# Application of the Theoretical Framework of Acceptability in healthcare interventions: a scoping review and proposed reporting checklist

**DOI:** 10.1186/s12913-026-15616-6

**Published:** 2026-09-14

**Authors:** Cara Ghiglieri, Benjamin McCullough, Rebecca H. McLeese, Brenda O’Neill, Emily Bannister, Bronwen Connolly, Judy M. Bradley

**Affiliations:** 1 Wellcome-Wolfson Institute for Experimental Medicine, School of Medicine, Dentistry and Biomedical Sciences, Queen’s University Belfast, Belfast, Northern Ireland UK; 2 School of Health Sciences, Faculty of Life & Health Sciences, Ulster University, Belfast, Northern Ireland UK; 3 Department of Physiotherapy, The University of Melbourne, Melbourne, Australia; 4 School of Medicine, Faculty of Life Sciences and Medicine, King’s College London, Portsmouth Campus, London, UK

**Keywords:** Theoretical Framework of Acceptability, Acceptability, Scoping review, Healthcare interventions, Implementation, TFAQ

## Abstract

**Background:**

The Theoretical Framework of Acceptability (TFA) assesses how interventions are perceived by those who deliver and receive them. However, there has been no systematic mapping of how the framework is conceptualised, operationalised, and reported in healthcare intervention research.

**Aim:**

To examine how the TFA is used to assess acceptability in healthcare intervention research.

**Methods:**

A scoping review was conducted using JBI methodology and reported in accordance with PRISMA-ScR. Searches were undertaken across MEDLINE, EMBASE, CINAHL, Cochrane Library, ClinicalTrials.gov and ISRCTN from 2017 to July 2025. Empirical studies, trial registrations, and published protocols were included if they applied the TFA to assess the acceptability of patient-facing healthcare interventions. Data were extracted and synthesised using descriptive and narrative approaches.

**Results:**

Use of the TFA has increased over time and spans diverse clinical contexts. When findings were aggregated across source types, acceptability was most commonly assessed, or planned to be assessed, using questionnaires and was typically positioned as a post-intervention outcome. Assessments were predominantly conducted with patients or service users at a single time point. Sensitivity analysis restricted to empirical studies showed that assessment was predominantly qualitative and interview-based, while outcome positioning and post-intervention timing remained common. Non-specification of TFA constructs was concentrated among trial registrations. In the overall sample, the TFA most frequently informed data collection, whereas among empirical studies it was most often applied during analysis, reporting and interpretation.

**Conclusion:**

Despite widespread adoption, application of the TFA remains variable and reporting does not always provide sufficient detail to support appraisal and comparison. This limits the ability to compare acceptability across studies, identify key drivers, or use findings to inform intervention development and implementation. Greater transparency in how acceptability is conceptualised, assessed and reported may enhance the utility of the TFA. A reporting checklist may support more consistent interpretation, clearer appraisal of framework use, and improved comparability across studies.

**Clinical trial number:**

Not applicable.

**Supplementary Information:**

The online version contains supplementary material available at https://doi.org/10.1186/s12913-026-15616-6.

## Background

Acceptability is an important consideration in the evaluation and implementation of healthcare interventions [1], as individuals are more likely to engage with and adhere to interventions perceived as acceptable [2]. Consequently, understanding how acceptability is conceptualised and assessed has become a key focus within intervention research, leading to the development of the Theoretical Framework of Acceptability (TFA) [3]. Within this framework, acceptability is defined as the extent to which individuals delivering or receiving an intervention perceive it to be appropriate, based on their anticipated or experienced cognitive and emotional responses to it [4]. The TFA conceptualises acceptability as a multi-component construct comprising seven domains: affective attitude, burden, ethicality, intervention coherence, opportunity costs, perceived effectiveness, and self-efficacy.

To support empirical application, the TFA has been operationalised through the development of the Theoretical Framework of Acceptability Questionnaire (TFAQ), a self-report measure designed to assess both prospective and retrospective acceptability [3]. The questionnaire includes items aligned with each of the seven constructs, alongside a general acceptability item that captures overall acceptability.

Since its development, the TFA has been used to explore how diverse healthcare interventions are perceived by patients, healthcare professionals and other stakeholders [5, 6]. However, despite its growing application within healthcare intervention research, no review has systematically mapped how the framework has been positioned and operationalised across studies. This may limit understanding of how acceptability evidence is generated, interpreted and applied, making it difficult to compare findings across studies or to use acceptability data to inform decisions about intervention refinement and implementation. It may also limit transparency in how the TFA is reported, making it difficult for readers to judge how the framework has informed study design, measurement, analysis and interpretation.

Given the need to explore and describe the literature rather than undertake more analytical synthesis [7], we conducted a scoping review. The aim of this review was to address the following research question: How is the TFA being used to inform the assessment of acceptability in healthcare intervention research?

## Methods

### Study design

This review was conducted using the JBI framework for scoping reviews [8] and adhered to the Preferred Reporting Items for Systematic Reviews and Meta-Analyses Extension for Scoping Reviews guidelines (PRISMA-ScR; Additional File 1) [9]. The protocol was registered a priori on the OSF database (https://doi.org/10.17605/OSF.IO/YZ4EG).

### Objectives

1. To identify studies that report using the TFA to assess acceptability in the context of healthcare interventions.
2. To examine how the TFA informs study design and methodology, including its influence on intervention development, data collection instruments (e.g. topic guides, surveys), analysis, or interpretation.
3. To explore how acceptability is conceptually defined and positioned within each study, including any theoretical justification.
4. To identify the specific tools and measures used to assess acceptability, including whether these were standardised (e.g. TFAQ), adapted from existing instruments, or developed for the study.
5. To examine how acceptability findings are reported, including their role in the study (e.g. outcome, process component), the type of data collected (qualitative, quantitative), and timing of assessment (anticipated, concurrent, retrospective).

### Eligibility criteria

Eligibility criteria were defined using the Population-Concept-Context framework [8]. Studies were included if they examined healthcare interventions directed at patients. Acceptability could be assessed among patients or other stakeholders (e.g. healthcare professionals, carers, or family members), where this related to delivering or supporting a patient-facing intervention. Studies were excluded if interventions targeted non-patient groups.

Eligible studies were required to demonstrate application of the TFA within the study method. Interventions had to be patient-facing and delivered within clinical or clinically supervised contexts. Studies were excluded if the TFA was only cited without application, or if interventions were population-level.

Any healthcare or clinically linked setting was eligible, with no geographic restrictions. Studies conducted outside healthcare settings without clinical involvement were excluded. Searches were limited to studies published in English due to resource constraints.

Empirical peer-reviewed studies using qualitative, quantitative or mixed-methods designs were included, alongside trial protocols and registrations reporting planned use of the TFA. Editorials, commentaries, conference abstracts, theses, grey literature, and systematic reviews were excluded due to limited methodological detail, which restricted the ability to assess how the TFA was applied across the research process. Reference lists or relevant reviews were screened for additional studies.

### Search strategy

A three-step search strategy was undertaken. First, an initial limited search of PubMed was conducted to identify relevant articles and to examine keywords and indexing terms used in titles and abstracts. These terms informed the development of the full search strategy. Second, the search strategy was developed in consultation with an academic librarian and adapted for each database. Search terms combined concepts relating to the TFA, healthcare interventions and approaches to assessing acceptability. The final search was conducted across MEDLINE (via PubMed and Ovid), EMBASE (via Ovid), CINAHL (via EBSCO), and the Cochrane Library from January 2017 (the first publication of the TFA) to 7 July 2025. ClinicalTrials.gov and the ISRCTN registry were also searched to identify protocols and ongoing studies. Third, the reference lists of included studies were screened to identify additional relevant records. Full search strategies for each database are provided in Additional File 2.

### Study selection

Search results were exported to EndNote 21 (Clarivate Analytics, PA, USA) and de-duplicated. Remaining records were imported into Covidence (Veritas Health Innovation, Melbourne, Australia) for screening. Title and abstract screening and full-text review were conducted independently by two reviewers following a pilot of 25 articles. Reasons for exclusion at the full-text stage were recorded. Disagreements were resolved through discussion, with a third reviewer consulted where necessary.

Where trial registrations, protocols, and published papers referred to the same underlying study, records were reviewed together to identify linked publications. Where linkages were confirmed, records were treated as multiple reports of a single study rather than as separate studies. Linked records were retained within the study selection counts reported in the PRISMA flow diagram.

### Data extraction

Consistent with scoping review methodology [8], no formal assessment of methodological quality or risk of bias was undertaken, as the aim was to map the extent and nature of the evidence rather than evaluate study quality.

Data were extracted from included sources by one reviewer and checked by a second reviewer using a standardised data extraction tool developed by the review team (Additional File 3). Discrepancies were resolved through discussion. Where multiple reports related to the same study, data were collated across reports and extracted as a single study-level entry.

The extraction tool (Additional File 3) was designed to capture both descriptive study characteristics and methodological details relating to the application of the TFA, with predefined operational definitions and coding guidance to support consistent classification. Extracted data included study identification details, study characteristics, description of the healthcare intervention and target population, and study setting. Additional variables captured how the TFA was utilised within each study (e.g. intervention design, data collection, analysis, interpretation, or reporting), methods used to assess acceptability, the TFA constructs applied, the positioning of acceptability within the study (e.g. outcome, process outcome, or combined), the type of data collected (qualitative, quantitative, or mixed methods), and the timing of assessment.

The extraction tool was piloted on a subset of 10 studies and refined to ensure clarity and consistency.

### Data synthesis and presentation

Extracted data were synthesised using descriptive and narrative approaches [8]. To support consistent synthesis, a data dictionary containing predefined operational definitions and coding rules was developed by the review team (Additional File 4). This was used to standardise classification across review variables, including study design, intervention type, timing of assessment, construct engagement, data collection tool and analytical approach. Predefined operational definitions and coding guidance for the stages of TFA application were embedded within the standardised extraction tool provided in Additional File 3. Illustrative examples from included studies were included to demonstrate how distinctions between analysis, interpretation and reporting were applied. Classification was based on explicit descriptions provided in study methods and supplementary materials. Where reporting was insufficient to support classification, studies were coded as unclear rather than framework use being inferred.

Descriptive statistics were used to summarise study characteristics, and results were presented in tabular form alongside a narrative synthesis describing how the TFA has been applied across healthcare intervention research.

Given the inclusion of empirical studies, protocols and trial registrations, a post hoc sensitivity analysis was conducted to examine the influence of source-type composition on the pooled findings. The variables reported in Tables 2 and 3 were recalculated after restricting the analysis to the 64 empirical studies.

Findings from the pooled synthesis and sensitivity analysis were used to inform the development of an evidence-informed reporting checklist. Candidate checklist items were derived from recurring patterns of variation, incomplete specification and limited transparency identified in the review findings and refined through discussion within the review team. Each checklist item was mapped to the specific pooled and empirical findings that informed its inclusion.

## Results

### Study selection

A total of 2,081 records were identified through database and registry searches. After removing 576 duplicates, 1505 records remained for title and abstract screening, of which 953 were excluded for not meeting the inclusion criteria. During screening, systematic reviews were identified and their reference lists were hand-searched, yielding 32 additional records.

**Fig. 1:**
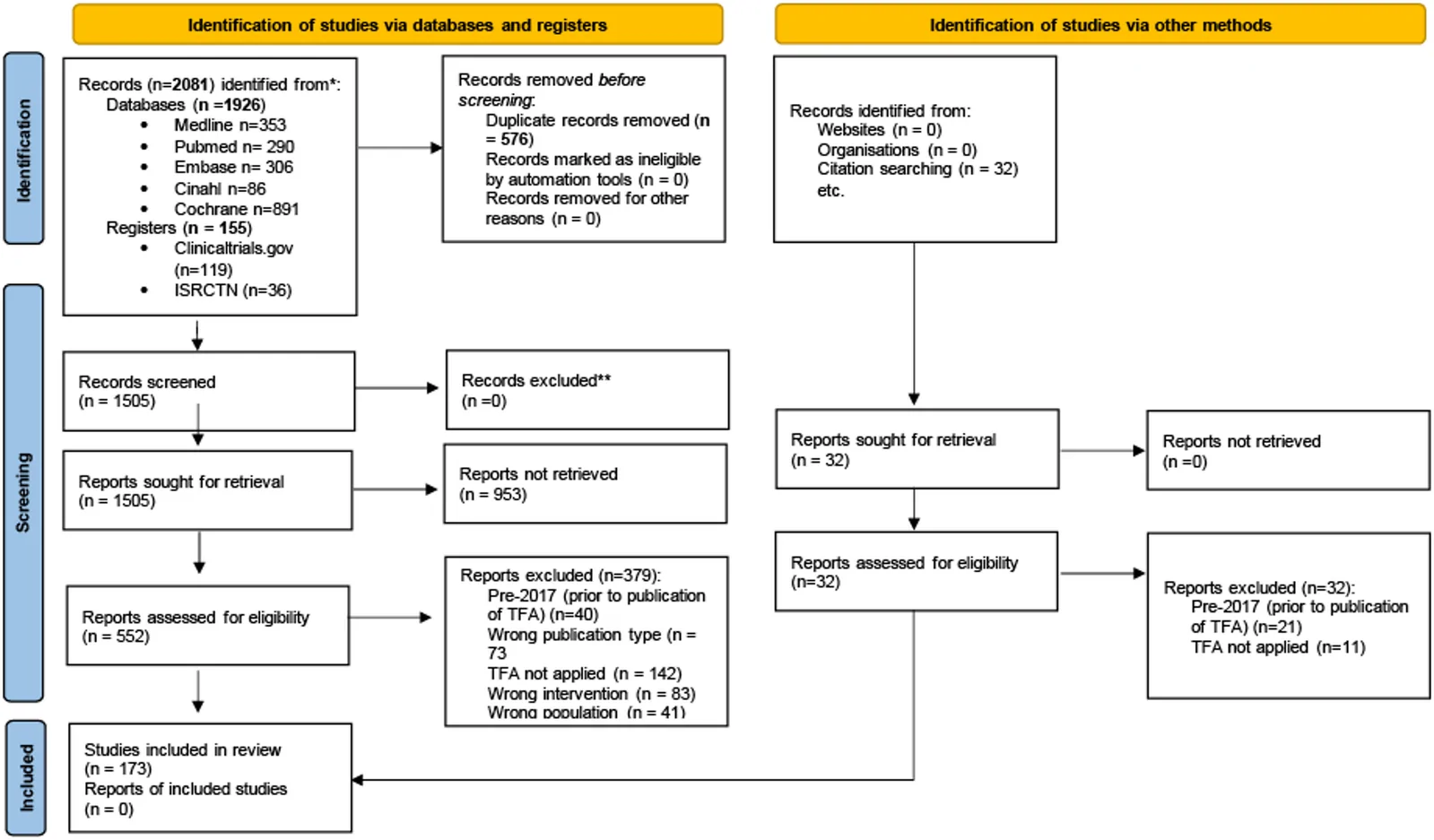
PRISMA flow diagram of study selection

In total, 584 records (552 from database and registry searches and 32 from hand-searching) underwent full-text review. Of these, 411 were excluded (Additional File 5) and 173 records met the inclusion criteria. Eight records were identified as linked reports of the same underlying study (Additional File 6), therefore, 165 unique study entries were included. The study selection process is summarised in Fig. 1.

### Study characteristics

#### Study overview and design

Characteristics of included studies are summarised in Table 1, with detailed study-level characteristics provided in Additional File 7. Of the 165 included records, 64 were empirical studies, 10 were study protocols, and 91 were trial registrations. As such, the findings reflect both reported and planned applications of the TFA. Use of the TFA increased over time, with most records published between 2024 and 2025 (86/165). Studies were conducted across six continents, with the largest number undertaken in Europe (77/165).

Randomised controlled trials were the most frequently reported study design (109/165), followed by quasi-experimental pre-post studies (31/165) and qualitative studies (17/165).

#### Intervention and participant characteristics

Interventions were delivered across a range of healthcare settings, most commonly in hospital outpatient settings (hospital-based services delivered without inpatient admission; 69/165) and community settings (delivered outside hospital environments, e.g. primary care, community health services, or home-based care; 57/165).

Intervention types varied widely. Behaviour change or self-management interventions were most common (38/165), followed by multicomponent interventions (33/165) and psychological interventions (31/165).

Interventions involved a wide range of clinical populations, most commonly oncology (30/165) and the majority involved adult populations (141/165).

**Table 1:** Characteristics of included studies reporting use of the Theoretical Framework of Acceptability (TFA) to assess acceptability of healthcare interventions

Category	Number of studies (*n* = 165)	Percentage (%)	Category	Number of studies (*n* = 165)	Percentage (%)
**Year of publication/registration**			**Study type**		
2017–2019	3	1.8%	Empirical study	64	38.8%
2020–2021	21	12.7%	Protocol	10	6.1%
2022–2023	55	33.3%	Trial registration	91	55.2%
2024–2025	86	52.1%	**Setting of intervention delivery**		
**Continent**			Outpatient	69	41.8%
Europe	77	46.7%	Community	57	34.5%
North America	37	22.4%	Inpatient	16	9.7%
Oceania	38	23.0%	Mixed settings	23	13.9%
Asia	7	4.2%	**Clinical population**		
Africa	5	3.0%	Cancer/oncology	30	18.2%
South America	1	0.6%	Musculoskeletal/rheumatology/chronic pain	24	14.5%
**Intervention type**			Neurological/cognitive	24	14.5%
Behaviour change/self-management	38	23.0%	Mental health	19	11.5%
Complementary therapy	2	1.2%	Cardiovascular/vascular	16	9.7%
Digital/monitoring	14	8.5%	Endocrine/metabolic	9	5.5%
Multicomponent	33	20.0%	Critical care/acute recovery	8	4.8%
Occupational therapy	2	1.2%	Infectious disease	5	3.0%
Pharmacological	11	6.7%	Respiratory	5	3.0%
Physical rehabilitation	23	13.9%	Maternal/reproductive	4	2.4%
Psychological	31	18.8%	Multimorbidity	4	2.4%
Speech and language therapy	5	3.0%	Geriatric/frailty	4	2.4%
Surgical/procedural	6	3.6%	Neurodevelopmental	3	1.8%
**Intervention design**			Congenital/structural	2	1.2%
Randomised controlled trial	109	66.1%	Ophthalmology/vision	2	1.2%
Quasi-experimental (pre-post)	31	18.8%	Urological	2	1.2%
Other quasi-experimental (other)	2	1.2%	Dermatology/ skin	2	1.2%
Cross-sectional study	3	1.8%	**Age group**		
Cohort study	3	1.8%	Adult populations	141	85.5%
Standalone qualitative study	17	10.3%	Paediatric populations	15	9.1%
			Mixed-age populations	9	5.5%

#### Application of the TFA

This section presents the pooled findings on how the TFA was applied across all 165 study entries, comprising empirical studies, protocols and trial registrations. Detailed study-level data are provided in Additional File 8. Findings from the sensitivity analysis restricted to empirical studies are presented after the pooled synthesis, with full results provided in Additional File 9.

#### Conceptualisation, assessment and engagement of acceptability

The conceptualisation and measurement of acceptability using the TFA across included studies (*n* = 165) are summarised in Table 2. A range of methods were used, or planned, to assess acceptability. Questionnaires were most common (71/165), followed by interviews (59/165) and combined approaches using more than one method (32/165). Focus groups were rarely used as a standalone approach (1/165), and in two trial registrations the method of assessment was not reported.

Among studies using questionnaires (*n* = 71), most employed quantitative approaches (52/71), with limited use of qualitative or mixed-method survey designs. Combined approaches (*n* = 32) most commonly involved the use of both questionnaires and interviews (17/32), followed by combinations of interviews and focus groups (10/32). Less frequently, studies combined questionnaires with focus groups (3/32), and two studies used all three methods.

In terms of the type of TFA data collected, qualitative approaches were most common (76/165), followed by quantitative approaches (52/165) and mixed methods (21/165). In 16 trial registrations, the type of data collected was not reported; most of these involved questionnaire-based approaches where the analytic approach was unclear.

Acceptability was most commonly assessed among patients or service users only (102/165), followed by studies including multiple stakeholder groups (54/165). Of the studies including multiple stakeholder groups, over half involved both patients and healthcare professionals (31/54).

Acceptability was predominantly positioned as an outcome of intervention exposure (118/165), with fewer studies conceptualising it as a process (27/165) or as both an outcome and a process (20/165). Among studies conceptualising acceptability as a process (*n* = 27), most used qualitative methods (18/27).

Post-intervention-only assessment was the most common timing category (116/165). Assessment occurred before the intervention only in 6/165 study entries and during intervention delivery only in 7/165. In 31/165 study entries, acceptability was assessed or planned to be assessed at more than one stage: pre- and post-intervention (13/165), during and post-intervention (6/165), post-intervention and follow-up (3/165), or across three or more stages (9/165). Timing was not reported in five study entries.

Reporting of construct engagement varied across studies. In 66/165 studies, the TFA constructs assessed or planned for assessment were not clearly specified. In contrast, 58/165 studies reported engaging with all seven constructs and a further 18/165 included an additional overall acceptability measure alongside the full construct set. A smaller number of studies engaged with a subset of constructs rather than the full framework (22/165) and in one case an additional construct was introduced. Among these studies (*n* = 22), ethicality was most frequently omitted (15/22), followed by opportunity cost (10/22). Most studies using selective constructs employed qualitative methods to assess acceptability (15/22).

**Table 2:** Conceptualisation, assessment and engagement of acceptability across included studies

Domain	Number of studies (*n* = 165)	Percentage (%)	Domain	Number of studies (*n* = 165)	Percentage (%)
**Type of TFA data collected**			**Population (TFA-measured)**		
Qualitative	76	46.1%	Patients / service users only	102	61.8%
Quantitative	52	31.5%	Healthcare professionals	6	3.6%
Mixed methods	21	12.7%	Carers / informal support only	3	1.8%
Not reported	16	9.7%	Multiple stakeholder groups	54	32.7%
**Method used to assess acceptability**			**Positioning of acceptability**		
Questionnaire	71	43.0%	Outcome	118	71.5%
Interviews	59	35.8%	Process outcome	27	16.4%
Combined	32	19.4%	Combined (outcome + process)	20	12.1%
Focus groups	1	0.6%	**Timing of assessment**		
Not reported	2	1.2%	Pre-intervention	6	3.6%
**Construct engagement**			During intervention	7	4.2%
Full set of 7 constructs	58	35.2%	Post-intervention	116	70.3%
Full + overall acceptability	18	10.9%	Pre + Post	13	7.9%
Selective use	22	13.3%	During + Post	6	3.6%
Additional constructs	1	0.6%	Post + follow-up	3	1.8%
Not specified	66	40.0%	Multiple stages (3+)	9	5.5%
			Not reported	5	3.0%

#### Application of the TFA across the research process

The application of the TFA across stages of the research process, and how it was operationalised within these stages, are summarised in Table 3. Across the included studies (*n* = 165), the TFA was predominantly applied during data collection (125/165), followed by analysis (75/165), reporting (61/165), interpretation (56/165), and study design (32/165), whilst 5 trial registrations did not explicitly state the stage of application. These categories were not mutually exclusive, as many studies applied the TFA at multiple stages.

Among studies that used the TFA to inform the design of data collection tools (*n* = 125), questionnaires (67/125) and interview topic guides (39/125) were most commonly used. This reflects a subset of studies in which the TFA explicitly shaped how data were collected, rather than studies that used these methods more generally. Eighteen studies used the TFA to inform multiple tools within the same study and one study applied it to a focus group topic guide. Among questionnaire-based approaches, adapted or study-specific measures were more commonly used than standardised instruments (42/67 vs. 25/67).

Among studies that used the TFA to inform analysis (75/165), qualitative approaches were most common (57/75), followed by mixed methods (10/75) and quantitative approaches (8/75). Within qualitative studies, 31/57 used deductive analysis structured by TFA constructs, while 26/57 employed an inductive-deductive approach, whereby themes were generated inductively and subsequently mapped to the framework. Quantitative studies most commonly used descriptive analyses (6/8), with fewer using inferential approaches (2/8). Mixed-methods studies either integrated qualitative and quantitative findings (4/10) or analysed them in parallel (6/10).

**Table 3:** Application of the TFA across stages of the research process and its operationalisation within studies

Stage of TFA application	Number of studies	Percentage (%)
**TFA used to inform study design**	**32/165**	**19.4%**
**TFA used to inform data collection**	**125/165**	**75.8%**
Interview guides informed by the TFA	39/125	31.2%
Focus group topic guides informed by the TFA	1/125	0.8%
Study-specific questionnaires informed by the TFA	42/125	33.6%
Standardised TFAQ	25/125	20.0%
Multiple data collection tools informed by the TFA	18/125	14.4%
**TFA used to inform analysis**	**75/165**	**45.5%**
Deductive qualitative analysis	31/75	41.3%
Inductive-deductive qualitative analysis	26/75	34.7%
Descriptive quantitative analysis	6/75	8.0%
Inferential quantitative analysis	2/75	2.7%
Parallel mixed-methods analysis	6/75	8.0%
Integrated mixed-methods analysis	4/75	5.3%
**TFA used to inform interpretation**	56/165	33.9%
**TFA used to structure reporting**	61/165	37.0%
**Unclear - Not reported where TFA applied**	5/165	3.0%

* Percentages for subcategories are calculated using the number of studies within each stage (e.g. data collection, analysis) as the denominator. Categories are not mutually exclusive. Full operational definitions and coding guidance for the stages of TFA application and their data collection and analysis subcategories are provided in Additional Files 3 and 4

#### Sensitivity analysis restricted to empirical studies

Restricting the analysis to the 64 empirical studies showed that several pooled patterns were influenced by source-type composition. Empirical studies predominantly collected qualitative TFA data (49/64, 76.6%) and most commonly assessed acceptability using interviews alone (38/64, 59.4%). A further 17/64 (26.6%) used combined assessment methods, while 9/64 (14.1%) used questionnaires alone.

The predominance of patient or service-user perspectives remained evident. Patients or service users were the only stakeholder group assessed in 37/64 empirical studies (57.8%), while 21/64 (32.8%) included multiple stakeholder groups. Six empirical studies assessed healthcare professionals alone, and none assessed carers or informal support providers alone.

Acceptability remained most frequently positioned as an outcome (42/64, 65.6%), followed by a process outcome (15/64, 23.4%) and a combination of outcome and process (7/64, 10.9%). Outcome positioning therefore remained predominant, although process-outcome positioning was more frequent among empirical studies than in the pooled analysis (15/64, 23.4% versus 27/165, 16.4%).

Post-intervention-only assessment also remained the most common timing category, occurring in 46/64 empirical studies (71.9%), compared with 116/165 study entries (70.3%) in the pooled analysis.

Construct engagement was more completely specified among empirical studies. Only 1/64 empirical studies (1.6%) did not report which constructs were assessed. Of the 66 study entries in the pooled analysis in which construct engagement was not specified, 62 were trial registrations, three were protocols and one was an empirical study. Among empirical studies, 38/64 (59.4%) used all seven constructs, 10/64 (15.6%) used all seven constructs alongside an overall acceptability measure and 14/64 (21.9%) used selected constructs.

Patterns of TFA application across the research process also differed. Among empirical studies, the TFA was most frequently used to inform analysis (60/64, 93.8%), structure reporting (57/64, 89.1%) and inform interpretation (48/64, 75.0%). It informed data collection in 34/64 studies (53.1%) and study design in 26/64 (40.6%). Full findings from the sensitivity analysis are presented in Additional File 9.

## Discussion

This scoping review examined how the TFA has been applied within healthcare intervention research. Across 165 unique study entries, uptake of the framework has increased across a wide range of clinical areas and study designs. However, substantial variation was identified in how the framework is conceptualised, operationalised and integrated across the research process.

Acceptability was predominantly assessed from the perspective of patients, with more limited inclusion of healthcare professionals and minimal attention to carers. Although broadly consistent with the original emphasis of the TFA on intervention recipients and deliverers [4], this may limit understanding in contexts where delivery, uptake and sustainment depend on multiple stakeholders. Many healthcare interventions are enacted across networks of patients, carers and professionals [10, 11], and the acceptability of an intervention to one group may shape the experience, engagement, or burden of another [12]. Clear reporting of whose perspectives are included and why is therefore important for interpreting how acceptability is understood within a given study.

The combined predominance of post-intervention assessment and outcome positioning suggests that acceptability is commonly approached as a retrospective evaluation of intervention exposure, rather than as a dynamic process that may shape engagement throughout an intervention. This is notable given that the TFA was developed to capture anticipated, concurrent and experienced acceptability [4]. Where assessment is reported only at the end of an intervention, it is difficult to determine how expectations influence recruitment, how experiences shape continued engagement or how acceptability evolves across the intervention lifecycle [13, 14]. Greater attention to prospective and concurrent assessment would support a more temporally sensitive understanding of acceptability.

Differences between the pooled and empirical analyses also illustrate the importance of distinguishing intended from implemented framework use. Trial registrations and protocols frequently described planned TFA-informed questionnaires or topic guides, whereas completed empirical studies more often used the framework during analysis, reporting and interpretation. Similarly, the apparent predominance of questionnaire-based assessment in the pooled synthesis was influenced by planned assessment within registrations and protocols, while empirical studies were primarily qualitative and interview-based. Neither qualitative nor quantitative assessment is inherently more appropriate; their value depends on the study purpose and the form of acceptability evidence required. While theory-informed data collection and analysis can support analytic coherence [15], limited integration early in the research process may constrain how the framework contributes to intervention development and evaluation [16]. The more important issue is therefore whether the TFA’s influence can be traced across the research process, including how constructs shaped the questions asked, the analysis undertaken and the conclusions drawn. Without this transparency, it is difficult to determine whether the framework meaningfully informed the research or was applied only at an isolated stage, and to compare how it was operationalised across studies [17].

The sensitivity analysis further showed that construct non-specification was largely concentrated among trial registrations. The more substantive issue within empirical studies was therefore not simply whether constructs were named, but whether selective construct use was justified and clearly operationalised. In some contexts, omission of constructs such as ethicality or opportunity cost may reflect perceived lack of relevance or practical difficulties in interpretation [18]. During development of the TFAQ, participants experienced difficulties interpreting or responding to some ethicality items, suggesting that their relevance and wording may vary across intervention contexts [3]. Opportunity cost also presented measurement challenges, and Paynter et al. have argued that its original definition may be overly unipolar because it foregrounds what individuals relinquish while overlooking potential gains from participation [18]. However, studies rarely explained why particular constructs were excluded. Selective use may be appropriate, but without an explicit rationale it becomes difficult to distinguish theoretically informed adaptation from incomplete application of the framework. In practice, this is likely to limit comparison across studies, interpretation of findings for intervention or implementation decisions and the cumulative development of knowledge about acceptability [19].

These patterns constrain the extent to which TFA evidence can contribute cumulatively to intervention development and implementation. The TFAQ represents an important step towards more structured and theory-informed measurement [3], but shared approaches to interpreting construct-level and overall acceptability scores remain underdeveloped. This reflects the relative maturity of the evidence base rather than an inherent limitation of the questionnaire. Although many theoretical measures are not interpreted using fixed thresholds, conventions for understanding patterns of scores often develop through repeated application and comparison [20]. At present, acceptability can be described within individual studies, but findings cannot readily be interpreted in a consistent way across interventions and settings.

It is also not yet possible to determine robustly whether particular TFA constructs are more influential than others in shaping uptake, engagement, fidelity or intervention effectiveness. This requires consistent construct specification, transparent operationalisation and clearer linkage between acceptability findings and relevant behavioural or implementation outcomes [14]. Without these connections, the framework can describe how an intervention is perceived but offers more limited insight into which aspects of acceptability matter most, for whom and under what conditions. More broadly, future TFA research should examine how individual constructs relate to intervention engagement and outcomes, develop clearer approaches to interpreting TFAQ findings and investigate acceptability across multiple stakeholder perspectives and stages of intervention delivery.

To support clearer and more consistent application of the TFA, we developed an evidence-informed reporting checklist grounded in the pooled and empirical findings of this review. Each item was mapped to the findings that informed its inclusion, providing a transparent basis for the proposed recommendations (Additional File 10). Deriving proposed items from a review of the available evidence reflects an early evidence-informed component of recommended processes for developing health research reporting guidelines [21]. The checklist is intended to help researchers, reviewers and readers trace how acceptability has been conceptualised, assessed, analysed and interpreted, thereby supporting critical appraisal and comparison across studies (Table 4). However, it has not undergone stakeholder consultation, formal consensus development or pilot testing and should therefore be regarded as a proposed reporting tool requiring further refinement and evaluation.

**Table 4:** Evidence-informed reporting checklist for studies applying the Theoretical Framework of Acceptability in healthcare intervention research

Item to report	Description of what to report	Rationale
Conceptualisation of acceptability	Explain the purpose of using the TFA and how its use relates to the study aim, intervention context and positioning of acceptability.	The TFA was used for different purposes and in different ways across studies. Explaining why the framework was selected and how it relates to the study aim and context helps readers interpret subsequent decisions about positioning, timing, construct selection, measurement and application across the research process.
Positioning of acceptability	State whether acceptability is treated as an outcome, a process, or both	How acceptability is positioned shapes how findings are interpreted; making this explicit helps clarify whether the study captures static evaluations, dynamic experiences, or both
Stage of integration	Specify at which stages the TFA is applied (design, data collection, analysis, interpretation, reporting)	Transparent reporting of where the framework is applied enables readers to assess how it has informed the study and whether its use is integrated or limited to specific stages
Stakeholder inclusion	Report whose perspectives are assessed (e.g. recipients, deliverers, others) and justify inclusion	Acceptability is inherently multi-perspectival; clearly reporting whose views are included supports a fuller understanding of how interventions are experienced and implemented
Timing of assessment	Report when acceptability is assessed (pre, during, post-intervention, follow-up) and provide rationale	The timing of assessment shapes what is captured; reporting this enables readers to interpret whether findings reflect initial expectations, evolving experiences, or retrospective evaluations
Construct selection	Specify which TFA constructs are included and justify any omissions, additions or adaptations.	Transparent reporting of construct selection allows readers to understand the scope of assessment and whether acceptability has been examined comprehensively or selectively
Operationalisation	Describe how TFA constructs informed data collection tools and study procedures	Clear mapping between constructs and methods supports conceptual clarity and enables readers to assess how constructs have been translated into practice
Measurement approach	Report the measures used (e.g. TFAQ, adapted tools) and how they were developed or selected	Transparency in measurement allows readers to understand how acceptability was assessed, appraise the suitability of the selected approach and compare methods across studies.
Analytical approach	Describe how the TFA informed analysis (e.g. deductive, inductive–deductive, quantitative)	Explicit reporting of analytic approach enables assessment of rigour and clarifies how the framework shaped interpretation of the data
Interpretation and application	Explain how acceptability findings are interpreted in relation to intervention refinement, implementation, or conclusions	Making this link explicit supports the applied value of acceptability research and clarifies how findings contribute to intervention development or implementation decisions
Reporting transparency	Provide sufficient detail to trace how the TFA informed each relevant stage of the study, including any differences between planned and implemented use.	Complete reporting enables readers to distinguish intended from implemented framework use and supports critical appraisal, replication and comparison across studies.

Note: The specific pooled and empirical findings underpinning each checklist item are detailed in Additional File 10. The checklist was derived from the findings of this review and has not undergone formal consensus testing or external validation

### Strengths and limitations

This review has several strengths. It was conducted in line with established methodological guidance for scoping reviews [8], ensuring a systematic and transparent approach to study identification, selection, data extraction, and synthesis. By including empirical studies alongside protocols and trial registrations, it captures how the TFA is positioned and applied both in completed applications and intended prospective use. The review also examined multiple dimensions of framework use, including conceptualisation, timing, measurement, analytic approach and construct engagement, allowing a broad view of how the TFA is currently applied in practice.

Several limitations should be acknowledged. The inclusion of empirical studies, protocols and trial registrations was intentional, as the review sought to capture both planned and reported applications of the TFA. However, these source types differ in their purpose and level of methodological detail, meaning that pooled findings may reflect source-type composition as well as variation in TFA application. A post hoc sensitivity analysis restricted to the 64 empirical studies was therefore conducted. This demonstrated that some patterns, particularly construct non-specification and the apparent predominance of questionnaire-based assessment, were strongly influenced by the inclusion of registrations and protocols. In contrast, the predominance of outcome positioning and post-intervention assessment remained evident within empirical studies. Classification of framework use nevertheless relied on the information reported within each source, and the extent to which the TFA informed study methods may therefore have been underestimated. Although coding decisions were made systematically, some degree of subjectivity is inherent in this process.

Consistent with the aims of a scoping review, methodological quality was not assessed, and the review did not examine how different approaches to applying the TFA influence study outcomes. Searches were restricted to English-language publications due to resource constraints. This may have introduced geographic and cultural bias by excluding relevant studies published in other languages and may have limited identification of culturally diverse applications of the TFA, particularly from regions that were less well represented within the included literature.

As all included studies explicitly applied the TFA, the review did not seek to compare competing definitions of acceptability; instead, it focused on variation in how the framework was positioned, operationalised and reported. Finally, the proposed checklist was developed from patterns identified in the pooled and empirical findings, with the derivation of each item detailed in Additional File 10. It has not been formally consensus-tested or externally validated and should therefore be considered a proposed, evidence-informed tool to support reporting transparency rather than a definitive reporting standard.

## Conclusions

This review shows that application of the TFA varies across source types and stages of the research process. Trial registrations and protocols commonly described planned questionnaire-based assessment, whereas empirical studies predominantly used qualitative interviews and applied the TFA during analysis, reporting and interpretation. Outcome positioning and post-intervention assessment remained common among empirical studies. More transparent and consistent reporting of how the TFA is conceptualised, operationalised and interpreted may strengthen its use in healthcare intervention research. The proposed reporting checklist is grounded in the findings of this review and may support clearer appraisal, comparison and cumulative learning across studies.

## Supplementary Information

Below is the link to the electronic supplementary material.

Supplementary Material 1

## Data Availability

The datasets generated and/or analysed during the current study are available from the corresponding author on reasonable request.

## Abbreviations

TFA: Theoretical framework of acceptability
TFAQ: Theoretical framework of acceptability questionnaire
PRISMA-ScR: Preferred reporting items for systematic reviews and meta-analyses extension for scoping reviews
JBI: Joanna briggs institute
