# Supplementary material for: Application of the Theoretical Framework of Acceptability in healthcare interventions: a scoping review and proposed reporting checklist
Source: BMC Health Serv Res. 2026 Sep 14;26:1306. doi: 10.1186/s12913-026-15616-6 (PMC13613629; doi:10.1186/s12913-026-15616-6)
Supplement: Supplementary file 1 [file 12913_2026_15616_MOESM1_ESM.docx]

**Additional files**

**Additional file 1:** PRISMA-ScR Checklist
**Additional file 2:** Search strategies
**Additional file 3:** Standardised extraction tool
**Additional file 4:** Data dictionary for categorisation
**Additional file 5:** Excluded studies
**Additional file 6:** Linked records
**Additional file 7:** Detailed study characteristics
**Additional file 8:** Detailed study-level data relating to the application of the TFA
**Additional file 9:** Sensitivity analysis restricted to empirical studies
**Additional file 10:** Mapping of review findings to the proposed TFA reporting checklist

# Additional file 1: Preferred Reporting Items for Systematic reviews and Meta-Analyses extension for Scoping Reviews (PRISMA-ScR) Checklist

| **SECTION** | **ITEM** | **PRISMA-ScR CHECKLIST ITEM** | **REPORTED ON PAGE #** |
| --- | --- | --- | --- |
| **TITLE** | | | |
| Title | 1 | Identify the report as a scoping review. | 1 |
| **ABSTRACT** | | | |
| Structured summary | 2 | Provide a structured summary that includes (as applicable): background, objectives, eligibility criteria, sources of evidence, charting methods, results, and conclusions that relate to the review questions and objectives. | 2 |
| **INTRODUCTION** | | | |
| Rationale | 3 | Describe the rationale for the review in the context of what is already known. Explain why the review questions/objectives lend themselves to a scoping review approach. | 3 |
| Objectives | 4 | Provide an explicit statement of the questions and objectives being addressed with reference to their key elements (e.g., population or participants, concepts, and context) or other relevant key elements used to conceptualize the review questions and/or objectives. | 4 |
| **METHODS** | | | |
| Protocol and registration | 5 | Indicate whether a review protocol exists; state if and where it can be accessed (e.g., a Web address); and if available, provide registration information, including the registration number. | 4 |
| Eligibility criteria | 6 | Specify characteristics of the sources of evidence used as eligibility criteria (e.g., years considered, language, and publication status), and provide a rationale. | 4 |
| Information sources* | 7 | Describe all information sources in the search (e.g., databases with dates of coverage and contact with authors to identify additional sources), as well as the date the most recent search was executed. | 5 |
| Search | 8 | Present the full electronic search strategy for at least 1 database, including any limits used, such that it could be repeated. | 5 |
| Selection of sources of evidence† | 9 | State the process for selecting sources of evidence (i.e., screening and eligibility) included in the scoping review. | 5 |
| Data charting process‡ | 10 | Describe the methods of charting data from the included sources of evidence (e.g., calibrated forms or forms that have been tested by the team before their use, and whether data charting was done independently or in duplicate) and any processes for obtaining and confirming data from investigators. | 6 |
| Data items | 11 | List and define all variables for which data were sought and any assumptions and simplifications made. | 6 |
| Critical appraisal of individual sources of evidence§ | 12 | If done, provide a rationale for conducting a critical appraisal of included sources of evidence; describe the methods used and how this information was used in any data synthesis (if appropriate). | n/a |
| Synthesis of results | 13 | Describe the methods of handling and summarizing the data that were charted. | 6 |
| **RESULTS** | | | |
| Selection of sources of evidence | 14 | Give numbers of sources of evidence screened, assessed for eligibility, and included in the review, with reasons for exclusions at each stage, ideally using a flow diagram. | 6 |
| Characteristics of sources of evidence | 15 | For each source of evidence, present characteristics for which data were charted and provide the citations. | 7-8 |
| Critical appraisal within sources of evidence | 16 | If done, present data on critical appraisal of included sources of evidence (see item 12). | N/A |
| Results of individual sources of evidence | 17 | For each included source of evidence, present the relevant data that were charted that relate to the review questions and objectives. | 7-13 |
| Synthesis of results | 18 | Summarize and/or present the charting results as they relate to the review questions and objectives. | 7-13 |
| **DISCUSSION** | | | |
| Summary of evidence | 19 | Summarize the main results (including an overview of concepts, themes, and types of evidence available), link to the review questions and objectives, and consider the relevance to key groups. | 13-14 |
| Limitations | 20 | Discuss the limitations of the scoping review process. | 17 |
| Conclusions | 21 | Provide a general interpretation of the results with respect to the review questions and objectives, as well as potential implications and/or next steps. | 18 |
| **FUNDING** | | | |
| Funding | 22 | Describe sources of funding for the included sources of evidence, as well as sources of funding for the scoping review. Describe the role of the funders of the scoping review. | 19 |

JBI = Joanna Briggs Institute; PRISMA-ScR = Preferred Reporting Items for Systematic reviews and Meta-Analyses extension for Scoping Reviews.

* Where *sources of evidence* (see second footnote) are compiled from, such as bibliographic databases, social media platforms, and Web sites.

† A more inclusive/heterogeneous term used to account for the different types of evidence or data sources (e.g., quantitative and/or qualitative research, expert opinion, and policy documents) that may be eligible in a scoping review as opposed to only studies. This is not to be confused with *information sources* (see first footnote).

‡ The frameworks by Arksey and O’Malley (6) and Levac and colleagues (7) and the JBI guidance (4, 5) refer to the process of data extraction in a scoping review as data charting*.*

§ The process of systematically examining research evidence to assess its validity, results, and relevance before using it to inform a decision. This term is used for items 12 and 19 instead of "risk of bias" (which is more applicable to systematic reviews of interventions) to include and acknowledge the various sources of evidence that may be used in a scoping review (e.g., quantitative and/or qualitative research, expert opinion, and policy document).

*From:* Tricco AC, Lillie E, Zarin W, O'Brien KK, Colquhoun H, Levac D, et al. PRISMA Extension for Scoping Reviews (PRISMAScR): Checklist and Explanation. Ann Intern Med. 2018;169:467-473. [doi: 10.7326/M18-0850](http://annals.org/aim/fullarticle/2700389/prisma-extension-scoping-reviews-prisma-scr-checklist-explanation).

# Additional file 2: Search strategies

A three-step search strategy was developed in line with Joanna Briggs Institute guidance. An initial limited search was conducted in PubMed to identify relevant articles and extract key terms and indexing. These terms informed the development of a comprehensive search strategy, which was iteratively refined and adapted for each database.

The search strategy combined terms relating to:

1. The Theoretical Framework of Acceptability (TFA)
2. Healthcare interventions
3. Methods used to assess acceptability

Full search strategies for each database are presented below.

**Full search terms for each database:**

| **Database** | **Search Date** | **Search String** | **Records Retrieved** |
| --- | --- | --- | --- |
| **MEDLINE (via Ovid)** | 07/07/2025 | ( “Theoretical Framework of Acceptability”.mp. OR TFA.mp. OR (theoretic*.tw. AND framework*.tw. AND accept*.tw.) ) AND ( intervention*.tw. OR “healthcare intervention”.tw. OR “clinical trial”.tw. OR “programme evaluation”.tw. OR “process evaluation”.tw. ) AND ( “acceptability testing”.tw. OR “acceptability questionnaire”.tw. OR TFAQ.tw. OR survey.tw. OR “topic guide”.tw. OR interview.tw. OR questionnaire.tw. OR “theory-informed”.tw. OR “framework-informed”.tw. ) | 353 |
| **EMBASE (via Ovid)** | 07/07/2025 | ("theoretical framework of acceptability".ti,ab. OR TFA.ti,ab. OR ((theoretic* OR theoretical) adj3 (framework* adj3 acceptab*)).ti,ab.) AND (intervention*.ti,ab. OR "health care intervention*".ti,ab. OR "clinical trial*".ti,ab. OR "programme evaluation*".ti,ab. OR "process evaluation*".ti,ab.) AND ("acceptability questionnaire".ti,ab. OR "acceptability testing".ti,ab. OR TFAQ.ti,ab. OR survey*.ti,ab. OR interview*.ti,ab. OR "topic guide".ti,ab. OR "theory-informed".ti,ab. OR "framework-informed".ti,ab. OR "mixed methods".ti,ab.) | 306 |
| **PubMed** | 07/07/2025 | (“Theoretical Framework of Acceptability”[All Fields] OR “TFA”[All Fields] OR ((“theoretical”[All Fields] OR “theoretically”[All Fields]) AND (“framework”[All Fields] OR “frameworks”[All Fields]) AND (“acceptability”[All Fields] OR “acceptable”[All Fields] OR “acceptance”[All Fields]))) AND (“intervention”[All Fields] OR “healthcare intervention”[All Fields] OR “clinical trial”[All Fields] OR “programme evaluation”[All Fields] OR “process evaluation”[All Fields]) AND (“acceptability testing”[All Fields] OR “acceptability questionnaire”[All Fields] OR “TFAQ”[All Fields] OR “survey”[All Fields] OR “topic guide”[All Fields] OR “interview”[All Fields] OR “questionnaire”[All Fields] OR “theory-informed”[All Fields] OR “framework-informed”[All Fields]) | 290 |
| **CINAHL (via EBSCO)** | 07/07/2025 | ("theoretical framework of acceptability" OR TFA OR ((theoretical OR theoretic*) N3 (framework* N3 acceptab*)))  AND (intervention* OR "healthcare intervention*" OR "clinical trial*" OR "programme evaluation*" OR "process evaluation*") AND ("acceptability questionnaire" OR "acceptability testing" OR TFAQ OR survey* OR interview* OR "topic guide" OR "theory-informed" OR "framework-informed" OR "mixed methods") | 86 |
| **Cochrane Library** | 07/07/2025 | (“Theoretical Framework of Acceptability” OR TFA OR (theoretic* AND framework* AND accept*)) AND (intervention OR “healthcare intervention” OR “process evaluation” OR “programme evaluation”) | 354  (reviews)  And  537  (trials) |
| **ClinicalTrials.gov** | 07/07/2025 | "Theoretical Framework of Acceptability" OR TFAQ | 119 |
| **ISRCTN Registry** | 07/07/2025 | "Theoretical Framework of Acceptability" | 36 |

# Additional file 3: Standardised extraction tool

**Purpose:**
This document defines all variables used in the data extraction form for the scoping review. It provides standardised definitions, response options, and decision rules to ensure transparent and reproducible data extraction across reviewers.

**General guidance:**

- Where “Free text” is listed, enter verbatim wording where possible.
- Capture only *explicit* mentions for each field; inferred use can be noted in the a*dditional comments* column.
- For guidance on handling linked papers or trial registrations, refer to the separate document on linking procedure.

**Reviewer responsibilities and quality checks:**

- Each study should be extracted by one reviewer and independently checked by a second.
- Minor discrepancies should be discussed directly between reviewers. Unresolved issues should involve third-party review.
- If recurring uncertainties arise, please notify the lead author as soon as possible so that the data dictionary can be updated for consistency.

| **Field** | **Response option** | **Response type** | **Description / example** | **Notes for consistency** |
| --- | --- | --- | --- | --- |
| **Linked paper** | Yes - specify | Free text | Indicate whether a publication linked to this registration was identified. Record as:  Linked to [Author surname] et al., [Year] | Use only when a linked publication is explicitly listed in the *Publications* or *Related studies* section of the trial registry. |
|  | None identified | Single choice | Indicate when no linked publication (e.g. protocol, results paper) is listed in the registration record. | Use when no linked document is listed within the *Publications* or *Related studies* section of the registration. |
| **Country of study** | - | Free text | Country (or countries) in which the study was conducted. | Use official country names (e.g. “United Kingdom,” not “UK”). For multi-country studies, list all countries separated by commas. |
| **Study type** | Published | Single choice | Peer-reviewed published article | Select when full peer-reviewed paper |
|  | Protocol |  | Published study protocol (e.g. registered or journal protocol) | Select when protocol is published/registered as a protocol |
|  | Registration |  | Trial registration or record (e.g. ClinicalTrials.gov, ISRCTN, ANZCTR). | Select for registered trials |
| **Study design/methods** | - | Free text | Short, high-level description of the *overall* study design and primary methods. | Use to give context for the study as a whole, not a detailed methodology and not just the acceptability strand. Include one short phrase or sentence that includes only:   1. **Design label -** e.g. RCT, quasi-experimental, open feasibility study, qualitative process evaluation, mixed-methods, etc. 2. **Very high-level features of the design -** e.g. feasibility study with repeated measures 3. **Primary data collection methods** (overall study, not just acceptability) - e.g. questionnaires, routine clinical data, interviews, focus groups with patients/carers/hcps   **Examples:**  “Quasi-experimental open feasibility study of guided self-help, with repeated questionnaire assessments at baseline and follow-up”  “Mixed-methods implementation study combining routine service data with interviews and focus groups with patients and healthcare professionals” |
| **Description of the healthcare intervention** | - | Free text | Summary of the intervention under study, including main components, duration, delivery mode, and intended purpose. | Provide concise summary (1-2 lines). Focus on what was delivered and how. |
| **Target population** | - | Free text | Group targeted by the intervention | Use population descriptors as stated by authors where possible. Avoid restating inclusion criteria in full. |
| **Setting** | Inpatient | Single choice | Participants receive the intervention while admitted to hospital (e.g. post-surgery ward, rehabilitation unit, ICU, oncology ward). | Select if delivery occurs in an inpatient clinical environment. |
|  | Outpatient |  | Participants attend hospital or specialist clinics for follow-up or treatment but are not admitted. | Select if delivery includes outpatient clinics, day units, or hospital-based telehealth. |
|  | Community |  | Intervention delivered outside hospital settings, such as GP surgeries, local health centres, community mental health teams, or voluntary-sector services | Select for community- or primary-care-based interventions. |
|  | Mixed |  | Intervention combines multiple settings (e.g. inpatient and outpatient models). | Select if intervention spans more than one type of setting. |
| **Where is TFA utilised:**  Reviewers should only select options when TFA is explicitly mentioned in that section. Any inferred use should be captured in Additional comments. | Design | Multi-choice | TFA explicitly informs the study’s conceptual framing or aims. | Select “design” if TFA is explicitly mentioned in relation to the study design, aims, or conceptual framework. Not all papers will label these sections, so look instead for clear statements such as “this study was guided by the Theoretical Framework of Acceptability” or “TFA informed the design/aims.” If there is no such statement, but one of the stated aims explicitly refers to using TFA, you can also code this as “design.” |
|  | Data collection |  | TFA used to shape interview questions, survey items, or other data-collection tools. | Select if TFA is explicitly mentioned in the data collection section or method description. This includes statements that questionnaires, interview schedules, or surveys were based on, informed by, or structured around TFA constructs. Avoid inferring where not stated by authors. |
|  | Analysis |  | Data were analysed using TFA. | Select if TFA is used as part of the analytic process, for example to code data, structure a framework matrix, or organise themes. This includes studies where themes are developed inductively and then mapped onto TFA constructs (for example, “themes were generated inductively and subsequently organised using the Theoretical Framework of Acceptability”). In these cases, treat the mapping as part of analysis.  NB: If TFA shapes intellectual organisation of data → Analysis. If TFA only shapes layout or presentation → Reporting. |
|  | Interpretation |  | Findings interpreted or discussed in relation to TFA. | Select if TFA is only used when making sense of or discussing the findings, rather than shaping coding or theme development. Look for wording such as “we interpreted our findings in light of the Theoretical Framework of Acceptability” or “the themes were then discussed using TFA constructs,” with no description of TFA being used during coding or framework development.  **Illustrative example from an included study:** Browne et al. (2025) was not coded as interpretation because, although the Results were structured around TFA constructs, the Discussion did not explicitly interpret the findings through the TFA. |
|  | Reporting |  | Results or structure of findings presented according to TFA (e.g. results organised by constructs). | Select if TFA is explicitly visible or named in how findings are presented or structured within the results. This includes results, tables, figures, or subheadings organised by TFA constructs, or statements like “results are presented according to the seven TFA constructs.”  **NB:** If TFA shapes intellectual organisation of data → Analysis. If TFA only shapes layout or presentation → Reporting.  **Illustrative example from an included study:** Browne et al. (2025) presented findings under headings corresponding to selected TFA constructs. This was coded as reporting because the framework structured the presentation of findings. It was not coded as interpretation because the Discussion did not explicitly use the TFA to contextualise or make further sense of the findings. |
|  | Additional comments | Free text | Any additional observations or inferred uses of TFA. | Use this field if you are unsure about any of the above categories or want to flag something that feels relevant but is not clearly reported. For example, you might note that the authors state the study was informed by the TFA, but its use is only clearly described at the interpretation stage. |
| **TFA constructs applied**  Reviewers should tick constructs only when explicitly named or clearly operationalised in the paper. Do not infer conceptual overlap. | Affective attitude | Multi-choice | Emotional or affective response to the intervention (e.g. enjoyment, liking, or aversion). | Must be named or described as *affective attitude* or equivalent wording clearly linked to TFA. |
|  | Burden |  | Effort required to participate in the intervention. | Must be framed as *burden* or equivalent wording clearly linked to TFA. |
|  | Ethicality |  | Degree to which the intervention aligns with the participant’s values. | Must be labelled or described as *ethicality* or similar, with reference to TFA. |
|  | Coherence |  | Understanding of how the intervention works and its purpose. | Must be identified as *coherence* or equivalent within the TFA framework. |
|  | Opportunity costs |  | What participants give up to engage in the intervention (e.g. time, effort, competing demands). | Must be named or described as *opportunity costs* in relation to TFA. |
|  | Perceived effectiveness |  | Perception that the intervention achieves its intended purpose. | Must be labelled *perceived effectiveness* or clearly identified as such within TFA context. |
|  | Self-efficacy |  | Confidence in performing behaviours required by the intervention. | Must be labelled *self-efficacy* or clearly linked to that TFA construct. |
|  | Overall acceptability |  | Overall perception of the intervention’s acceptability. | Must be labelled *overall acceptability* or clearly linked to that TFA construct. |
|  | Other (additional constructs) | Free text | Constructs added beyond the original seven (e.g. “Perceived safety,” “Accessibility”). | Record any adapted or extended constructs explicitly identified by authors. |
|  | Not specified | Multi-choice | Construct-level use not specified or unclear. | Select if authors state using TFA but do not specify individual constructs. |
|  | Additional comments | Free text | Comments on combined or omitted constructs. | May wish to provide detail if constructs were combined or omitted (e.g. “Burden and opportunity cost combined into one theme”). |
| **Positioning of acceptability** | Outcome | Single choice | Acceptability is treated as an endpoint or outcome measure | Use when acceptability is measured as a *result* of the intervention. For example:   - post-intervention survey including an acceptability scale - qualitative study whose stated aim is “to explore the acceptability of X” and treats the themes themselves as the main outcome of interest. |
|  | Process outcome |  | Acceptability is used as a mechanism for understanding or optimising delivery | Use when focus is on whether acceptability is used to *understand delivery* or *implementation*. For example:   - Interim acceptability ratings collected longitudinally and used to decide whether to adjust dose, content, or mode of delivery before the main trial. - Process evaluation interviews with patients and staff exploring barriers, facilitators, and reasons for low or high acceptability, explicitly described as informing refinement or implementation. |
|  | Combined |  | Acceptability is used both as an outcome measure and as a process outcome (e.g. measured quantitatively as an endpoint and explored qualitatively to inform delivery or refinement). | Use when acceptability clearly serves *both roles* in the study. Note briefly in the comments column how this is reflected For example:   - Post-intervention acceptability questionnaire reported as a feasibility outcome, plus nested qualitative interviews used to interpret the scores and refine delivery. |
|  | Additional comments | Free text | Space for any relevant observations about how acceptability is positioned or conceptualised in the study. | Use this field if it is unclear whether acceptability is being treated mainly as an outcome or as process evaluation. Code it in the way that seems most consistent with the authors’ description and use this field to briefly explain your reasoning or note any ambiguity. |
| **Acceptability assessment method/tool** | - | Free text | Method(s) or tools used to assess acceptability (e.g. bespoke questionnaire, TFAQ, TFA-informed interviews). | Use to record how the methods/tools were linked to TFA. For example, Interview guide informed by TFA or Interview data analysed using TFA. Include multiple tools or approaches where relevant. Do no include assessment measures not related to the TFA, |
| **Stakeholder groups included** | Patients/service users only | Single choice | Acceptability assessed only among individuals receiving the intervention. | Code based on whose acceptability is assessed, not who receives the intervention.  Select “multiple stakeholder groups” whenever more than one group is included, regardless of which groups.  If a study includes multiple groups but reports results for only one, code based on the group(s) for whom acceptability data are actually presented.  If stakeholder group is unclear, record in comments. |
|  | Healthcare professionals only; |  | Acceptability assessed only among those delivering or supporting delivery of the intervention (e.g. clinicians, therapists, nurses). |  |
|  | Carers/informal support only; |  | Acceptability assessed only among informal carers, family members, or others supporting patient engagement. |  |
|  | Multiple stakeholder groups |  | Acceptability assessed across two or more stakeholder groups (e.g. patients and healthcare professionals; patients, carers, and professionals). |  |
|  | Other |  | Stakeholder group(s) do not fit the above categories (e.g. policy-makers, service managers, commissioners). |  |
| **Type of TFA data collected** | Quantitative | Single choice | Only quantitative TFA data collected (e.g. survey or scale data). | Select when TFA data are measured numerically. |
|  | Qualitative |  | Only qualitative TFA data collected (e.g. interviews, focus groups, open-ended responses). | Select when TFA data are explored through narrative or thematic data. |
|  | Mixed methods |  | Both qualitative and quantitative TFA data collected. | Select when both data types contribute to TFA analysis. |
|  | Unclear |  | Type of data not clearly reported. | Select when the data source is ambiguous (e.g. registry mentions a “TFA-guided survey” without specifying if data are quantitative, qualitative, or mixed). |
| **Timing of assessment** | - | Free text | When acceptability data were collected (e.g. “baseline and post-intervention,” “immediately after intervention,” “3-month follow-up”). | Provide full details of timing or sequence if available. Use study timepoints or week numbers where stated. |
| **Notes or observations** | - | Free text | Space for any relevant reviewer reflections, contextual notes, or uncertainties not covered elsewhere. | Use for interpretive or methodological notes (e.g. “Acceptability explicitly guided by TFA but operationalisation unclear”; “TFA mentioned in introduction but not applied in analysis”). |

# Additional file 4: Data dictionary for categorisation

| **Variable** | **Category** | **Operational definition** | **Coding rules** |
| --- | --- | --- | --- |
| **Study design:** Code the design represented by the included study entry. For trial registrations and protocols, code the planned parent-study design. For empirical publications, including qualitative or observational substudies, code the design used in the included publication. | Randomised controlled trial | An experimental study in which participants, clusters, sites or other units are allocated to two or more intervention or comparison conditions using a random allocation process. | Code when the publication explicitly reports random allocation, randomisation or a randomised trial design. Include individually randomised, cluster-randomised, crossover and factorial randomised trials. Do not code as an RCT solely because the publication is linked to a randomised parent trial. Qualitative or observational substudies of an RCT should be coded according to the design used in that publication. |
|  | Quasi-experimental (pre-post) | A study that measures outcomes before and after an intervention or change but does not use random allocation. This may involve a single group or a non-randomised comparison group. | Include uncontrolled before-after studies and controlled before-after studies. Code here when the central design involves comparison of outcomes across pre-intervention and post-intervention time points. Do not use for interrupted time-series studies involving multiple observations before and after the intervention; code these as Quasi-experimental (other). |
|  | Quasi-experimental (other) | An intervention evaluation that does not use random allocation and does not fit a simple pre-post design. | Include non-randomised controlled trials, interrupted time-series studies, regression discontinuity designs, natural experiments and other quasi-experimental evaluations. Use when allocation is determined by factors other than randomisation, such as clinical decision, location, service availability or participant preference. Do not use for purely observational studies in which no intervention or system change is being evaluated. |
|  | Cohort study | An observational study that follows a defined group of participants over time to examine outcomes in relation to an exposure, characteristic or experience | Code when participants are grouped according to an exposure, intervention received in routine practice or other characteristic and are followed prospectively or retrospectively to assess outcomes. Include prospective and retrospective cohort studies. Do not code routine pre-post intervention evaluations as cohort studies when the primary purpose is to assess change following an intervention. |
|  | Case-control study | An observational study in which participants are selected according to whether they have a particular outcome or condition, and previous exposures or characteristics are compared between cases and controls. | Code when the sample is explicitly divided into cases and controls based on outcome status. The defining feature is that participants are selected according to the outcome, rather than according to an exposure. Do not use for cohort studies in which exposed and unexposed groups are followed to determine subsequent outcomes. |
|  | Cross-sectional study | An observational study in which exposure, characteristics and outcomes are assessed at a single time point or during a single defined assessment period. | Code when there is no longitudinal follow-up and the primary analysis examines prevalence, associations or group differences using data collected at one time point. Include cross-sectional surveys and one-time assessments. Do not code baseline-only analyses from a longitudinal study as cross-sectional unless the publication explicitly reports a distinct cross-sectional analysis. |
|  | Other quantitative observational study | A quantitative, non-experimental study that does not clearly meet the definition of a cohort, case-control or cross-sectional design. | Use only when the study is observational and quantitative but the specific design cannot be classified more precisely. This may include routinely collected data analyses, ecological studies, descriptive audits or secondary analyses without a clearly identifiable cohort, case-control or cross-sectional structure. Where sufficient information is available, prioritise the more specific observational category. |
|  | Qualitative | A study that primarily uses qualitative data collection and analysis to examine experiences, perspectives, meanings, processes or contextual factors. | Include interviews, focus groups, observations, qualitative diaries and open-ended qualitative data analysed using a recognised qualitative or interpretive approach. Code qualitative process evaluations and qualitative substudies of trials as qualitative studies. Do not code a study as qualitative solely because it contains a small number of open-text survey questions if the primary design and analysis are quantitative. |
| **Intervention type:** Code the primary intervention type; use Multicomponent when no component is clearly primary. | Psychological | An intervention primarily intended to influence psychological processes, emotional wellbeing, cognition, coping or mental health through recognised psychological or psychotherapeutic techniques. | Include counselling, psychotherapy, cognitive behavioural therapy, acceptance-based therapies, mindfulness-based psychological interventions and structured psychological support. Do not code as Psychological when psychological techniques are used only as minor components of a broader self-management or rehabilitation intervention. |
|  | Behavioural / self-management | An intervention primarily intended to change health-related behaviour or increase a person’s ability to manage their condition, symptoms, treatment or daily activities. | Include behaviour-change programmes, health coaching, goal setting, action planning, adherence support, symptom-management education, pacing and structured self-management programmes. Do not use when the main intervention is psychological therapy, physical rehabilitation or another more specific intervention type. |
|  | Speech and language therapy | An intervention primarily focused on communication, speech, language, voice, swallowing or related functional difficulties. | Include interventions delivered or designed within speech and language therapy, such as aphasia rehabilitation, communication training, speech exercises and swallowing therapy. Code here when speech, language, communication or swallowing rehabilitation is the principal intervention focus |
|  | Physical rehabilitation | An intervention primarily focused on improving physical function, mobility, strength, endurance, balance or physical recovery. | Include physiotherapy, exercise therapy, mobility training, balance training, strength and conditioning, pulmonary rehabilitation and other structured physical rehabilitation programmes. Do not code general advice to remain active as Physical rehabilitation unless structured physical training is a central component. |
|  | Digital / monitoring | An intervention primarily delivered through digital technology or focused on the remote or continuous monitoring of symptoms, behaviour or health status. | Include mobile applications, web-based platforms, telehealth systems, wearable devices, remote monitoring, digital prompts and technology-supported feedback. Code here when the technology itself is the principal intervention mechanism. Do not use solely because another intervention is delivered by video call or through an online platform. |
|  | Pharmacological | An intervention involving the administration, modification or evaluation of a medicinal drug or pharmaceutical treatment. | Include prescription medicines, drug dosage changes and comparisons between pharmaceutical treatments. Use when the intervention is explicitly described as pharmacological and does not include a broader biological treatment requiring the Pharmacological / biological category. |
|  | Complementary therapy | A non-mainstream therapeutic intervention used alongside or instead of conventional medical, psychological or rehabilitation treatments. | Include acupuncture, massage, aromatherapy, reflexology and other complementary practices. Do not include recognised psychological therapies, structured exercise or dietary interventions solely because they are described as holistic. |
|  | Multicomponent | An intervention comprising two or more distinct therapeutic components that contribute substantially to the intervention and cannot reasonably be represented by one primary category. | Use when the intervention intentionally combines multiple substantial components, such as exercise, psychological support and self-management education. Do not code as Multicomponent when one intervention type is clearly primary and other elements are minor, supportive or related only to delivery. |
|  | Surgical / procedural | An intervention involving surgery, an invasive procedure or another clinical procedure intended to alter anatomy, physiology or function. | Include surgical operations, catheter-based procedures, device implantation and other invasive clinical procedures. Do not include non-invasive monitoring, medication or routine clinical assessment. |
|  | Occupational therapy | An intervention primarily focused on enabling participation in daily activities, roles, routines and occupations. | Include occupational therapy addressing activities of daily living, functional independence, environmental adaptation, assistive equipment, fatigue management or return to work. Code here when occupational performance is the main intervention focus. |
|  | Pharmacological / biological | An intervention involving a pharmaceutical treatment, biological therapy or another biologically active medical treatment. | Include medications, biologic therapies, immunotherapies, hormones and other treatments acting primarily through biological or physiological mechanisms. Use this category where the source explicitly combines pharmacological and biological interventions or where the intervention extends beyond a conventional drug treatment. |
| **Clinical population:** Code the condition that primarily defines eligibility. | Musculoskeletal / chronic pain | Populations with conditions primarily affecting the musculoskeletal system, physical movement, joints, bones, muscles or persistent pain. | Include arthritis, osteoarthritis, rheumatoid arthritis, back pain, neck pain, fibromyalgia, osteoporosis, musculoskeletal injury and chronic or persistent pain conditions. Code chronic pain here when pain is the defining clinical condition, even where its precise physical cause is unclear. Do not use for pain that is secondary to another clearly defined condition, such as cancer pain, when that underlying condition defines the study population |
|  | Neurological / cognitive | Populations with acquired or progressive conditions affecting the brain, spinal cord, peripheral nervous system, cognition or neurological functioning. | Include stroke, traumatic brain injury, multiple sclerosis, Parkinson’s disease, epilepsy, dementia, acquired cognitive impairment, neuropathy and other neurological disorders. Do not include neurodevelopmental conditions, which should be coded as Neurodevelopmental. Code dementia and progressive cognitive disorders here unless the study primarily defines the population through frailty or general ageing. |
|  | Endocrine / metabolic | Populations with conditions involving hormonal regulation, metabolism or metabolic functioning. | Include diabetes, obesity where treated as a metabolic condition, thyroid disorders, metabolic syndrome and other endocrine or metabolic diseases. Do not code weight-management studies here when the population is not defined by an endocrine or metabolic condition. |
|  | Cancer / oncology | Populations diagnosed with, treated for, recovering from or at elevated risk of malignant disease. | Include all cancer types, cancer survivorship, metastatic or advanced cancer, haematological malignancies and populations undergoing cancer treatment. Code carers or family members here when their inclusion is specifically based on supporting a person with cancer. Do not include benign tumours unless the study explicitly treats them within an oncology population. |
|  | Cardiovascular / vascular | Populations with conditions affecting the heart, circulation or blood vessels. | Include coronary heart disease, heart failure, hypertension, arrhythmias, peripheral vascular disease, venous disease and cardiac rehabilitation populations. Code stroke as Neurological / cognitive, unless the study is explicitly focused on vascular risk or cardiovascular prevention rather than neurological consequences. |
|  | Maternal / reproductive | Populations defined by pregnancy, childbirth, the postnatal period, fertility, reproductive health or gynaecological and reproductive conditions. | Include antenatal and postnatal populations, infertility, menopause, reproductive disorders and pregnancy-related complications. Code reproductive cancers as Cancer / oncology where cancer is the defining condition. |
|  | Geriatric / frailty | Older adult populations defined primarily by frailty, age-related functional decline, vulnerability, falls risk or geriatric care needs. | Include frailty, falls prevention, age-related functional impairment and general geriatric populations. Do not use solely because participants are older adults. Where a specific condition defines the sample, such as stroke, cancer or heart failure, code the relevant condition category instead. |
|  | Mental health | Populations with diagnosed or clinically significant mental health, emotional or psychiatric conditions. | Include depression, anxiety disorders, psychosis, bipolar disorder, eating disorders, post-traumatic stress disorder, substance-related mental health conditions and severe mental illness. Do not code populations experiencing psychological distress secondary to a physical condition as Mental health unless the mental health condition itself defines eligibility or is the primary focus of the study. |
|  | Critical care / acute illness | Populations receiving or recovering from intensive, emergency or acute hospital care, where the acute episode or critical illness defines the population. | Include intensive care survivors, post-intensive care syndrome, acute hospital admissions, sepsis recovery and rehabilitation following critical illness. Where a specific disease primarily defines the sample, code that condition instead unless critical care recovery is the principal focus. |
|  | Multimorbidity / complex conditions | Populations defined by the presence of multiple long-term conditions, interacting health problems or substantial clinical complexity that cannot be represented by one disease category | Use when multimorbidity, multiple chronic conditions or complex care needs are explicit eligibility criteria or central to the research question. Do not use merely because participants may have comorbidities in addition to one primary condition. |
|  | Dermatology / skin | Populations with conditions primarily affecting the skin, hair or nails. | Include eczema, psoriasis, acne, chronic wounds, burns where skin recovery is the focus and other dermatological disorders. Code skin cancer as Cancer / oncology. Code diabetic wounds under Endocrine / metabolic when diabetes defines the population, unless wound care is clearly the primary clinical focus. |
|  | Renal / hepatic | Populations with conditions affecting kidney or liver structure or functioning. | Include chronic kidney disease, renal failure, dialysis populations, liver disease, cirrhosis and hepatic failure. Code transplant populations according to the organ condition where renal or hepatic disease defines the sample |
|  | Respiratory | Populations with conditions primarily affecting breathing, the lungs or respiratory functioning. | Include asthma, chronic obstructive pulmonary disease, bronchiectasis, pulmonary fibrosis, cystic fibrosis and chronic respiratory failure. Code respiratory consequences of an acute critical illness as Critical care / acute illness when recovery from the acute episode defines the study. |
|  | Infectious diseases | Populations with acute, chronic or post-acute conditions caused by infectious organisms. | Include HIV, hepatitis when studied primarily as an infectious disease, tuberculosis, COVID-19, long COVID and other viral, bacterial, fungal or parasitic diseases. Code post-infectious populations here where the original infection remains central to the study. |
|  | Urological | Populations with conditions affecting the urinary tract, bladder, continence or male urological functioning. | Include urinary incontinence, overactive bladder, prostate conditions, urinary tract disorders and other urological diseases. Code prostate, bladder or kidney cancer as Cancer / oncology. Code chronic kidney disease as Renal / hepatic. |
|  | Neurodevelopmental | Populations with conditions originating during development and affecting cognition, communication, behaviour, learning or neurological functioning across the life course. | Include autism, attention deficit hyperactivity disorder, intellectual disability, developmental coordination disorder and other neurodevelopmental conditions. Do not use for acquired neurological or cognitive conditions, which should be coded as Neurological / cognitive. |
|  | Ophthalmology / vision | Populations with conditions affecting the eyes, sight or visual functioning. | Include visual impairment, glaucoma, cataracts, macular degeneration, retinal disease and other ophthalmological conditions. Code neurological visual impairment as Neurological / cognitive when the neurological condition defines the population. |
|  | Congenital / structural | Populations with congenital anomalies or structural conditions present from birth or arising from anatomical abnormalities. | Include congenital heart defects, craniofacial conditions, limb differences and other congenital or structural abnormalities. Where a more specific clinical system clearly defines the study, use that category where appropriate. For example, code congenital heart disease as Cardiovascular / vascular when the cardiovascular condition is the primary focus. |
| **Age group:** Assign one category based on the population’s reported age range. | Adult | The study population consists exclusively of adults. | Code when all participants are aged 18 years or older, or when the study explicitly describes the population as adults and does not include participants aged under 18 years. |
|  | Paediatric | The study population consists exclusively of children or young people below the age of 18 years. | Code when all participants are aged under 18 years, or when the study explicitly describes the population as paediatric, children, adolescents or young people and does not include adults. |
|  | Mixed | The study population includes both paediatric and adult participants. | Code when the reported age range crosses the 18-year boundary or when the study explicitly includes both children or young people and adults. Do not use solely because carers or professionals of a different age group were included; code according to the population whose acceptability of the intervention was assessed. |
| **Construct Engagement:** Derive this from the constructs identified during extraction. | Full set of 7 constructs | All seven original TFA constructs explicitly named or operationalised. | Code only when all seven constructs are clearly included. |
|  | Full set + overall acceptability | All seven constructs plus an additional global acceptability item. | Code only when both are explicitly included. |
|  | Selective use of constructs | Only some TFA constructs used. | Code when fewer than seven constructs are clearly applied. |
|  | Selective + overall acceptability | Selected constructs plus a global item. | Use when both conditions are met. |
|  | Additional constructs introduced | Study adds constructs beyond the original TFA. | Code when new domains are clearly described as extensions. |
|  | Constructs not specified | TFA referenced but specific constructs not named. | Use when domain-level detail is absent. |
|  | Unclear | Insufficient information to determine construct use. | Use only when clearly justified. |
| **Timing of acceptability assessment:** Assign one category; use Multiple timepoints when there are three or more assessments. | Pre-intervention | Acceptability assessed only before participants began or were exposed to the intervention. | Code when acceptability was assessed at a single pre-intervention timepoint and no during-intervention, post-intervention or follow-up assessment was reported. This may include anticipated or prospective acceptability. |
|  | During intervention | Acceptability assessed only while the intervention was being delivered or used. | Code when acceptability was assessed at a single timepoint, or during a defined period, before intervention completion, with no pre-intervention or post-intervention assessment reported. |
|  | Post-intervention | Acceptability assessed only after the intervention had been completed | Code when one acceptability assessment was conducted following intervention completion and no pre-intervention, during-intervention or later follow-up assessment was reported. |
|  | During and post-intervention | Acceptability assessed both during intervention delivery and after intervention completion. | Code when acceptability was assessed at exactly two stages: during intervention delivery and post-intervention. Do not use when a pre-intervention or follow-up assessment was also conducted. |
|  | Pre- and post-intervention | Acceptability assessed both before intervention exposure and after intervention completion. | Code when acceptability was assessed at exactly two stages: pre-intervention and post-intervention. Do not use when an additional during-intervention or follow-up assessment was conducted. Standardise entries recorded as Pre + Post t |
|  | Post-intervention and follow-up | Acceptability assessed immediately or shortly after intervention completion and again at a later follow-up timepoint. | Code when acceptability was assessed at exactly two post-exposure stages: post-intervention and one later follow-up. Do not use when a pre-intervention or during-intervention assessment was also reported. |
|  | Multiple timepoints (≥3) | Acceptability assessed at three or more distinct timepoints. | Code whenever three or more acceptability assessments were reported, regardless of whether these occurred before, during or after the intervention. Examples include pre + during + post, pre + post + follow-up, or repeated post-intervention assessments at three or more timepoints. |
|  | Unclear | The timing of acceptability assessment could not be determined from the available information. | Code only when the study reports assessing acceptability but provides insufficient information to determine when assessment occurred. Do not use when timing can reasonably be assigned to one of the specified categories. |
| **TFA-informed data collection tool;** Code the type of data collection tool that was explicitly developed, adapted or structured using TFA constructs | TFAQ, standardised | A previously published TFA questionnaire (e.g. the TFAQ derived from Sekhon et al.). | Code only if authors explicitly state use of a validated TFA questionnaire. Minor contextual wording changes are acceptable. If items are substantially rewritten, expanded, reduced, or restructured, code as adapted/self-developed. |
|  | TFA-based questionnaire, adapted or self-developed | A questionnaire created or substantially modified by the study team using TFA domains to generate items. | Code when authors describe developing, adapting, or mapping questionnaire items to TFA constructs. The tool must be prospectively informed by TFA. |
|  | TFA-informed interviews | A structured or semi-structured interview guide explicitly designed around TFA domains. | Code only if the Methods section states that interview questions were informed by, structured around, or mapped to TFA constructs. Do not infer from the analysis approach alone. |
|  | TFA-informed focus groups | A focus group discussion guide explicitly developed using TFA constructs. | Code when focus group prompts are stated to reflect TFA domains. If both interviews and focus groups are prospectively TFA-informed, code as Mixed TFA tools. |
|  | TFA-informed workshop or co-design materials | Workshops, stakeholder meetings, or co-design sessions structured using TFA constructs to guide discussion or participatory activities. | Code when TFA domains are explicitly used to structure exercises, prompts, or group discussions. The use must be described prospectively. |
|  | Mixed TFA-informed tools | More than one distinct prospectively TFA-informed data collection method used in the same study (e.g. questionnaire plus interviews; interviews plus focus groups). | Code when at least two separate data collection tools were prospectively informed by TFA. Do not use if TFA was applied only during analysis for one component. |
|  | Other | A TFA-informed data collection approach that does not fit any predefined category. | Specify the format clearly in extraction notes. Use only when clearly justified. |
|  | Unclear | Insufficient methodological detail to determine how TFA informed the data collection tool. | Use only when the Methods lack clarity. Do not assume TFA-informed design unless explicitly stated. |
| **TFA-informed analysis:** Code how TFA was explicitly used in the analysis of acceptability data, rather than the general analytic approach used elsewhere in the study. | Deductive qualitative analysis using TFA constructs | Qualitative data analysed using TFA constructs as a priori coding categories or analytic framework. | Use when authors explicitly state that coding, framework analysis, template analysis, or thematic analysis was structured around TFA domains. Do not use for post hoc interpretive mapping. |
|  | Mixed inductive-deductive qualitative analysis | Qualitative data analysed inductively to generate themes, followed by mapping or organising findings using TFA constructs. | Use when authors describe generating themes inductively and subsequently aligning or mapping them to TFA domains. Both phases must be clearly described. |
|  | Inductive analysis with TFA used interpretively | Qualitative analysis conducted inductively without TFA structuring coding; TFA referenced only in interpretation or discussion. | Use when TFA is applied after themes are developed, primarily to interpret or contextualise findings rather than guide coding. |
|  | No clear analytic integration of TFA | TFA is listed in the paper but the analytic procedure linking data to it is not described. | Use when authors claim TFA use but the analytic pathway is not transparent. |
|  | Descriptive quantitative analysis of TFA constructs | Questionnaire or survey data analysed by reporting TFA construct-level scores (e.g., means, medians, proportions) without inferential modelling beyond basic comparisons. | Code when analysis consists of descriptive statistics or simple comparisons organised by predefined TFA domains. Do not label as deductive. |
|  | Inferential quantitative analysis of TFA constructs | Statistical modelling or hypothesis testing conducted using TFA construct scores (e.g., regression, group comparisons, longitudinal analysis). | Code when authors conduct inferential analyses examining relationships, predictors, or changes in TFA constructs. |
|  | Parallel mixed-methods analysis (qualitative and quantitative analysed separately) | Study includes both qualitative and quantitative TFA-informed data, analysed separately without explicit integration across strands. | Code when both strands use TFA but results are reported independently, with no triangulation, integration, or joint interpretation by construct. |
|  | Integrated mixed-methods TFA analysis | Qualitative and quantitative TFA-informed findings explicitly integrated, triangulated, or synthesised at the level of TFA constructs. | Code when authors describe triangulation, convergence/divergence analysis, joint displays, or integration of strands using TFA domains. |
|  | Unclear | Insufficient methodological detail to determine how TFA informed analysis. | Use only when analytic procedures are not described clearly enough to determine orientation. Do not infer. |

# Additional file 5: Excluded studies

| **#** | **Title** | **Study ID** | **Exclusion reason** |
| --- | --- | --- | --- |
| 1 | Treatment of Hypoxemic Respiratory Failure and ARDS With Protection, Paralysis, and Proning (TheraPPP) Pathway: a Pilot Before and After Study | NCT04070053 | Wrong population |
| 2 | Your Answers When Needing Sleep in New Brunswick (YAWNS NB): A Randomized Controlled Trial Comparing Sleepwell, EMPOWER, and Treatment as Usual for Benzodiazepine Receptor Agonist Discontinuation in Older Adults (Protocol) | NCT04406103 | Wrong population |
| 3 | Identification and Treatment of Hypoxemic Respiratory Failure (HRF) and ARDS With Protection, Paralysis, and Proning: TheraPPP Study | NCT04744298 | Wrong population |
| 4 | From Hardship to Hope: A Peer-led Intervention to Reduce Financial Hardship and Suicide Risk | NCT04840134 | Wrong intervention |
| 5 | Evaluating the Feasibility and Acceptability of a Therapeutically-Grounded Virtual Sister Circle Intervention for Black Women With Depressive Symptoms | NCT04837573 | Wrong intervention |
| 6 | Evaluating the NeoTree: An eHealth Solution to Reduce Neonatal Mortality in Two Low Income Countries: Malawi and Zimbabwe | NCT05127070 | Wrong population |
| 7 | Reducing Health Disparities Through an Adaptive Healthy Eating Program for Underserved Infants in a Home Visiting Program | NCT04977947 | Wrong population |
| 8 | Does Cultivating Self-compassion Improve Resilience to Criticism and Improve Mental Health in Adults With ADHD? | NCT04978792 | Wrong intervention |
| 9 | Peer PrEP Referral + HIV Self-test Delivery for PrEP Initiation Among Young Kenyan Women: Pilot Study & Randomized Trial | NCT04982250 | Wrong intervention |
| 10 | Efficacy and Acceptability of a Monkeypox Curriculum for Disproportionately Impacted Communities | NCT05651581 | Wrong intervention |
| 11 | Feasibility and Acceptability of Home-based Computerised Cognitive Training After Cardiac Surgery | NCT05298540 | Review paper |
| 12 | Differences in Acceptability of Music Therapy Sessions Played Live Compared to a Recording Thereof | NCT06108375 | Wrong intervention |
| 13 | Flexible Attention Sensory Training for Youth with Chronic Pain: a Feasibility and Acceptability Study | NCT06051305 | Wrong intervention |
| 14 | Adaptive Cell Phone Support to Promote Medication Adherence Among Adolescents and Young Adults with Chronic Health Conditions | NCT05719064 | Wrong intervention |
| 15 | The Efficacy of Home-based Strength and Tai-chi Exercise Snacking for Improving Physical Function in Taiwanese and British Older Adults: a Randomised Control Trial | NCT05758727 | Wrong intervention |
| 16 | The Acceptability and Effectiveness of Formal Versus Informal Mindfulness Instruction Among University Students With Self-reported ADHD, Lived Experience of Nonsuicidal Self-injury, or Stress: An Online Randomized Controlled Trial | NCT06038942 | Wrong intervention |
| 17 | Developing a Smartphone Application to Support the Functional Needs of Veterans in Medication Treatment for Opioid Use Disorder | NCT06454903 | Wrong intervention |
| 18 | Development of a Culturally Tailored Resilience-Building Intervention to Facilitate Advance Care Planning Discussions Between Chinese American Patients and Their Family Caregivers | NCT06247215 | TFA not applied |
| 19 | User Testing of the Palliate Intervention, Supporting Lay Carers to Prepare and Administer Anticipatory Medications to Loved Ones at Home at the End of Life | Oak 2024 | Wrong population |
| 20 | Pilot Clinical Trial of Ketamine-assisted Psychotherapy for Methamphetamine Use Disorder and HIV Risk Reduction | NCT06538285 | Wrong intervention |
| 21 | A Piano Training Program to Improve Manual Dexterity and Upper Extremity Function in Subacute Stroke Survivors | NCT06621771 | Wrong intervention |
| 22 | Feasibility of Nurse-performed Gastric Point-of-care Ultrasound to Determine Gastric Content After Gastro-intestinal and Oncologic Surgery: a Pilot Study | NCT06620549 | Wrong population |
| 23 | Dissemination, Acceptability and Adaptation Study of Two Transdiagnostic Psychological Interventions Based on Emotional Regulation (ER) for the Treatment of Alcohol Addiction: Dialectical Behavioral Therapy(DBT) and Unified Protocol (UP) | NCT06366100 | Wrong population |
| 24 | Small Steps for Big Changes: Implementing an Evidence-Based Diabetes Prevention Program Into Diverse Urban Communities | NCT06440395 | TFA not applied |
| 25 | Sound Origins: Assessment of the Acceptability and Accessibility of a Co-designed Music Therapy Intervention | NCT07021469 | Wrong population |
| 26 | A Multi-Site Hybrid Type I Effectiveness-Implementation Randomized Trial of an Emergency Care Action Plan for Infants With Medical Complexity | NCT06993129 | Wrong population |
| 27 | Development of a Personalized, Psychosocial Intervention for Menopausal Individuals With Elevated Dementia Risk | NCT06965686 | Wrong intervention |
| 28 | Adolescent SPEAKS Study: Integrating Supports to Promote PrEP for Black Adolescents Working With Apps | NCT06876324 | TFA not applied |
| 29 | A Universal Primary Care Based Pilot Intervention to Reduce Youth Overdose Risk | NCT07022717 | Wrong intervention |
| 30 | Feasibility, Acceptability, and Preliminary Outcomes of a Mobile App for Coping With Cancer | NCT06923501 | Wrong intervention |
| 31 | Evaluating the Impact of Various Dietary Nitrate Supplementation Forms on Microbiological Markers of Oral Health and Acceptability Among Adult Participants | NCT06956612 | Wrong population |
| 32 | Treatment of Severe Diabetic Macular Oedema With Anti-vascular Endothelial Growth Factor (Anti-VEGF) Monotherapy vs Anti-VEGF Followed by Subthreshold Micropulse Laser When the Thickness of the Central Retina Goes Below 400 Microns: Pragmatic Randomised Equivalence Trial | NCT06985706 | TFA not applied |
| 33 | Acceptability, Fidelity, Safety and Preliminary Efficacy of a Sedentary Behaviour Intervention in Individuals With Spinal Cord Injury: a Pilot Study | NCT06957483 | TFA not applied |
| 34 | Kupewa: Optimizing Strategies to Implement Provider Recommendation of HPV Vaccination for Adolescent Girls and Young Women With HIV in Malawi | NCT07015957 | Wrong intervention |
| 35 | The impact of peer educators on human immunodeficiency viruses (HIV) testing, treatment linkage and viral suppression | CN-02165334 | Wrong intervention |
| 36 | Delivering a higher-protein diet for Trauma Patients (Ignite) | CN-02184479 | TFA not applied |
| 37 | A very brief intervention for physical activity behaviour change in cardiac rehabilitation: the â€˜Measure It!â€™ effectiveness-implementation trial | CN-02460852 | TFA not applied |
| 38 | A randomized control trial to test the effectiveness, feasibility, and acceptability of an education intervention promoting healthy lifestyle to reduce risk factors for metabolic syndrome, among office workers in Ethiopia | CN-02552913 | Wrong intervention |
| 39 | Optimising outcomes for families with children with a Fetal Alcohol Spectrum Disorder diagnosis or 'At Risk' designation: a mixed-methods randomised trial of Parents under Pressure and the AlertÂ® Program | CN-02527239 | TFA not applied |
| 40 | Benefits of education and exercise for knee osteoarthritis | CN-02523362 | TFA not applied |
| 41 | ENgaginG older people Across transitions of care to improve medication manaGEment â€“ Developing and Testing Co-designed Strategies | CN-02734401 | Wrong intervention |
| 42 | Testing a faith-based intervention to quit smoking during Ramadan 2024 | CN-02663796 | Wrong intervention |
| 43 | Kidney-Healthy Eating and Lifestyle Program (Kidney-HELP): randomised Feasibility Study | CN-02658806 | TFA not applied |
| 44 | The Intergenerational Wellbeing and Brain-Healthy Lifestyles Intervention (GenWell-BRAVE): a Feasibility Pilot | CN-02804917 | Wrong population |
| 45 | Active-Prem: exercise Participation in Early Childhood for Children Born Very Preterm | CN-02812534 | Wrong population |
| 46 | Pregnant women's views on how to promote the use of a decision aid for Down syndrome prenatal screening: a theory-informed qualitative study | Agbadje 2018 | TFA not applied |
| 47 | Behaviour and associated determinants of COVID-19 vaccine acceptance and advocacy: a nationwide survey of pharmacy professionals in Qatar | AlHail 2023 | TFA not applied |
| 48 | Effect of low trans-fatty acid intakes on preeclampsia: a randomized controlled trial | Alamolhoda 2020 | TFA not applied |
| 49 | How acceptable is the use of linguistic-phonological intervention in children with cleft palate? A qualitative study in speech therapists | Alighieri 2023 | Wrong population |
| 50 | The retrospective acceptability of high intensity versus low intensity speech intervention in children with a cleft palate: A qualitative study from the parents' point of view using the Theoretical Framework of Acceptability | Alighieri 2023 | Wrong population |
| 51 | Cognitive Training for Emotion-Related Impulsivity and Rumination: protocol for a Pilot Randomized Waitlist-Controlled Trial | Allen 2025 | TFA not applied |
| 52 | "That makes me feel human": a qualitative evaluation of the acceptability of an HIV differentiated care intervention for formerly incarcerated people re-entering community settings in South Africa | An 2022 | Wrong intervention |
| 53 | U-care: youngcan-development of an internet-based self-help program of psychosocial support and psychological treatment | Ander 2013 | Pre-2017 (prior to publication of TFA) |
| 54 | Patient education in the management of coronary heart disease | Anderson 2017 | Review paper |
| 55 | Developing and Evaluating a Parent-child Intervention for Intellectually Able Children With Autism Spectrum Disorders: A Feasibility Study | NCT04574206 | Wrong population |
| 56 | Acceptability and Feasibility of Groups for Specific Versus Mixed-diagnosis Patients of Long-term Conditions | NCT05402358 | TFA not applied |
| 57 | Keep Exercising & Stay Steady (KESS): Development and Feasibility of a Digital Health Intervention to Encourage Exercise Maintenance After Fall Prevention Exercise Programmes End | NCT06447948 | TFA not applied |
| 58 | Exploring the acceptability of a specialist obesity treatment intervention (Fast Track to Health) for adolescents; a mixed method study | 2034761685 | Wrong publication type |
| 59 | Shared decisionâ€making interventions for people with mental health conditions | Aoki 2022 | Review paper |
| 60 | Development of an intervention to increase adherence to nebuliser treatment in adults with cystic fibrosis: cFHealthHub | Arden 2021 | TFA not applied |
| 61 | Do the Memory Support Intervention and Improving Memory for Treatment Facilitate Behavior Change in Cognitive Therapy? | Armstrong 2025 | TFA not applied |
| 62 | Patient acceptability of CITOBOT for cervical cancer screening: A mixed-method study | Arrivillaga 2025 | Wrong intervention |
| 63 | Developing an intervention package to optimise the management of vancomycin therapy using theory informed co-design | Avent 2024 | TFA not applied |
| 64 | Barriers and enabling factors to use of a physical activity behavioural intervention for adults with Type 2 diabetes delivered in primary care: qualitative findings from the Movement as Medicine open pilot study | Avery 2015 | Pre-2017 (prior to publication of TFA) |
| 65 | Guideline-Adherent Physiotherapy for Patients With Hip and Knee Osteoarthritis in Germany: Protocol for an Implementation Research Project Using the Theoretical Domains Framework and the Behavior Change Wheel | Bahns 2023 | TFA not applied |
| 66 | "A strong reason why I enjoy coming to work": Clinician acceptability of a palliative and supportive care intervention (PACT) for older adults with acute myeloid leukemia and their care partners | Bankole 2024 | Wrong population |
| 67 | Development of an implementation plan for the 6-PACK falls prevention programme as part of a randomised controlled trial: protocol for a series of preimplementation studies | Barker 2016 | Pre-2017 (prior to publication of TFA) |
| 68 | Barriers and facilitators to participation in exercise prehabilitation before cancer surgery for older adults with frailty: a qualitative study | Barnes 2023 | TFA not applied |
| 69 | Improving medicines management for people with dementia in primary care: a qualitative study of healthcare professionals to develop a theory-informed intervention | Barry 2020 | TFA not applied |
| 70 | Understanding acceptability in the context of text messages to encourage medication adherence in people with type 2 diabetes | Bartlett 2021 | Wrong intervention |
| 71 | Mindfulness-Based Stress Reduction to Improve Depressive Symptoms and Rheumatoid Arthritis-Related Clinical Outcomes : Results from a Feasibility and Acceptability Trial | Beaulieu 2020 | Wrong publication type |
| 72 | Design and baseline results of a coaching intervention for implementation of trans-radial access in percutaneous coronary intervention | Beaver 2021 | TFA not applied |
| 73 | Treating Suicidal Thoughts and Behaviors Within an Emotional Disorders Framework: acceptability and Feasibility of the Unified Protocol in an Inpatient Setting | Bentley 2017 | TFA not applied |
| 74 | The role of mental health nursing in pediatric hematology/oncology - Part 3: Evaluating feasibility, acceptability, and appreciation | Bernier 2024 | Wrong population |
| 75 | HABIT (Health visitors delivering Advice in Britain on Infant Toothbrushing): a qualitative exploration of the acceptability of a complex oral health intervention | Bhatti 2022 | Wrong population |
| 76 | Development of a targeted client communication intervention for pregnant and post-partum women: a descriptive study | Bogale 2021 | TFA not applied |
| 77 | A Theory and Evidence-Informed e-Cycling Intervention for Individuals Diagnosed With Cancer: development Study | Bourne 2024 | TFA not applied |
| 78 | Improving cognitive function after cardiac surgery: home-based computerised cognitive training (FACCT study) | Bowden 2023 | Wrong intervention |
| 79 | Interventions for improving modifiable risk factor control in the secondary prevention of stroke | Bridgwood 2018 | Review paper |
| 80 | Acceptability and feasibility of continuous glucose monitoring in people with diabetes: protocol for a mixed-methods systematic review of quantitative and qualitative evidence | Brown 2022 | Review paper |
| 81 | 'I wasn't interested until I actually had that consultation': Exploring the acceptability of a community pharmacy-based hypertension visualisation intervention | Brown 2023 | Wrong publication type |
| 82 | Testing a New Prototype Osteoporosis Shared Decision-Making Intervention in Uk Fracture Liaison Services: Patient and Clinician Perspectives of 'Ifrap' | Bullock 2023 | Wrong publication type |
| 83 | Interventions for promoting physical activity in people with chronic obstructive pulmonary disease (COPD) | Burge 2020 | Review paper |
| 84 | Promoting early presentation of breast cancer: development of a psycho-educational intervention | Burgess 2008 | Pre-2017 (prior to publication of TFA) |
| 85 | Women's acceptability of a self-collect HPV same-day screen-and-treat program in a high burden setting in the Pacific | Camara 2022 | Wrong intervention |
| 86 | 3.44 Clinician Perspectives on the Acceptability of Pediatric ASD Therapies: A Mixed-Methods Study | Caven 2022 | Wrong publication type |
| 87 | Parent and Clinician Perspectives on Acceptability of Paediatric Autism Therapies | Caven 2023 | Wrong publication type |
| 88 | Reach Out Emergency Department: partnering With an Economically Disadvantaged Community in the Development of a Text-Messaging Intervention to Address High Blood Pressure | Champoux 2020 | TFA not applied |
| 89 | Prospective Acceptability of Digital Phenotyping Among Pregnant and Parenting People With Opioid Use Disorder | Charron 2024 | Wrong publication type |
| 90 | Barriers and Facilitators to Community Acceptability of Integrating Point-of-Care Testing to Screen for Sickle Cell Disease in Children in Primary Healthcare Settings in Rural Northern Ghana | Chatio 2022 | Wrong publication type |
| 91 | "I don't know much about providing pharmaceutical care to people who are transgender": A qualitative study of experiences and attitudes of pharmacists | Chaudhary 2023 | Wrong population |
| 92 | Evaluation of a theory-informed video intervention to educate underrepresented groups on clinical and genomic research using the Theoretical Framework of Acceptability | Churchill 2023 | Wrong publication type |
| 93 | Parenting Support Intervention During Pregnancy With a Life-Limiting Fetal Condition: A Pilot Study | Cote-Arsenault 2025 | TFA not applied |
| 94 | Implementation of offering choice of pulmonary rehabilitation location to people with COPD: a protocol for the process evaluation of a cluster randomised controlled trial | Cox 2023 | TFA not applied |
| 95 | IMProving care After inherited Cancer Testing (IMPACT) study: protocol of a randomized trial evaluating the efficacy of two interventions designed to improve cancer risk management and family communication of genetic test results | Cragun 2021 | TFA not applied |
| 96 | Development of a theory-informed implementation intervention to improve the triage, treatment and transfer of stroke patients in emergency departments using the Theoretical Domains Framework (TDF): the T<sup>3</sup> Trial | Craig 2017 | TFA not applied |
| 97 | Testing a Program to Help Women with Breast Cancer Stay Strong and Positive | Ctri 2025 | TFA not applied |
| 98 | Acceptability of a complex team-based quality improvement intervention for transient ischemic attack: a mixed-methods study | Damush 2021 | Wrong intervention |
| 99 | The feasibility and acceptability of a supervised exercise programme to improve cardiorespiratory fitness, strength and body composition in women living with obesity: The EXOFFIT study | Davis 2023 | Wrong publication type |
| 100 | Health care providers acceptance of default prescribing of TB preventive treatment for people living with HIV in Malawi: a qualitative study | DeGroot 2024 | Wrong intervention |
| 101 | Parent Education and Counseling (PairEd-C) Intervention to Improve Family-Centered Care: Protocol for a Prospective Acceptability Study Using the Theoretical Framework of Acceptability | Deribe 2024 | Wrong population |
| 102 | Attitudes of COPD patients towards tele-rehabilitation: a cross-sector case study | Dinesen 2013 | Pre-2017 (prior to publication of TFA) |
| 103 | Acceptability of the Kangaroo Mother Care at the University Hospital of Treichville in Cote d'Ivoire | Doukoure 2022 | Wrong intervention |
| 104 | Development of a multi-component lifestyle intervention for preventing type 2 diabetes and cardiovascular risk factors in adults with intellectual disabilities | Dunkley 2018 | TFA not applied |
| 105 | User-centered design of a telehealth-enhanced hybrid cardiac rehabilitation program as hospital quality improvement | Duran 2023 | TFA not applied |
| 106 | Systematic development and feasibility testing of a multibehavioural digital prehabilitation intervention for patients approaching major surgery (iPREPWELL): A study protocol | Durrand 2022 | TFA not applied |
| 107 | Implementing the NICE osteoarthritis guidelines: a mixed methods study and cluster randomised trial of a model osteoarthritis consultation in primary care--the Management of OsteoArthritis In Consultations (MOSAICS) study protocol | Dziedzic 2014 | Pre-2017 (prior to publication of TFA) |
| 108 | Text Messaging Adherence Intervention for Adolescents and Young Adults with Chronic Kidney Disease: Pilot Randomized Controlled Trial and Stakeholder Interviews | Eaton 2020 | TFA not applied |
| 109 | Using patient feedback to adapt intervention materials based on acceptance and commitment therapy for people receiving renal dialysis | Elander 2021 | TFA not applied |
| 110 | Acceptability of the Brushing RemInder 4 Good oral HealTh (BRIGHT) trial intervention: a qualitative study of perspectives of young people and school staff | Elyousfi 2022 | Wrong intervention |
| 111 | Acceptability of a digital return-to-work intervention for common mental disorders: a qualitative study on service user perspectives | Engdahl 2021 | Wrong intervention |
| 112 | Self-Management for Amputee Rehabilitation Using Technology with Support of Peers: a User-Centred Web-Based Program for Lower Limb Loss | Esfandiari 2021 | Wrong publication type |
| 113 | Improving HIV pre-exposure prophylaxis (PrEP) uptake and initiation: process evaluation and recommendation development from a national PrEP program<sup> </sup> | Estcourt 2023 | TFA not applied |
| 114 | Integration of a smart multidose blister package for medication intake: A mixed method ethnographic informed study of older adults with chronic diseases | Faisal 2022 | TFA not applied |
| 115 | Experience with exercise prehabilitation before cancer surgery for older people with frailty | Feng 2019 | Wrong publication type |
| 116 | Patient perspectives on a proposed pharmacy-based colorectal cancer screening program | Ferrari 2023 | TFA not applied |
| 117 | The use of robotic upper limb therapy in routine clinical practice for stroke survivors: Insights from Australian therapists | Flynn 2025 | TFA not applied |
| 118 | Upskilling a workforce to deliver Acceptance and Commitment Therapy to stroke survivors: a qualitative study | Foote 2023 | Wrong publication type |
| 119 | The acceptability of being trained to deliver online, group-based Acceptance and Commitment Therapy to stroke survivors: the experience of third-sector practitioners | Foote 2024 | Wrong population |
| 120 | Very brief intervention for physical activity behaviour change in cardiac rehabilitation: protocol for the 'Measure It!' effectiveness-implementation hybrid trial | Freene 2023 | TFA not applied |
| 121 | The Feasibility of an Online Language Program Delivered Through Music and the Impact of Dosage on Vocabulary Outcomes in Young Children With Down Syndrome | Frizelle 2024 | Wrong intervention |
| 122 | Attitudes of Australian patients undergoing treatment for upper gastrointestinal cancers to different models of nutrition care delivery: Qualitative investigation | Furness 2022 | Wrong publication type |
| 123 | Practice makes perfect: Acceptability and feasibility of a telerehabilitation solution for rehabilitation stroke teams | Gaboury 2018 | Wrong publication type |
| 124 | Acceptability of an enhanced transdiagnostic CBT intervention for adults with anxiety disorders who are parenting an anxious child | Galea 2022 | TFA not applied |
| 125 | Barriers and enablers to influenza vaccination uptake in adults with chronic respiratory conditions: applying the behaviour change wheel to specify multi-levelled tailored intervention content | Gallant 2023 | TFA not applied |
| 126 | Development of an intervention for reducing infant bathing frequency | Goldsmith 2024 | Wrong population |
| 127 | Health Impact of a Mobile-Delivered Diabetes Intervention to Control Blood Pressure in Older Adults | Goodman 2024 | TFA not applied |
| 128 | Stakeholder perspectives of a pilot multicomponent delirium prevention intervention for adult patients with advanced cancer in palliative care units: A behaviour change theory-based qualitative study | Green 2022 | TFA not applied |
| 129 | Upper Airway Control Therapy (U-ACT): The Development of a Non-Pharmacological Intervention for Inducible Laryngeal Obstruction | Haines 2025 | TFA not applied |
| 130 | Applying the COM-B model to creation of an IT-enabled health coaching and resource linkage program for low-income Latina moms with recent gestational diabetes: the STAR MAMA program | Handley 2016 | Pre-2017 (prior to publication of TFA) |
| 131 | Development of a co-designed behaviour change intervention aimed at healthcare professionals recruiting to clinical trials in maternity care | Hanrahan 2022 | Wrong population |
| 132 | OptimalMe Intervention for Healthy Preconception, Pregnancy, and Postpartum Lifestyles: protocol for a Randomized Controlled Implementation Effectiveness Feasibility Trial | Harrison 2022 | TFA not applied |
| 133 | Development of targeted, theory-informed interventions to improve bronchiolitis management | Haskell 2021 | TFA not applied |
| 134 | Acceptability of a physiotherapy-led intensive prone positioning service in intensive care: A qualitative study with multidisciplinary clinicians | Haughton 2025 | Wrong population |
| 135 | The feasibility and acceptability of a physical activity intervention for older people with chronic musculoskeletal pain: The iPOPP pilot trial protocol | Healey 2018 | TFA not applied |
| 136 | A novel naloxone distribution intervention among persons experiencing unsheltered homelessness: acceptability of naloxone training and distribution during an annual point-in-time count | Herrera 2025 | Wrong population |
| 137 | A qualitative exploration of the prospective acceptability of the MiDerm app; a complex digital intervention for adults living with skin conditions | Hewitt 2025 | Wrong intervention |
| 138 | Clinician acceptability of the ReacStep reactive balance training program for fall prevention | Ho 2024 | Wrong population |
| 139 | 'Is Insulin Right for Me?' Development of a theory-informed, web-based resource for reducing psychological barriers to insulin therapy in type 2 diabetes | Holloway 2021 | TFA not applied |
| 140 | Development and feasibility testing of a time-restricted eating intervention for women living with overweight/obesity and HIV in a resource-limited setting of South Africa | Hoosen 2024 | TFA not applied |
| 141 | Patients' perceptions of highly active antiretroviral therapy in relation to treatment uptake and adherence: the utility of the necessity-concerns framework | Horne 2007 | Pre-2017 (prior to publication of TFA) |
| 142 | Can a theory-informed interactive animation increase intentions to engage in physical activity in young people with asthma? | Hoskins 2014 | Pre-2017 (prior to publication of TFA) |
| 143 | Development and pilot testing of a nurse-led common-sense model of self-regulation-based heart failure self-care program | Huang 2025 | TFA not applied |
| 144 | Health Services and Delivery Research | Hughes 2020 | TFA not applied |
| 145 | The Effectiveness of Acceptance and Commitment Therapy on Prolonged Grief Disorder | CN-02743711 | TFA not applied |
| 146 | The effectiveness of a self-efficacy psychoeducational programme to enhance outcomes of patients with end-stage renal disease | CN-01796755 | Pre-2017 (prior to publication of TFA) |
| 147 | Social cognitive therapy in virtual reality for early psychosis | CN-01892317 | TFA not applied |
| 148 | A community-based monitoring programme for the early detection of wet age-related macular degeneration | CN-02775840 | Wrong intervention |
| 149 | Integrating environmental and skill-based approaches to health and resilience: a case study within the European research collaboration RESONATE | CN-02707290 | Wrong intervention |
| 150 | Evaluation of the parenting support group intervention "Everyday life and parenting" for parents of preschool children with neurodevelopmental difficulties | CN-02763698 | TFA not applied |
| 151 | Effect of music therapy in mitigating anxiety and enhancing satisfaction in eldery patients undergoing lower limbs orthopedic surgery under intravertebral anesthesia | CN-02687508 | TFA not applied |
| 152 | Barriers to and facilitators for supporting patient communication in the adult ICU during the COVIDâ€19 pandemic: A qualitative study | Istanboulian 2022 | Wrong intervention |
| 153 | A qualitative pilot study exploring the acceptability of a peer provider delivered substance use brief intervention from the perspective of youth in Kenya | Jaguga 2025 | Wrong population |
| 154 | Bracing Acceptability and Adherence for Knee Osteoarthritis: Findings from a Qualitative Study in the Prop-Oa Multicentre Trial | Jinks 2024 | Wrong publication type |
| 155 | A PROCESS EVALUATION OF THE IMPLEMENTATION OF AN INTERVENTION ('GET SET GO') TO REDUCE POST-STROKE SEDENTARY BEHAVIOUR IN THE RECREATE PILOT CLUSTER RANDOMISED CONTROLLED TRIAL | Johansson 2023 | Wrong publication type |
| 156 | Acceptability of a cessation intervention for pregnant smokers: a qualitative study guided by Normalization Process Theory | Jones 2020 | TFA not applied |
| 157 | "Making an effort for the very elderly": The acceptability of a multidisciplinary intervention to retirement village residents | Jung 2022 | Wrong population |
| 158 | Feasibility and acceptability of persons on long-acting cabotegravir for HIV prevention in the SEARCH Dynamic Choice HIV Prevention trial extension in rural Kenya and Uganda: a longitudinal cohort study | Kakande 2025 | Wrong intervention |
| 159 | Dentists' perspectives on selective caries removal for the management of deep carious lesions in permanent teeth | Kettle 2025 | TFA not applied |
| 160 | Feasibility and acceptability of the real-world implementation of telehealth in motor neuron disease (TiM) service in two specialist MND centers | Knox 2022 | Wrong publication type |
| 161 | Designing strategies to implement a blunt chest injury care bundle using the behaviour change wheel: a multi-site mixed methods study | Kourouche 2019 | TFA not applied |
| 162 | Exploring the determinants of oral health care uptake among women with gestational diabetes mellitus: A qualitative study to develop a logic model and framework for an oral health intervention | Kristensen 2024 | TFA not applied |
| 163 | Linguistic and Cultural Adaptation of a Computer-Based Counseling Program (CARE+ Spanish) to Support HIV Treatment Adherence and Risk Reduction for People Living With HIV/AIDS: a Randomized Controlled Trial | Kurth 2016 | Pre-2017 (prior to publication of TFA) |
| 164 | Healthcare professionals' perceptions of the role of a multidisciplinary approach in improving the care of patients with advanced chronic kidney disease | Kwek 2020 | Wrong intervention |
| 165 | Patient and healthcare provider perceptions of acceptability of fingerstick point-of-care hepatitis C testing at Aboriginal Community Controlled Health Services in Australia | Lafferty 2024 | Wrong intervention |
| 166 | "That was quick, simple, and easy": Patient perceptions of acceptability of point-of-care hepatitis C RNA testing at a reception prison | Lafferty 2022 | Wrong intervention |
| 167 | Client and staff perceptions of acceptability of MyCheck: a direct-to-pathology telehealth and e-testing service for comprehensive bloodborne virus and sexually transmissible infection screening | Lafferty 2024 | Wrong intervention |
| 168 | Understanding factors that could influence patient acceptability of the use of the PINCER intervention in primary care: A qualitative exploration using the Theoretical Framework of Acceptability | Laing 2022 | Wrong intervention |
| 169 | Exploring the acceptability of a risk prediction tool for cardiometabolic risk (gestational diabetes and hypertensive disorders of pregnancy) for use in early pregnancy: A qualitative study | Lang 2025 | Wrong intervention |
| 170 | Acceptability of venue-based HIV testing and prevention interventions for men who have sex with transgender women and transgender women in Lima, Peru: a formative, qualitative study | Lankowski 2024 | Wrong intervention |
| 171 | Preventing pressure ulcers in nursing homes using a care bundle: A feasibility study | Lavallee 2019 | Wrong population |
| 172 | Patient experiences with micronutrient and overall nutrition management after bariatric surgery: identifying facilitators and barriers to implementing care | Lewis 2023 | TFA not applied |
| 173 | Process evaluation of a behaviour change approach to improving clinical practice for detecting hereditary cancer | Long 2019 | Wrong intervention |
| 174 | Optimizing management of low back pain through the pain and disability drivers management model: A feasibility trial | Longtin 2021 | Wrong intervention |
| 175 | A multidimensional approach to assessing intervention fidelity in a process evaluation of audit and feedback interventions to reduce unnecessary blood transfusions: a study protocol | Lorencatto 2016 | Pre-2017 (prior to publication of TFA) |
| 176 | Promoting self-management of breast and nipple pain in breastfeeding women: protocol of a pilot randomized controlled trial | Lucas 2019 | TFA not applied |
| 177 | The impact of a self-management program on volume overload among patients with heart failure in Thailand: a quasi-experimental study | Lungkawong 2025 | TFA not applied |
| 178 | Interventions for the uptake of evidenceâ€based recommendations in acute stroke settings | Lynch 2023 | Review paper |
| 179 | Conventional chest physiotherapy compared to other airway clearance techniques for cystic fibrosis | Main 2023 | Review paper |
| 180 | Radial vs. Femoral Access for Percutaneous Coronary Artery Intervention in Patients With ST-Elevation Myocardial Infarction | Malik 2021 | TFA not applied |
| 181 | Smartphone and tablet self management apps for asthma | MarcanoBelisario 2013 | Pre-2017 (prior to publication of TFA) |
| 182 | Understanding Women's Willingness to Use e-Health for HIV-Related Services: A Novel Application of the Technology Readiness and Acceptance Model to a Highly Stigmatized Medical Condition | Marhefka 2019 | TFA not applied |
| 183 | Identifying intervention strategies to improve HPV vaccine decision-making using behaviour change theory | Marshall 2023 | TFA not applied |
| 184 | Project BETTER: Preliminary Feasibility and Acceptability of a Technology-Delivered Educational Program for Pregnant and Postpartum People with Opioid Use Disorder | Martin 2022 | Wrong intervention |
| 185 | Wearable Activity Technology And Action-Planning (WATAAP) to promote physical activity in cancer survivors: Randomised controlled trial protocol | Maxwell-Smith 2018 | TFA not applied |
| 186 | Long-term care residents' acceptance of a standing intervention: A qualitative intrinsic case study | McCain 2023 | Wrong population |
| 187 | A digital, coach-assisted intervention to address the psychosocial needs of young adult cancer survivors: randomized controlled trial protocol and intervention adaptation process | McCready 2024 | TFA not applied |
| 188 | The Fibromyalgia Active Management and Exercise (FAME) programme: A service evaluation | McIlroy 2022 | Wrong publication type |
| 189 | Views, experience and adherence among pregnant women with gestational diabetes participating in a weight loss study (WELLBABE) | McParlin 2019 | TFA not applied |
| 190 | A pilot study of self-regulation informed goal setting in people with traumatic brain injury | McPherson 2009 | Pre-2017 (prior to publication of TFA) |
| 191 | Designing a behaviour change intervention using COM-B and the Behaviour Change Wheel: co-designing the Healthy Gut Diet for preventing gestational diabetes | Meloncelli 2024 | TFA not applied |
| 192 | Mobile health (mâ€health) smartphone interventions for adolescents and adults with overweight or obesity | Metzendorf 2024 | Review paper |
| 193 | Psychological treatments for people with epilepsy | Michaelis 2020 | Review paper |
| 194 | Telephone followâ€up, initiated by a hospitalâ€based health professional, for postdischarge problems in patients discharged from hospital to home | Mistiaen 2006 | Pre-2017 (prior to publication of TFA) |
| 195 | Personally tailored activities for improving psychosocial outcomes for people with dementia in longâ€term care | MÃ¶hler 2023 | Review paper |
| 196 | Personally tailored activities for improving psychosocial outcomes for people with dementia in community settings | MÃ¶hler 2020 | Review paper |
| 197 | High patient acceptability for a hepatitis C mobile outreach service targeting 'vulnerable' homeless communities - an important component for elimination? | Montague 2020 | Wrong intervention |
| 198 | Improving quality of life for transfemoral amputees: results from a two-year study of the OPRA implant system and rehabilitation protocol | Mortazavi 2025 | TFA not applied |
| 199 | A theory-informed approach to developing visually mediated interventions to change behaviour using an asthma and physical activity intervention exemplar | Murray 2016 | Pre-2017 (prior to publication of TFA) |
| 200 | RESTORE: an exploratory trial of a web-based intervention to enhance self-management of cancer-related fatigue: findings from a qualitative process evaluation | Myall 2015 | Pre-2017 (prior to publication of TFA) |
| 201 | Assessing acceptability of pre-exposure prophylaxis (PrEP) among participants in an HIV vaccine preparedness study in southwestern Uganda | Nakamanya 2022 | Wrong intervention |
| 202 | Treatment Acceptability and Engagement Amongst Patients on Oral and Intravenous Bisphosphonates: A Qualitative Study | Narayanasamy 2022 | Wrong publication type |
| 203 | Development of "Yima Nkqo," a community-based, peer group intervention to support treatment initiation for young adults with HIV in South Africa | Nardell 2023 | TFA not applied |
| 204 | Interventions for fatigue in people with kidney failure requiring dialysis | Natale 2023 | Review paper |
| 205 | Diet and Physical Activity Intervention in CRC Survivors | Nct 2012 | Pre-2017 (prior to publication of TFA) |
| 206 | An Online Intervention to Address Barriers to IBD Medication Adherence | Nct 2012 | Pre-2017 (prior to publication of TFA) |
| 207 | The Effectiveness of Operant Learning Versus Energy Conservation Activity Pacing Treatments in Fibromyalgia | CN-01537317 | Pre-2017 (prior to publication of TFA) |
| 208 | Sober Network IPT for Perinatal Women With Comorbid Substance Use and Depression | CN-01536062 | Pre-2017 (prior to publication of TFA) |
| 209 | MedActive: a Smartphone Intervention to Improve Adherence to Antipsychotic Medications | CN-01490376 | Pre-2017 (prior to publication of TFA) |
| 210 | Effectiveness of a Transdiagnostic Internet-based Treatment for Emotional Disorders | CN-02046893 | Pre-2017 (prior to publication of TFA) |
| 211 | The Breast Cancer Online Rehabilitation Program | CN-01555551 | Pre-2017 (prior to publication of TFA) |
| 212 | Efficacy of D-allulose on Weight and Fat Loss and Insulin Resistance in Non-diabetic Obese Subjects | CN-01560542 | Pre-2017 (prior to publication of TFA) |
| 213 | Internet-based Acceptance and Commitment Therapy for Chronic Pain | CN-01522277 | TFA not applied |
| 214 | Improving Emotion Regulation at the End of Breast Cancer Treatment | CN-01565624 | TFA not applied |
| 215 | Pilot of Pragmatic SBIRT Study | CN-01564785 | Wrong population |
| 216 | Practical Telemedicine to Improve Control and Engagement for Veterans With Clinic-Refractory Diabetes Mellitus | CN-01574271 | TFA not applied |
| 217 | A Physiotherapist Led Intervention to Promote Physical Activity in Rheumatoid Arthritis - a Pilot Study | CN-01662556 | TFA not applied |
| 218 | De-implementation of Low Value Castration for Men With Prostate Cancer | CN-01660919 | Wrong population |
| 219 | Virtual Reality and Outpatient Hysteroscopy | CN-01663938 | TFA not applied |
| 220 | Development and Usability Testing of a Progressive WebApp for Women With Heart Disease | CN-01701952 | Wrong intervention |
| 221 | Evaluation of Effectiveness of Child-oriented Goal-setting in Paediatric Rehabilitation (the ENGAGE Approach) | CN-01984639 | TFA not applied |
| 222 | Brief Acceptance and Commitment Therapy for HIV-infected At-risk Drinkers | CN-01945280 | TFA not applied |
| 223 | A Nurse-led Patient-centred Intervention to Increase Written Advance Directives | CN-01983677 | TFA not applied |
| 224 | Rhythm-based Intervention in Aphasia | CN-02181896 | TFA not applied |
| 225 | Evaluation of the Effectiveness of Child-oriented Goal-setting in Paediatric Rehabilitation (the ENGAGE Approach) | CN-02299621 | TFA not applied |
| 226 | A Web-based Peer Navigation Program for Men With Prostate Cancer | CN-02307849 | TFA not applied |
| 227 | Music Intervention for Preoperative Anxiety and Acute Pain Among Mastectomy Patients | CN-02253358 | TFA not applied |
| 228 | Strength-based Treatment Approach for Adults With ADHD | CN-02253551 | TFA not applied |
| 229 | Implementation of a 12-week Adapted Physical Activity (APA) Program in People Suffering From Chronic Insomnia | CN-02391645 | TFA not applied |
| 230 | Clinical Outcomes With Passive MPKs vs. Powered Prosthetic Knees | CN-02391285 | TFA not applied |
| 231 | Behavioral Activation for Independence in Older Veterans | CN-02431724 | Wrong intervention |
| 232 | Multi-domain Psychoeducation Self-management Program for Adults With MDD in Singapore | CN-02512070 | TFA not applied |
| 233 | Developing a Smartphone-based App to Reduce Self-harm for CAMHS-treated Adolescents | CN-02404936 | Wrong intervention |
| 234 | Digital Intervention to Improve Physical Activity Behaviour In Overweight and Obese Adolescents | CN-02531433 | Wrong intervention |
| 235 | Social Support Intervention Targeting Patients Treated for Cardiac Disease Who Experience Loneliness | CN-02451702 | TFA not applied |
| 236 | Border Region Area Lifestyle Intervention Study for Healthy Neurocognitive Ageing Diabetes | CN-02472497 | TFA not applied |
| 237 | Using Aromatherapy in Substance Use Disorder | CN-02508624 | TFA not applied |
| 238 | Internet-based Mindfulness-based Training (iMBT) for People With Depression | CN-02405824 | Wrong intervention |
| 239 | Mobile Health Intervention to Increase HIV Testing and Linkage to Care | CN-02431536 | Wrong population |
| 240 | Enhanced Medical Rehabilitation for Spinal Cord Injury | CN-02398836 | TFA not applied |
| 241 | Effectiveness of a Novel Exergame-Based Training Concept for Older Adults With Mild Neurocognitive Disorder | CN-02400098 | Wrong intervention |
| 242 | Does Volume-based Enteral Feeding Improve Nutrient Delivery in Hospitalized Critically Ill Children? | CN-02391423 | TFA not applied |
| 243 | Development and Feasibility of an Intervention to Increase Physical Activity Among Young Children | CN-02531191 | Wrong population |
| 244 | The MANTRA Trial (MANdibular TRauma and Antibiotic Use) | CN-02586839 | TFA not applied |
| 245 | Healthy ME: advancing Health Equity in Lymphatic Pain and Lymphedema in Black and Hispanic Women With Breast Cancer | CN-02651713 | Wrong intervention |
| 246 | Metacognitive Training for Negative Symptoms | CN-02626608 | TFA not applied |
| 247 | Interventional Study to Improve Medication Adherence Using Patient Decision Aid | CN-02679694 | Wrong intervention |
| 248 | Assessing the Effects of Robotic Pets on Patients With Dementia in the Acute Care Hospital Setting | CN-02782134 | Wrong intervention |
| 249 | CIFeR - A Clinician-led Intervention to Address Fear of Cancer Recurrence | CN-02739266 | TFA not applied |
| 250 | A Trial for a Preliminary Estimate of the Effectiveness of a Novel Theoretical Framework | CN-02709017 | TFA not applied |
| 251 | Virtual Reality and Post Traumatic Stress | CN-02782137 | TFA not applied |
| 252 | Effects of Emotion-Focused Therapy (EFT) on Mood Awareness and Emotional Reactivity in Bipolar PatientsAwareness and Emotional Reactivity | CN-02791103 | TFA not applied |
| 253 | Pentabiocel in Pediatric IBS | CN-02791060 | TFA not applied |
| 254 | Rol of Verapamil in Radial Artery Spasm During Transradial Angiography | CN-02707579 | TFA not applied |
| 255 | Qnnections: refinement and Pilot Trial of a Suicide Prevention Intervention to Increase Social Connection | CN-02808913 | TFA not applied |
| 256 | Safety and Efficacy Trial of TV5M01 for Radiation-Induced Mucositis in Head and Neck Cancer Patients | CN-02822335 | TFA not applied |
| 257 | Digital Intervention for Psychedelic Preparation (DIPP): comparing Meditation and Music-Based Programs | CN-02811163 | Wrong population |
| 258 | Validation therapy for dementia | Neal 2003 | Pre-2017 (prior to publication of TFA) |
| 259 | "I Wish to Continue Receiving the Reminder Short Messaging Service": A Mixed Methods Study on the Acceptability of Digital Adherence Tools Among Adults Living with HIV on Antiretroviral Treatment in Tanzania | Ngowi 2021 | Wrong intervention |
| 260 | ?I wish to continue receiving the reminder Short Messaging Service?: A mixed methods study on acceptability of digital adherence tools among adults living with HIVon antiretroviral treatment, Tanzania | Ngowi 2021 | Wrong intervention |
| 261 | Acceptability of integration of cervical cancer screening into routine HIV care, associated factors and perceptions among HIV-infected women: a mixed methods study at Mbarara Regional Referral Hospital, Uganda | Ninsiima 2023 | Wrong intervention |
| 262 | A Digital Health Intervention for Stress Relief in Perioperative Care: clinical Study | CN-02719145 | TFA not applied |
| 263 | Dressings and topical agents for treating venous leg ulcers | Norman 2018 | Review paper |
| 264 | Stress and seizures: development and piloting of a self-help stress management intervention for patients with seizures | Novakova 2016 | Pre-2017 (prior to publication of TFA) |
| 265 | An Emergency Department Virtual Observation Unit Fall Prevention Program: A Pilot Acceptability Study | Numata 2025 | Wrong intervention |
| 266 | Applying the COM-B model to designing a tailored physical activity intervention for sedentary African American women with asthma | Nyenhuis 2017 | Wrong publication type |
| 267 | The Acceptability of Using Augmented Reality as a Mechanism to Engage Children in Asthma Inhaler Technique Training: Qualitative Interview Study With Deductive Thematic Analysis | O'Connor 2023 | Wrong intervention |
| 268 | "A Good Day Is Just Being Able to Breathe": Aligning COPD Research with Patient Needs, a Qualitative Study | O'Connor 2025 | Wrong intervention |
| 269 | Study protocol for the implementation and evaluation of a digital-robotic-based intervention for nurses and patients in a hospital: a quantitative and qualitative triangulation based on the Medical Research Council (MRC) framework for developing and evaluating complex interventions | Ohneberg 2022 | TFA not applied |
| 270 | Evaluating the Acceptability of the Drink Less App and the National Health Service Alcohol Advice Web Page: Qualitative Interview Process Evaluation | Oldham 2024 | Wrong intervention |
| 271 | A Novel Web App for Dietary Weight Management: Development, Implementation, and Usability Study | Oliveira 2024 | TFA not applied |
| 272 | Development and psychometric testing of the Acceptability regarding Cognitive Rehabilitation Interventions Survey - Cancer Survivors (ACRIS-CS) | Oliveira 2024 | Wrong publication type |
| 273 | Use of sedative drugs in specialist palliative care (iSedPall): a multi-modal intervention pilot study protocol | Ostgathe 2025 | TFA not applied |
| 274 | Implementing a Goal-Directed Care Bundle after Acute Intracerebral Haemorrhage: process Evaluation for the Third INTEnsive Care Bundle with Blood Pressure Reduction in Acute Cerebral Haemorrhage Trial Study in China | Ouyang 2022 | TFA not applied |
| 275 | Computerâ€based diabetes selfâ€management interventions for adults with type 2 diabetes mellitus | Pal 2013 | Pre-2017 (prior to publication of TFA) |
| 276 | Psychological interventions for people with hemophilia | Palareti 2020 | Review paper |
| 277 | Cultural adaptation of an existing children's weight management programme: the CHANGE intervention and feasibility RCT | Pallan 2019 | TFA not applied |
| 278 | Dietary interventions for adults with chronic kidney disease | Palmer 2017 | Review paper |
| 279 | Psychosocial interventions for men with prostate cancer | Parahoo 2013 | Pre-2017 (prior to publication of TFA) |
| 280 | Designing a Behaviour Change Wheel guided implementation strategy for a hypoxaemic respiratory failure and ARDS care pathway that targets barriers | Parhar 2023 | TFA not applied |
| 281 | Acceptability of Remote Consulting during Covid-19 among Patients with Two Common Longterm Musculoskeletal Conditions: Findings from Three Qualitative Studies and Recommendations for Practice | Paskins 2022 | Wrong publication type |
| 282 | Acceptability of, and preferences for, remote consulting during COVID-19 among older patients with two common long-term musculoskeletal conditions: findings from three qualitative studies and recommendations for practice | Paskins 2022 | Wrong intervention |
| 283 | Empowering With PrEP (E-PrEP), a Peer-Led Social Media-Based Intervention to Facilitate HIV Preexposure Prophylaxis Adoption Among Young Black and Latinx Gay and Bisexual Men: protocol for a Cluster Randomized Controlled Trial | Patel 2018 | TFA not applied |
| 284 | Feasibility pilot study and potential impact of a mobile app intervention led by pharmacist, to support and improve self-management of diabetes, in Cyprus | Pavlidou 2022 | Wrong publication type |
| 285 | IDF2022-0718 Feasibility study of an m-health intervention led by pharmacist aiming to improve self-management of diabetes, in Cyprus | Pavlidou 2023 | Wrong publication type |
| 286 | Development and feasibility study of a community pharmacy intervention to support self-management of patients with type 2 diabetes, in Cyprus...The Royal Pharmaceutical Society Annual Conference, November 10, 2023, London, United Kingdom | Pavlidou 2023 | Wrong publication type |
| 287 | Application of the theoretical framework of acceptability in a surgical setting: Theoretical and methodological insights | Paynter 2023 | Wrong publication type |
| 288 | Usability and Acceptability of Ambulatory Ecg Monitoring in Undiagnosed Syncope: Insights from the Aspired-Q Qualitative Study | Pearsons 2024 | Wrong publication type |
| 289 | Antimicrobial therapy for chronic bacterial prostatitis | Perletti 2013 | Pre-2017 (prior to publication of TFA) |
| 290 | An early intervention for adolescent depression targeting emotional mental images and memory specificity: a process evaluation | Pile 2023 | TFA not applied |
| 291 | Exposure Therapy Acceptability Among Pregnant Latinas With Anxiety: A Qualitative Content Analysis | Ponting 2025 | Wrong intervention |
| 292 | Social network interventions to support cardiac rehabilitation and secondary prevention in the management of people with heart disease | Purcell 2023 | Review paper |
| 293 | An external pilot cluster randomised controlled trial of a theory-based intervention to improve appropriate polypharmacy in older people in primary care (PolyPrime): study protocol | Rankin 2021 | TFA not applied |
| 294 | Protocol for a process evaluation of an external pilot cluster randomised controlled trial of a theory-based intervention to improve appropriate polypharmacy in older people in primary care: the PolyPrime study | Rankin 2021 | Wrong publication type |
| 295 | Telephone interventions for symptom management in adults with cancer | Ream 2020 | Review paper |
| 296 | Adaptation and feasibility of the Copen-scale intervention among childcare workers â€“ a pilot study | 0 | Wrong population |
| 297 | A brief intervention for addressing â€˜food addictionâ€™ introducing the Food Addiction Screening Tool (FAST) | ISRCTN12138612 | Wrong intervention |
| 298 | Evaluation of a clinical service to support the uptake of breast cancer prevention medications by women at increased risk of the disease | ISRCTN15718519 | Wrong intervention |
| 299 | Copen-SCALE: Scaling up an effective intervention to childcare institutions in Copenhagen | ISRCTN14831585 | Wrong population |
| 300 | Development of a Web-Based, Guided Self-help, Acceptance and Commitment Therapy-Based Intervention for Weight Loss Maintenance: Evidence-, Theory-, and Person-Based Approach | Richards 2022 | TFA not applied |
| 301 | Development of an acceptance-based guided self-help behavioural intervention for weight loss maintenance: an evidence-, theory-and person-based approach | Richards 2021 | Wrong publication type |
| 302 | Exercise interventions for cerebral palsy | Ryan 2017 | Review paper |
| 303 | A pragmatic cluster randomised trial evaluating three implementation interventions | Rycroft-Malone 2012 | Pre-2017 (prior to publication of TFA) |
| 304 | Understanding falls and its prevention among adults with visual impairment through behavior change models | Sachidanandam 2024 | TFA not applied |
| 305 | Nutritional therapy for reducing disability and improving activities of daily living in people after stroke | Sakai 2024 | Review paper |
| 306 | Experiences of healthcare and recreation staff with implementing a community exercise program incorporating a healthcare-recreation partnership for people with mobility limitations post-stroke in a pilot randomized trial | Salbach 2019 | Wrong publication type |
| 307 | Acceptability of a novel physical activity and self-management intervention for managing fatigue in rheumatoid arthritis | Salmon 2015 | Pre-2017 (prior to publication of TFA) |
| 308 | Translating a precision dosing approach for opioid use disorder in Puerto Rico: Pilot testing of the clinical utility and patient/provider acceptability | Santiago 2023 | Wrong publication type |
| 309 | Interventions for reducing sedentary behaviour in people with stroke | Saunders 2021 | Review paper |
| 310 | Radial versus femoral access site for percutaneous coronary intervention in patients suffering acute myocardial infarction : a randomized prospective multicenter trial | Schernthaner 2018 | TFA not applied |
| 311 | Psychedelicâ€assisted therapy for treating anxiety, depression, and existential distress in people with lifeâ€threatening diseases | Schipper 2024 | Review paper |
| 312 | Mechanism-based modular psychotherapy versus cognitive behavioural therapy for adolescents and young adults with childhood trauma experiences: study protocol for a feasibility trial within the German Center for Mental Health | Seitz 2025 | TFA not applied |
| 313 | Does prospective acceptability of an intervention influence refusal to participate in a randomised controlled trial? An interview study | Sekhon 2021 | Wrong intervention |
| 314 | Acceptability of therapist training and delivery of a psychological informed gait rehabilitation intervention for people with early rheumatoid arthritis | Sekhon 2022 | Wrong publication type |
| 315 | Therapists acceptability of delivering a psychologically informed gait rehabilitation intervention in early rheumatoid arthritis (GREAT): A qualitative interview study | Sekhon 2020 | Wrong publication type |
| 316 | "It gives you a road map of what to do to solve your problems": acceptability of a combination HIV prevention intervention among adolescent girls in Uganda | SensoyBahar 2023 | Wrong population |
| 317 | Psychological intervention (ConquerFear) for treating fear of cancer recurrence: mediators and moderators of treatment efficacy | Sharpe 2019 | TFA not applied |
| 318 | Augmented reality (AR) as a delivery mechanism for psychological interventions | Sharrad 2022 | Wrong publication type |
| 319 | Planning for Your Advance Care Needs (PLAN): A Communication Intervention to Improve Advance Care Planning among Latino Patients with Advanced Cancer | Shen 2023 | TFA not applied |
| 320 | Behavioural therapies versus other psychological therapies for depression | Shinohara 2013 | Pre-2017 (prior to publication of TFA) |
| 321 | Protocol for a pilot randomized controlled feasibility study of brief interpersonal psychotherapy for addressing social-emotional needs and preventing excess gestational weight gain in adolescents | Shomaker 2020 | TFA not applied |
| 322 | An SMS Text Message-Based Type 2 Diabetes Prevention Program for Hispanic Adolescents With Obesity: Qualitative Co-Design Process | Soltero 2023 | TFA not applied |
| 323 | Feasibility of Fit24, a Digital Diabetes Prevention Program for Hispanic Adolescents: Qualitative Evaluation Study | Soltero 2024 | TFA not applied |
| 324 | Acceptability of a cross-sectoral hospital pharmacist intervention for patients in transition between hospital and general practice: a mixed methods study | Sorensen 2023 | Wrong intervention |
| 325 | eHealth interventions for people with chronic kidney disease | Stevenson 2019 | Review paper |
| 326 | Development of the HT&Me intervention to support women with breast cancer to adhere to adjuvant endocrine therapy and improve quality of life | Stewart 2023 | TFA not applied |
| 327 | Psychological therapies for people with borderline personality disorder | Stoffers-Winterling 2012 | Pre-2017 (prior to publication of TFA) |
| 328 | Perceptions of extended-release buprenorphine among people who received medication for opioid use disorder in jail: a qualitative study | Stopka 2024 | Wrong population |
| 329 | Pushing to keep going, scared: A qualitative study exploring why transcatheter aortic valve implantation to treat aortic stenosis is a highly acceptable intervention to patients and carers | Straiton 2022 | Wrong publication type |
| 330 | Acceptability of the eHealth Intervention Sustainable Worker Digital Support for Persons With Chronic Pain and Their Employers (SWEPPE): Questionnaire and Interview Study | Svanholm 2023 | Wrong intervention |
| 331 | Effectiveness, acceptability, and completeness and quality of intervention reporting of psychological interventions for people with dementia or mild cognitive impairment: Protocol for a mixed-methods systematic review | Svedin 2023 | Review paper |
| 332 | Applying a User-Centered Design Framework to Develop a Remote Research Assessment Protocol for a Randomized Clinical Trial for Toddlers with Early Autism Characteristics | Tagavi 2024 | Wrong intervention |
| 333 | An Evidence-Based Somatic Acupressure Intervention Protocol for Managing the Breast Cancer Fatigue-Sleep Disturbance-Depression Symptom Cluster: development and Validation following the Medical Research Council Framework | Tan 2022 | TFA not applied |
| 334 | Psychological therapies for anxiety and depression in children and adolescents with longâ€term physical conditions | Thabrew 2018 | Review paper |
| 335 | Psychological interventions for multiple sclerosis | Thomas 2006 | Pre-2017 (prior to publication of TFA) |
| 336 | Acceptability of six-monthly PrEP dispensing supported with interim HIV self-testing to simplify PrEP delivery in Kenya: findings from qualitative research | Thuo 2024 | Wrong intervention |
| 337 | Acceptability and adoption of the Face-it health promotion intervention targeting women with prior gestational diabetes and their partners: A qualitative study of the perspectives of healthcare professionals | Timm 2023 | Wrong population |
| 338 | Co-design of an intervention to improve patient participation in discharge medication communication | Tobiano 2024 | Wrong intervention |
| 339 | Physical rehabilitation approaches for the recovery of function and mobility following stroke | Todhunter-Brown 2025 | Review paper |
| 340 | Combining specific task-oriented training with manual therapy to improve balance and mobility in patients after stroke: a mixed methods pilot randomised controlled trial | Traxler 2024 | TFA not applied |
| 341 | Interventions for promoting habitual exercise in people living with and beyond cancer | Turner 2018 | Review paper |
| 342 | Multiâ€disciplinary rehabilitation for acquired brain injury in adults of working age | Turnerâ€Stokes 2015 | Pre-2017 (prior to publication of TFA) |
| 343 | Assessing the feasibility, acceptability, and fidelity of a tele-retinopathy-based intervention to encourage greater attendance to diabetic retinopathy screening in immigrants living with diabetes from China and African-Caribbean countries in Ottawa, Canada: a protocol | Umaefulam 2023 | Wrong intervention |
| 344 | Effect of continuous ingestion of Bifidobacterium and N-acetylglucosamine on VFA reduction. - A randomized, double-blind, placebo-controlled, parallel-group clinical trial | Umin 2016 | Pre-2017 (prior to publication of TFA) |
| 345 | Behavioural activation therapy for depression in adults | Uphoff 2020 | Review paper |
| 346 | Acceptability of self-collected vaginal swabs and point-of-care testing for sexually transmitted and genital infections among pregnant women in Papua New Guinea | Vallely 2024 | Wrong intervention |
| 347 | Introduction of peer support as part of scaling-up integrated care in patients with concomitant diabetes and arterial hypertension at the primary health care level in Slovenia: pilot study protocol...22nd International Conference on Integrated Care, 23-25 May 2022, Odense, Denmark | VirtiÄ 2022 | Wrong publication type |
| 348 | Experiences in the realisation of a research project on anthroposophical medicine in patients with advanced cancer | vonRohr 2000 | Pre-2017 (prior to publication of TFA) |
| 349 | Providing psychological support to people in intensive care: development and feasibility study of a nurse-led intervention to prevent acute stress and long-term morbidity | Wade 2018 | TFA not applied |
| 350 | Preparing for the OptiBreech Trial: A mixed methods implementation and feasibility study | Walker 2022 | Wrong publication type |
| 351 | Effect of a WeChat-Based Hybrid Intervention on the Adaptation Outcomes of People Living With HIV/AIDS: pilot Randomized Controlled Trial | Wang 2025 | TFA not applied |
| 352 | The comparative study on psychological influence of transradial vs transfemoral artery access for percutanous coronary intervention in patients with acute myocardial infarction | Wei 2006 | Pre-2017 (prior to publication of TFA) |
| 353 | A smart pill bottle and text messaging intervention for promoting medication adherence in patients with chronic myeloid leukemia: a pilot study of txt4 TKI | Wen 2025 | TFA not applied |
| 354 | Nonâ€pharmacological interventions for sleep disturbances in people with dementia | Wilfling 2023 | Review paper |
| 355 | Feasibility of a theoretically grounded, multicomponent, physiotherapy intervention aiming to promote autonomous motivation to adopt and maintain physical activity in patients with lower-limb osteoarthritis: protocol for a single-arm trial | Willett 2023 | TFA not applied |
| 356 | Psychological therapies for the management of chronic pain (excluding headache) in adults | Williams 2020 | Review paper |
| 357 | Acceptability of a small changes behavioural weight loss treatment for patients with atrial fibrillation and obesity in cardiac rehabilitation | Williamson 2024 | Wrong publication type |
| 358 | Comparison of Non-thrust Manipulation VS. Muscle Energy Techniques in Management of Patients with Knee Osteoarthritis: a Randomized Clinical Trial | Witwit 2022 | TFA not applied |
| 359 | Cognitive stimulation to improve cognitive functioning in people with dementia | Woods 2023 | Review paper |
| 360 | Development of behaviour change support for young adults within a randomised controlled trial | Worthington 2023 | Wrong publication type |
| 361 | Group psychological counseling-based growth mindset intervention to promote active aging behaviors in older people: protocol for a randomized controlled trial | Xiao 2025 | TFA not applied |
| 362 | Acceptability of an Online Support Group to Improve Physical Activity for People with Huntington's Disease: Preliminary Results of a Qualitative Study | Xiaoyi 2024 | Wrong publication type |
| 363 | Assessing the Psychosocial Impact of Expressive Writing on Adults With Spinal Cord Injury: Qualitative Study | Xie 2025 | TFA not applied |
| 364 | Would they accept it? An interview study to identify barriers and facilitators to user acceptance of a prescribing advice service | Yager 2022 | TFA not applied |
| 365 | Transradial Versus Transfemoral Arterial Access in Liver Cancer Embolization: randomized Trial to Assess Patient Satisfaction | Yamada 2018 | TFA not applied |
| 366 | Acceptability and impact of group interpersonal therapy (IPT-G) on Kenyan adolescent mothers living with human immunodeficiency virus (HIV): a qualitative analysis | Yator 2022 | TFA not applied |
| 367 | Factors of communication disorders in ICU machinery ventilates patients based on the theoretical framework of acceptability:a phenomenological study | Yunjing 2024 | Wrong intervention |
| 368 | Feasibility of incorporating the Pocket colposcope into nurse-led cervical cancer screening programs in Western Kenya | Zeufack 2025 | Wrong intervention |
| 369 | Success rate, efficacy and complications of the transradial artery approach to percutaneous coronary intervention for coronary heart disease | Zhang 2017 | TFA not applied |
| 370 | Effect of Patients Tailored Music Intervention in Relieving Chemotherapy Induced Nausea and Vomiting | Zhang 2017 | Wrong publication type |
| 371 | Mobile health assisted self-monitoring is acceptable for supporting weight loss in rural men: a pragmatic randomized controlled feasibility trial | Eisenhauer 2021 | TFA not applied |
| 372 | Using mHealth for HIV/TB Treatment Support in Lesotho: Enhancing Patient-Provider Communication in the START Study | Hirsch-Moverman 2017 | TFA not applied |
| 373 | SmartMom Text Messaging for Prenatal Education: A Qualitative Focus Group Study to Explore Canadian Women's Perceptions | Munro 2017 | TFA not applied |
| 374 | Smoking Cessation Support by Text Message During Pregnancy: A Qualitative Study of Views and Experiences of the MiQuit Intervention | Sloan 2017 | TFA not applied |
| 375 | Women's views and experiences of a mobile phone-based intervention to support post-abortion contraception in Cambodia | Smith 2017 | TFA not applied |
| 376 | Feasibility Randomized Controlled Trial of ImpulsePal: Smartphone App-Based Weight Management Intervention to Reduce Impulsive Eating in Overweight Adults | vanBeurden 2019 | TFA not applied |
| 377 | Breathing retraining for African-American adolescents with asthma: a pilot study of a school-based randomized controlled trial | Bignall, 2015 | TFA not applied |
| 378 | Mindfulness-based Intervention for Female Adolescents with Chronic Pain: A Pilot Randomized Trial | [Chadi, 2016](https://www.ncbi.nlm.nih.gov/pubmed/27924146) | Pre-2017 (prior to publication of TFA) |
| 379 | Mindfulness-Based Stress Reduction and Group Support Decrease Stress in Adolescents with Cardiac Diagnoses: A Randomized Two-Group Study | Freedenberg et al., 2017 | Pre-2017 (prior to publication of TFA) |
| 380 | The VUKA family program: Piloting a family-based psychosocial intervention to promote health and mental health among HIV infected early adolescents in South Africa | Bhana et al. 2014 | Pre-2017 (prior to publication of TFA) |
| 381 | Randomized controlled trial of the acceptability, feasibility, and preliminary effects of a cognitive behavioral skills building intervention in adolescents with chronic daily headaches: a pilot study | Hickman et al 2015 | Pre-2017 (prior to publication of TFA) |
| 382 | Cognitive Behavior Therapy for Pediatric Functional Abdominal Pain: A Randomized Controlled Trial | Van der Veek 2013 | TFA not applied |
| 383 | Effect of relaxation-breathing training on anxiety and asthma signs/symptoms of children with moderate-to-severe asthma: A randomized controlled trial | Chianget al 2009 | TFA not applied |
| 384 | Feasibility Randomized Controlled Trial of ImpulsePal: Smartphone App-Based Weight Management Intervention to Reduce Impulsive Eating in Overweight Adults | Van Beurden 2019 | TFA not applied |
| 385 | Developing a mHealth intervention to promote uptake of HIV testing among African communities in the UK: a qualitative study | Evans (2016) | Pre-2017 (prior to publication of TFA) |
| 386 | Young People’s Views and Experiences of a Mobile Phone Texting Intervention to Promote Safer Sex Behavior | French (2016) | Pre-2017 (prior to publication of TFA) |
| 387 | What's in a message? Delivering sexual health promotion to young people in Australia via text messaging | Gold (2010) | Pre-2017 (prior to publication of TFA) |
| 388 | Designing Audience-Centered Interactive Voice Response Messages to Promote Cancer Screenings Among Low-Income Latinas | Greaney (2014) | Pre-2017 (prior to publication of TFA) |
| 389 | Using mHealth for HIV/TB Treatment Support in Lesotho: Enhancing Patient-Provider Communication in the START Study | Hirsch‐Moverman (2017) | Pre-2017 (prior to publication of TFA) |
| 390 | Mobile health: Connecting managers, service providers and clients in Bombali district, Sierra Leone. Intervention study on mHealth for maternal and newborn health in resource-poor community and health system settings, Sierra Leone. Midline study report. | Jalloh‐Vos (2013) | TFA not applied |
| 391 | Exploring the use of mobile phone technology for the enhancement of the prevention of mother-to-child transmission of HIV program in Nyanza, Kenya: a qualitative study | Jennings (2013) | Pre-2017 (prior to publication of TFA) |
| 392 | Antenatal health promotion via short message service at a Midwife Obstetrics Unit in South Africa: a mixed methods study | Lau (2014) | Pre-2017 (prior to publication of TFA) |
| 393 | Considerations in using text messages to improve adherence to highly active antiretroviral therapy: a qualitative study among clients in Yaoundé, Cameroon | Mbuagbaw (2012) | Pre-2017 (prior to publication of TFA) |
| 394 | A framework for community ownership of a text messaging programme to improve adherence to antiretroviral therapy and client-provider communication: a mixed methods study | Mbuagbaw (2014) | Pre-2017 (prior to publication of TFA) |
| 395 | Short Text Messages to Motivate HIV Testing Among Men Who have Sex with Men: A Qualitative Study in Lima, Peru | Menacho (2013) | Pre-2017 (prior to publication of TFA) |
| 396 | Building capacity to use m-health in maternal, newborn and child health interventions : final technical report (September 1, 2014 - April 30, 2016 | Missal (2016) | TFA not applied |
| 397 | SmartMom Text Messaging for Prenatal Education: A Qualitative Focus Group Study to Explore Canadian Women’s Perceptions | Munro et al (2017) | TFA not applied |
| 398 | Attitudes towards SMS text message smoking cessation support: a qualitative study of pregnant smokers | Naughton (2013) | Pre-2017 (prior to publication of TFA) |
| 399 | Developing Content for a mHealth Intervention to Promote Postpartum Retention in Prevention of Mother-To-Child HIV Transmission Programs and Early Infant Diagnosis of HIV: A Qualitative Study | Odeny (2014) | Pre-2017 (prior to publication of TFA) |
| 400 | Adolescents' Perspectives on the Use of a Text Messaging Service for Preventive Sexual Health Promotion | Perry (2012) | Pre-2017 (prior to publication of TFA) |
| 401 | Short Message Service (SMS)-Based Intervention to Improve Treatment Adherence among HIV-Positive Youth in Uganda: Focus Group Findings | Rana (2015) | Pre-2017 (prior to publication of TFA) |
| 402 | 'The phone reminder is important, but will others get to know about my illness?' Patient perceptions of an mHealth antiretroviral treatment support intervention in the HIVIND trial in South India | Rodrigues (2015) | Pre-2017 (prior to publication of TFA) |
| 403 | Smoking Cessation Support by Text Message During Pregnancy: A Qualitative Study of Views and Experiences of the MiQuit Intervention | Sloan et al 2017 | TFA not applied |
| 404 | A Qualitative Study Investigating the Use of a Mobile Phone Short Message Service Designed to Improve HIV Adherence and Retention in Care in Canada (WelTel BC1) | Smillie (2014) | Pre-2017 (prior to publication of TFA) |
| 405 | Women’s views and experiences of a mobile phone-based intervention to support post-abortion contraception in Cambodia | Smith et al 2017 | TFA not applied |
| 406 | Text messaging for HIV prevention with young Black men: formative research and campaign development | Wright (2011) | Pre-2017 (prior to publication of TFA) |
| 407 | Acceptability and feasibility of mHealth and community-based directly observed antiretroviral therapy to prevent mother-to-child HIV transmission in South African pregnant women under Option B+: an exploratory study | Nachega (2016) | Pre-2017 (prior to publication of TFA) |
| 408 | Increasing breastfeeding initiation and duration in women with a body mass index ≥30 kg/m²: Developing an intervention from theory to acceptability. International Breastfeeding Journal. 2025; 20(1): 29. | Lyons (2025) | Wrong intervention |
| 409 | Feasibility of in-home monitoring for people with glaucoma: the I-TRAC mixed-methods study. | Stewart et al 2024 | Wrong intervention |
| 410 | Acceptability of identification and management of perinatal anxiety: A qualitative interview study with postnatal women. | Meades et al 2024 | Wrong intervention |
| 411 | Analysis of the social acceptability of a humanised childbirth intervention in Senegal: A qualitative study. | Gelinas et al 2023 | Wrong intervention |

# Additional file 6: Linked records

| **Primary record (included study)** | **Linked source(s)** | **Nature of linkage** |
| --- | --- | --- |
| https://clinicaltrials.gov/study/NCT05439252 | Western et al. (2023) | Trial registry linked to published paper reporting the same study |
| https://trialsearch.who.int/Trial2.aspx?TrialID=ACTRN12623000759695 | Kuruppu et al. (2024) | Trial registry linked to published paper reporting the same study |
| https://trialsearch.who.int/Trial2.aspx?TrialID=ISRCTN65177345 | Pinto et al. (2025) | Trial registry linked to published paper reporting the same study |
| https://clinicaltrials.gov/study/NCT05591625 | Bates et al. (2024) | Trial registry linked to published paper reporting the same study |
| https://trialsearch.who.int/Trial2.aspx?TrialID=ACTRN12624000511538 | Duplicate record | Duplicate trial registration for the same underlying study |
| https://clinicaltrials.gov/study/NCT06576557 | Duplicate record | Duplicate trial registration for the same underlying study |
| https://clinicaltrials.gov/study/NCT04447508 | Duplicate record | Duplicate trial registration for the same underlying study |
| Istanboulian L, Rose L, Yunusova Y, Dale CM. Protocol for a mixed method acceptability evaluation of a codesigned bundled COmmunication intervention for use in the adult ICU during the COVID-19 PandEmic: the COPE study. BMJ open. 2021 Sep 1;11(9):e050347. | Istanboulian et al. (2023) | Protocol linked to subsequent full study publication |

# Additional file 7: Detailed study characteristics

| **#** | **Citation** | **Source type** | **Year** | **Country** | **Study design** | **Intervention type** | **Target population** | **Age group** | **Clinical population** | **Setting** | **Intervention summary** |
| --- | --- | --- | --- | --- | --- | --- | --- | --- | --- | --- | --- |
| 1 | NCT03658226 | Trial registration | 2018 | United Kingdom | Quasi-experimental (other) | Psychological | Adults with treatment-resistant chronic low back pain | Adult | Musculoskeletal / chronic pain | Outpatient | Psychodynamic interpersonal therapy, therapist-delivered, 8 weekly sessions |
| 2 | NCT03696589 | Trial registration | 2020 | United States | Quasi-experimental (pre-post) | Behavioural / self-management | Adults with early-stage Parkinson’s disease | Adult | Neurological / cognitive | Mixed | Physical activity coaching with activity tracker feedback, 6 sessions |
| 3 | NCT05269758 | Trial registration | 2022 | Denmark | Quasi-experimental (other) | Speech and language therapy | Older adults with sarcopenic dysphagia | Adult | Neurological / cognitive | Mixed | Task-oriented swallowing resistance training during admission and after discharge, up to 12 weeks |
| 4 | NCT05403658 | Trial registration | 2022 | Canada | Randomised controlled trial | Behavioural / self-management | Children with obesity | Paediatric | Endocrine / metabolic | Outpatient | Family navigation support alongside multidisciplinary obesity management |
| 5 | NCT05642884 | Trial registration | 2022 | United States | Randomised controlled trial | Physical rehabilitation | Older adults with multiple myeloma undergoing stem cell transplantation | Adult | Cancer / oncology | Community | Supervised telehealth prehabilitation with exercise and education, 8 weeks |
| 6 | NCT06207318 | Trial registration | 2023 | United States | Randomised controlled trial | Psychological | Adults undergoing coronary artery bypass graft surgery | Adult | Cardiovascular / vascular | Inpatient | Telehealth acceptance and commitment therapy, 2 sessions with workbook and mindfulness practice |
| 7 | NCT06399731 | Trial registration | 2024 | Netherlands | Randomised controlled trial | Digital / monitoring | Adults with Parkinson’s disease and mild cognitive impairment | Adult | Neurological / cognitive | Community | Repetitive transcranial magnetic stimulation or home-based transcranial direct current stimulation |
| 8 | NCT06326424 | Trial registration | 2024 | United States | Cohort study | Digital / monitoring | Older adults with dementia in the emergency department | Adult | Neurological / cognitive | Inpatient | Wrist biosensor monitoring of physiological and movement data, up to 48 hours |
| 9 | NCT06446089 | Trial registration | 2024 | Canada | Quasi-experimental (pre-post) | Psychological | Pregnant Chinese immigrant women in Canada | Adult | Maternal / reproductive | Community | Mandarin mobile app based on cognitive behavioural and behavioural activation principles |
| 10 | NCT06383338 | Trial registration | 2024 | Australia | Quasi-experimental (pre-post) | Pharmacological | Children and young adults with lymphoma | Mixed | Cancer / oncology | Outpatient | Probe-drug testing to assess changes in drug metabolism |
| 11 | NCT06666218 | Trial registration | 2024 | United States | Randomised controlled trial | Psychological | Women with recurrent ovarian cancer | Adult | Cancer / oncology | Outpatient | Psycho-behavioural coping workshop for recurrent ovarian cancer, 3 sessions |
| 12 | NCT06497582 | Trial registration | 2024 | China | Randomised controlled trial | Complementary therapy | Breast cancer survivors | Adult | Cancer / oncology | Community | Self-acupressure or standing meditation home practice, 6 weeks |
| 13 | NCT06425510 | Trial registration | 2024 | Puerto Rico | Randomised controlled trial | Physical rehabilitation | Older Latino adults with functional impairment | Adult | Geriatric / frailty | Outpatient | Assistive technology education and skills programme with device provision, 6 weeks |
| 14 | NCT06894836 | Trial registration | 2025 | Australia | Quasi-experimental (pre-post) | Psychological | Young people with depression or anxiety; clinicians; service staff | Mixed | Mental health | Outpatient | Virtual reality therapy for depression and anxiety, 4-5 sessions |
| 15 | NCT06884709 | Trial registration | 2025 | United Kingdom | Quasi-experimental (pre-post) | Physical rehabilitation | Adults post-stroke with lower-limb impairment | Adult | Neurological / cognitive | Inpatient | Activity-based mirror therapy added to usual physiotherapy, 2 weeks |
| 16 | ACTRN12621000554864 | Trial registration | 2021 | Australia | Randomised controlled trial | Multicomponent | ICU patients and family members | Adult | Critical care / acute illness | Mixed | Patient- and family-mediated nutrition and exercise education during and after intensive care |
| 17 | ACTRN12621001741875 | Trial registration | 2021 | New Zealand | Randomised controlled trial | Multicomponent | Adults with knee osteoarthritis | Adult | Musculoskeletal / chronic pain | Community | Electroencephalography neurofeedback or mindfulness meditation training, 12 sessions |
| 18 | ACTRN12621001217897 | Trial registration | 2021 | Australia | Randomised controlled trial | Multicomponent | Adults with persistent low back pain | Adult | Musculoskeletal / chronic pain | Outpatient | Group pain self-management education plus supervised exercise, 5 weeks |
| 19 | ACTRN12622001028796 | Trial registration | 2022 | Australia | Randomised controlled trial | Behavioural / self-management | Adults with chronic illness taking regular medication | Adult | Multimorbidity / complex conditions | Inpatient | Medication education using poster, website and question-builder resources before discharge |
| 20 | ACTRN12622001071718 | Trial registration | 2022 | Australia | Randomised controlled trial | Multicomponent | Adults post bone marrow transplant for haematological cancer | Adult | Cancer / oncology | Community | Telehealth supportive care combining exercise, nutrition and behaviour change, 9 weeks |
| 21 | ACTRN12622000839707 | Trial registration | 2022 | Australia | Randomised controlled trial | Physical rehabilitation | Adults with postoperative hypoxaemia after major abdominal surgery | Adult | Critical care / acute illness | Inpatient | Physiotherapy-led postoperative mobilisation and breathing programme, with or without non-invasive ventilation |
| 22 | ACTRN12622000762752 | Trial registration | 2022 | Australia | Randomised controlled trial | Digital / monitoring | Adults with methamphetamine use disorder | Adult | Mental health | Outpatient | Theta-burst brain stimulation over 4 weeks |
| 23 | ACTRN12622001393741 | Trial registration | 2022 | Australia | Randomised controlled trial | Multicomponent | Women with endometriosis-associated pelvic pain | Adult | Musculoskeletal / chronic pain | Community | Pelvic floor physiotherapy plus mindfulness, 8 weeks |
| 24 | ACTRN12622000610730 | Trial registration | 2022 | Australia | Randomised controlled trial | Multicomponent | Adults with depressive symptoms | Adult | Mental health | Community | Videoconference psychosocial training plus vortioxetine treatment, 8 weeks |
| 25 | ACTRN12623000494639 | Trial registration | 2023 | Australia | Randomised controlled trial | Pharmacological | Children undergoing laser procedures | Paediatric | Dermatology / skin | Outpatient | Topical local anaesthetic before laser procedures |
| 26 | ACTRN12623001085662 | Trial registration | 2023 | Australia | Randomised controlled trial | Behavioural / self-management | Adults receiving haemodialysis | Adult | Renal / hepatic | Community | Teach-back self-management education for fluid and salt control in haemodialysis, 4 sessions |
| 27 | ACTRN12623001013651 | Trial registration | 2023 | Australia | Randomised controlled trial | Behavioural / self-management | Older adults | Adult | Geriatric / frailty | Community | Transition care rehabilitation after hospital discharge |
| 28 | ACTRN12624001034527 | Trial registration | 2024 | Australia | Randomised controlled trial | Behavioural / self-management | Children and young people with pancreatic cancer | Adult | Cancer / oncology | Inpatient | Specialist cancer nurse-led telehealth symptom management and care coordination, 12 weeks |
| 29 | ACTRN12624000658516 | Trial registration | 2024 | Australia | Randomised controlled trial | Multicomponent | Older adults from culturally and linguistically diverse communities | Adult | Geriatric / frailty | Outpatient | Tailored falls-prevention exercise programme for culturally diverse older adults |
| 30 | ACTRN12624000946516 | Trial registration | 2024 | Australia | Randomised controlled trial | Speech and language therapy | Children with cerebral palsy | Paediatric | Neurological / cognitive | Community | Dynamic tactile and temporal cueing speech intervention |
| 31 | ACTRN12624000511538 | Trial registration | 2024 | Australia | Randomised controlled trial | Psychological | Children with emotional or behavioural difficulties | Paediatric | Mental health | Community | Brief parent-child emotions intervention, 3 sessions |
| 32 | ACTRN12624000486527 | Trial registration | 2024 | Australia | Randomised controlled trial | Behavioural / self-management | Adults with epilepsy | Adult | Neurological / cognitive | Community | Modified ketogenic diet supported by digital tools, 12 weeks |
| 33 | ACTRN12624001032549 | Trial registration | 2024 | Australia | Randomised controlled trial | Physical rehabilitation | Adults with advanced non-small cell lung cancer | Adult | Cancer / oncology | Outpatient | Co-designed cancer rehabilitation programme, 12 weeks |
| 34 | ACTRN12624000874516 | Trial registration | 2024 | Australia | Randomised controlled trial | Multicomponent | Men with prostate cancer receiving androgen deprivation therapy | Adult | Cancer / oncology | Community | Remotely delivered healthy eating and exercise programme for prostate cancer |
| 35 | ACTRN12624000042549 | Trial registration | 2024 | Australia | Randomised controlled trial | Behavioural / self-management | Young children with cerebral palsy | Paediatric | Cancer / oncology | Community | Early behaviour-change intervention to increase activity in children with cerebral palsy |
| 36 | ACTRN12625000591459 | Trial registration | 2025 | Australia | Randomised controlled trial | Digital / monitoring | People with long-term breathing failure | Adult | Respiratory | Mixed | In-hospital versus home-based adjustment of non-invasive ventilation settings |
| 37 | ACTRN12625000378426 | Trial registration | 2025 | Australia | Randomised controlled trial | Physical rehabilitation | Stroke survivors with upper-limb impairment | Adult | Neurological / cognitive | Mixed | Robotic arm therapy for stroke recovery, 4 weeks |
| 38 | ACTRN12625000410459 | Trial registration | 2025 | Australia | Randomised controlled trial | Psychological | Women with metastatic breast cancer | Adult | Cancer / oncology | Community | Web-based psychological intervention with added therapist guidance |
| 39 | NCT03788122 | Trial registration | 2018 | Belgium | Qualitative study | Surgical / procedural | Pregnant women and partners considering fetal surgery | Adult | Cancer / oncology | Inpatient | Fetal surgery for congenital malformation |
| 40 | NCT04447508 | Trial registration | 2020 | Canada | Randomised controlled trial | Multicomponent | People with chronic low back pain | Adult | Musculoskeletal / chronic pain | Outpatient | Motivational interviewing plus virtual group exercise for chronic low back pain |
| 41 | NCT05107440 | Trial registration | 2021 | Canada | Quasi-experimental (pre-post) | Behavioural / self-management | People with long COVID | Adult | Infectious diseases | Community | Virtual self-management programme for long COVID |
| 42 | NCT05682183 | Trial registration | 2022 | Singapore | Randomised controlled trial | Behavioural / self-management | Adults with major depressive disorder | Adult | Mental health | Community | Multi-domain psychoeducation and self-management programme, 5 sessions |
| 43 | NCT05935020 | Trial registration | 2023 | Denmark | Randomised controlled trial | Physical rehabilitation | Adults after total knee arthroplasty | Adult | Musculoskeletal / chronic pain | Mixed | Referral to municipal rehabilitation after knee replacement |
| 44 | NCT05745038 | Trial registration | 2023 | United States | Quasi-experimental (pre-post) | Behavioural / self-management | Older adults with cancer receiving oral anticancer treatment | Adult | Cancer / oncology | Community | Social network intervention to build support and treatment engagement, 6 sessions |
| 45 | NCT06377709 | Trial registration | 2024 | United Kingdom | Quasi-experimental (pre-post) | Psychological | Adults with burn injuries | Adult | Dermatology / skin | Inpatient | Acceptance and commitment therapy for burn-related appearance concerns |
| 46 | NCT06733103 | Trial registration | 2024 | United Kingdom | Quasi-experimental (pre-post) | Psychological | Pre-frail stroke survivors and informal carers | Adult | Neurological / cognitive | Outpatient | Group cognitive stimulation and psychoeducation for pre-frail stroke survivors |
| 47 | NCT06667739 | Trial registration | 2024 | Canada | Randomised controlled trial | Behavioural / self-management | Adults with dysvascular lower-limb amputation | Adult | Cardiovascular / vascular | Community | Physical activity coaching and technology support after lower-limb amputation |
| 48 | NCT05182632 | Trial registration | 2021 | Canada | Quasi-experimental (pre-post) | Physical rehabilitation | Older women with urinary incontinence | Adult | Urological | Mixed | Online pelvic floor muscle training group programme with home exercise, 12 weeks |
| 49 | DRKS00034413 | Trial registration | 2025 | Germany | Randomised controlled trial | Behavioural / self-management | Adults with multiple sclerosis | Adult | Neurological / cognitive | Outpatient | Online nutrition and behaviour-change programme for multiple sclerosis, with or without counselling |
| 50 | NCT04613076 | Trial registration | 2020 | Chile | Randomised controlled trial | Psychological | Adults attending primary care cardiovascular and depression programmes | Adult | Multimorbidity / complex conditions | Outpatient | Computer-assisted psychoeducation with therapist support and telephone follow-up |
| 51 | NCT06576557 | Trial registration | 2024 | Ireland | Quasi-experimental (pre-post) | Physical rehabilitation | Adults with degenerative knee pain | Adult | Musculoskeletal / chronic pain | Outpatient | Primary care intervention with clinician training, enhanced consultation and early physiotherapy |
| 52 | NCT06638918 | Trial registration | 2024 | Canada | Cohort study | Physical rehabilitation | People undergoing lower-limb amputation | Adult | Cardiovascular / vascular | Mixed | Virtual reality graded motor imagery for phantom limb pain |
| 53 | NCT06225791 | Trial registration | 2023 | Indonesia | Randomised controlled trial | Behavioural / self-management | Pregnant women; postpartum women; healthcare providers; decision-makers | Adult | Maternal / reproductive | Outpatient | Multiple micronutrient supplementation packaging and behaviour-change communication strategies |
| 54 | ISRCTN12202900 | Trial registration | 2023 | United Kingdom | Randomised controlled trial | Psychological | Adults with multiple sclerosis and sexual or intimacy difficulties; clinical staff | Adult | Neurological / cognitive | Outpatient | Manualised psychosexual intervention for multiple sclerosis, mainly self-guided with clinician support |
| 55 | ISRCTN12693443 | Trial registration | 2024 | United Kingdom | Randomised controlled trial | Pharmacological | Adults with diabetic macular oedema | Adult | Endocrine / metabolic | Outpatient | Anti-vascular endothelial growth factor treatment with or without later micropulse laser |
| 56 | ISRCTN23564393 | Trial registration | 2024 | United Kingdom | Randomised controlled trial | Surgical / procedural | Adults undergoing endovascular revascularisation | Adult | Cardiovascular / vascular | Inpatient | Carbon dioxide contrast medium during endovascular intervention to reduce kidney injury risk |
| 57 | ISRCTN16147125 | Trial registration | 2024 | United Kingdom | Randomised controlled trial | Physical rehabilitation | Older adults after hip fracture | Adult | Musculoskeletal / chronic pain | Mixed | Goal-oriented outdoor mobility programme with therapist support and peer video |
| 58 | ISRCTN16030620 | Trial registration | 2024 | Australia | Randomised controlled trial | Behavioural / self-management | Children attending outpatient clinics; service providers | Paediatric | Multimorbidity / complex conditions | Outpatient | Brief outpatient health and wellbeing assessment linked to clinic visits |
| 59 | ISRCTN86825441 | Trial registration | 2024 | Ethiopia | Randomised controlled trial | Psychological | Adults affected by leprosy and depression | Adult | Mental health | Community | Traumatic stress relief programme compared with waitlist |
| 60 | ISRCTN79060696 | Trial registration | 2025 | United Kingdom | Randomised controlled trial | Psychological | Adults with psychosis and agoraphobic avoidance | Adult | Mental health | Mixed | Automated virtual reality therapy for agoraphobic avoidance in psychosis |
| 61 | ISRCTN13813593 | Trial registration | 2025 | United Kingdom | Randomised controlled trial | Behavioural / self-management | ICU survivors with fatigue; healthcare professionals | Adult | Critical care / acute illness | Community | Web-based fatigue self-management resources plus supportive telephone call |
| 62 | ISRCTN32090559 | Trial registration | 2025 | United Kingdom | Randomised controlled trial | Pharmacological | Adults with rheumatoid arthritis | Adult | Musculoskeletal / chronic pain | Outpatient | Oral versus intramuscular glucocorticoids for rheumatoid arthritis |
| 63 | NCT06877273 | Trial registration | 2025 | United Kingdom | Randomised controlled trial | Digital / monitoring | Adults with multiple sclerosis and cognitive difficulties | Adult | Neurological / cognitive | Mixed | Adapted cognitive rehabilitation programme for multiple sclerosis |
| 64 | NCT05840549 | Trial registration | 2023 | United Kingdom | Randomised controlled trial | Surgical / procedural | Men with prostate cancer and lower urinary tract symptoms | Adult | Cancer / oncology | Mixed | UroLift implants versus transurethral prostate resection before radiotherapy |
| 65 | NCT06114732 | Trial registration | 2023 | United Kingdom | Randomised controlled trial | Multicomponent | Adults with peripheral arterial disease and claudication | Adult | Cardiovascular / vascular | Mixed | Remote behavioural support plus electrical stimulation for intermittent claudication |
| 66 | NCT06768918 | Trial registration | 2024 | Denmark | Quasi-experimental (pre-post) | Physical rehabilitation | Adults receiving systemic cancer treatment | Adult | Cancer / oncology | Mixed | Digital cancer rehabilitation solutions during or after systemic treatment |
| 67 | NCT06652828 | Trial registration | 2024 | Germany | Randomised controlled trial | Physical rehabilitation | Older adults at high risk of falls | Adult | Geriatric / frailty | Outpatient | Perturbation treadmill balance training for falls prevention |
| 68 | NCT06983808 | Trial registration | 2025 | Australia | Randomised controlled trial | Multicomponent | Adults awaiting cardiac surgery | Adult | Cardiovascular / vascular | Outpatient | Digital cardiac prehabilitation programme |
| 69 | NCT06143345 | Trial registration | 2023 | United States | Randomised controlled trial | Physical rehabilitation | Adults with prediabetes and overweight or obesity | Adult | Endocrine / metabolic | Outpatient | Supervised high-intensity interval training, 8 weeks |
| 70 | NCT05695170 | Trial registration | 2022 | United States | Randomised controlled trial | Multicomponent | Adults at high risk of type 2 diabetes and partners | Adult | Endocrine / metabolic | Community | Individual or couple-based lifestyle intervention for type 2 diabetes prevention, 12 months |
| 71 | NCT06747650 | Trial registration | 2024 | United States | Quasi-experimental (pre-post) | Psychological | Adolescents with FASD and mental health difficulties; caregivers | Paediatric | Mental health | Community | Dialectical behaviour therapy adapted for adolescents with fetal alcohol spectrum disorder |
| 72 | NCT06001775 | Trial registration | 2023 | United States | Quasi-experimental (pre-post) | Behavioural / self-management | Adults with mild cognitive impairment or dementia; care partners | Adult | Neurological / cognitive | Outpatient | Patient priorities care conversation with patient or care partner, documented in health record |
| 73 | NCT04574206 | Trial registration | 2020 | United Kingdom | Randomised controlled trial | Psychological | Caregiver-child dyads involving children with autism | Paediatric | Neurodevelopmental | Community | Parent training in elaborative reminiscence or present-tense talk, 20 weeks |
| 74 | NCT06345781 | Trial registration | 2024 | United States | Quasi-experimental (pre-post) | Digital / monitoring | Adults with chronic spinal cord injury and neurogenic bowel | Adult | Neurological / cognitive | Community | Abdominal functional electrical stimulation during bowel care at home |
| 75 | NCT06997874 | Trial registration | 2025 | United Kingdom | Randomised controlled trial | Surgical / procedural | Adults with retinal detachment | Adult | Ophthalmology / vision | Outpatient | Combined cataract and retinal surgery versus retinal surgery alone |
| 76 | ISRCTN21541209 | Trial registration | 2019 | United Kingdom | Randomised controlled trial | Surgical / procedural | Adults with chronic venous leg ulcers | Adult | Cardiovascular / vascular | Mixed | Decellularised dermal allograft plus usual compression therapy for venous leg ulcers |
| 77 | ISRCTN10420559 | Trial registration | 2020 | United Kingdom | Randomised controlled trial | Multicomponent | Adults with chronic dizziness | Adult | Neurological / cognitive | Outpatient | Psychologically informed vestibular rehabilitation versus usual vestibular physiotherapy |
| 78 | ISRCTN62558470 | Trial registration | 2023 | United Kingdom | Qualitative study | Behavioural / self-management | Adults prescribed statins; primary care professionals | Adult | Cardiovascular / vascular | Mixed | Digitally delivered tailored intervention to improve statin adherence |
| 79 | ISRCTN17396524 | Trial registration | 2023 | United Kingdom | Quasi-experimental (pre-post) | Multicomponent | Adults with lumbar spinal stenosis and neurogenic claudication | Adult | Musculoskeletal / chronic pain | Outpatient | Behaviour-change rehabilitation before and after lumbar spinal surgery |
| 80 | ISRCTN36893888 | Trial registration | 2023 | United Kingdom | Randomised controlled trial | Behavioural / self-management | Adults with COPD | Adult | Respiratory | Outpatient | Handheld fan-based breathlessness management package, single session |
| 81 | ISRCTN14403521 | Trial registration | 2023 | United Kingdom | Randomised controlled trial | Pharmacological | Adults with newly diagnosed rheumatoid arthritis | Adult | Musculoskeletal / chronic pain | Outpatient | Subcutaneous versus oral methotrexate in treat-to-target rheumatoid arthritis care |
| 82 | ISRCTN69024008 | Trial registration | 2024 | United Kingdom | Quasi-experimental (pre-post) | Digital / monitoring | Older care home residents with type 2 diabetes | Adult | Endocrine / metabolic | Community | Medication review plus continuous glucose monitoring in care homes |
| 83 | ISRCTN13121835 | Trial registration | 2024 | United Kingdom | Randomised controlled trial | Behavioural / self-management | Adults with chronic breathlessness | Adult | Respiratory | Community | Self-guided internet-based breathlessness self-management programme |
| 84 | NCT05605015 | Trial registration | 2022 | United Kingdom | Randomised controlled trial | Behavioural / self-management | Adults with cardiovascular disease not attending cardiac rehabilitation | Adult | Cardiovascular / vascular | Community | Remote physical activity programme with wearable feedback and trainer support |
| 85 | NCT05308238 | Trial registration | 2022 | United States | Randomised controlled trial | Behavioural / self-management | Adults with Parkinson’s disease | Adult | Neurological / cognitive | Community | Telehealth occupational therapy coaching intervention with activity tracker support |
| 86 | NCT05026385 | Trial registration | 2021 | Canada | Randomised controlled trial | Multicomponent | Adults with obesity and knee osteoarthritis | Adult | Musculoskeletal / chronic pain | Community | Personalised nutrition, resistance training and osteoarthritis self-management programme |
| 87 | NCT06954532 | Trial registration | 2025 | United Kingdom | Quasi-experimental (pre-post) | Psychological | Adults with a history of breast cancer | Adult | Cancer / oncology | Mixed | Cardiac interoceptive training using heartbeat biofeedback tasks |
| 88 | NCT05310565 | Trial registration | 2022 | United States | Quasi-experimental (pre-post) | Digital / monitoring | Adults with colon cancer | Adult | Cancer / oncology | Outpatient | Twice-weekly cervical chiropractic treatment, 6 weeks |
| 89 | NCT04671225 | Trial registration | 2020 | Rwanda | Randomised controlled trial | Psychological | Adults with bipolar disorder | Adult | Mental health | Outpatient | Group psychoeducation for bipolar disorder, 8 weeks |
| 90 | NCT06411223 | Trial registration | 2024 | United States | Quasi-experimental (pre-post) | Pharmacological | Women living with HIV and social care needs | Adult | Infectious diseases | Outpatient | Pharmacist-led telehealth support for switching to long-acting injectable HIV treatment |
| 91 | NCT06867757 | Trial registration | 2025 | United States | Cohort study | Digital / monitoring | Adolescents and young adults with chronic musculoskeletal pain | Mixed | Musculoskeletal / chronic pain | Community | Smartwatch-based monitoring with repeated symptom self-reporting, 12 weeks |
| 92 | Bates et al. (2024) | Protocol | 2024 | United Kingdom | Randomised controlled trial | Psychological | ICU survivors with PTSD symptoms | Adult | Critical care / acute illness | Outpatient | Eye movement desensitisation and reprocessing therapy, up to 16 sessions |
| 93 | Capobianco et al. (2024) | Protocol | 2024 | United Kingdom | Randomised controlled trial | Multicomponent | Children and adolescents with heart conditions | Paediatric | Cardiovascular / vascular | Outpatient | Cardiac rehabilitation programme for children and young people with exercise and psychological support |
| 94 | Graco et al. (2025) | Protocol | 2025 | Australia | Randomised controlled trial | Digital / monitoring | People with motor neurone disease; carers; healthcare professionals | Adult | Neurological / cognitive | Community | Polysomnography-assisted initiation of non-invasive ventilation in motor neurone disease |
| 95 | Johansson et al. (2023) | Protocol | 2023 | United Kingdom | Randomised controlled trial | Behavioural / self-management | Stroke survivors; carers; staff | Adult | Neurological / cognitive | Mixed | Intervention to reduce sedentary behaviour after stroke |
| 96 | Malcolm et al. (2025) | Protocol | 2025 | United Kingdom | Randomised controlled trial | Multicomponent | Children and adolescents after brain tumour treatment | Paediatric | Cancer / oncology | Outpatient | Paediatric neurocognitive rehabilitation for fatigue and thinking difficulties after brain tumour |
| 97 | Mouton et al. (2025) | Protocol | 2025 | Belgium | Randomised controlled trial | Speech and language therapy | Children with cleft palate | Paediatric | Congenital / structural | Outpatient | High-intensity versus low-intensity speech intervention for children with cleft palate |
| 98 | Mullenmeister et al. (2024) | Protocol | 2024 | Germany | Randomised controlled trial | Occupational therapy | People with post-COVID syndrome | Mixed | Infectious diseases | Outpatient | Online occupational therapy for post-COVID condition by teletherapy or pre-recorded videos |
| 99 | Swingwood et al. (2023) | Protocol | 2023 | United Kingdom | Randomised controlled trial | Physical rehabilitation | Critically ill adults requiring ventilatory weaning | Mixed | Critical care / acute illness | Inpatient | Mechanical insufflation-exsufflation to support secretion clearance in intensive care |
| 100 | Welford et al. (2025) | Protocol | 2025 | United Kingdom | Quasi-experimental (pre-post) | Multicomponent | Older care home residents with type 2 diabetes; consultees; care home staff | Adult | Endocrine / metabolic | Inpatient | Medication de-intensification supported by continuous glucose monitoring and staff training |
| 101 | Wong et al. (2023) | Protocol | 2023 | United Kingdom | Randomised controlled trial | Surgical / procedural | Men undergoing prostate radiotherapy with urinary symptoms | Adult | Cancer / oncology | Outpatient | UroLift versus transurethral prostate resection before radiotherapy |
| 102 | Arbour et al. (2024) | Published | 2024 | Canada | Quasi-experimental (pre-post) | Psychological | Women with binge eating disorder | Adult | Mental health | Outpatient | Guided self-help for binge eating disorder with weekly dietitian calls |
| 103 | Barnett et al. (2024) | Published | 2024 | Australia | Randomised controlled trial | Multicomponent | Adults with complex chronic conditions | Adult | Renal / hepatic | Outpatient | Self-selected digital health services for diet and exercise support |
| 104 | Beales et al. (2024) | Published | 2024 | Australia | Randomised controlled trial | Physical rehabilitation | Primary care patients at risk of poor musculoskeletal pain outcomes | Adult | Musculoskeletal / chronic pain | Community | Specialist musculoskeletal physiotherapy within a stratified care pathway |
| 105 | Browne et al. (2025) | Published | 2025 | United States | Qualitative study | Occupational therapy | Autistic children | Paediatric | Neurodevelopmental | Community | Occupational therapy using self-regulation concepts in an equine setting |
| 106 | Bull et al. (2025) | Published | 2025 | United States | Quasi-experimental (pre-post) | Psychological | Adults at risk of post-injury PTSD or depression | Adult | Mental health | Outpatient | Brief preventive intervention for post-traumatic stress and depression after injury |
| 107 | Bullock et al. (2024) | Published | 2024 | United Kingdom | Randomised controlled trial | Physical rehabilitation | Physiotherapists delivering knee osteoarthritis care | Adult | Musculoskeletal / chronic pain | Outpatient | Knee bracing with adherence support, advice and exercise instruction |
| 108 | Casmir et al. (2025) | Published | 2025 | Kenya | Randomised controlled trial | Pharmacological | Adolescent girls and young women seeking post-abortal care | Mixed | Maternal / reproductive | Outpatient | Pre-exposure prophylaxis delivery integrated into post-abortal care |
| 109 | Chen et al. (2025) | Published | 2025 | China | Randomised controlled trial | Complementary therapy | Middle-aged and older adults with knee osteoarthritis | Adult | Musculoskeletal / chronic pain | Community | Self-administered acupressure |
| 110 | Chew-Graham et al. (2022) | Published | 2022 | United Kingdom | Quasi-experimental (pre-post) | Psychological | Adults with sub-threshold depression and long-term conditions | Adult | Mental health | Community | Brief behavioural activation support delivered through community pharmacies |
| 111 | Cleland et al. (2025) | Published | 2025 | United Kingdom | Randomised controlled trial | Speech and language therapy | Children with cleft palate | Paediatric | Congenital / structural | Outpatient | Ultrasound visual biofeedback versus articulation therapy for cleft palate speech |
| 112 | Cornwall et al. (2024) | Published | 2024 | United Kingdom | Qualitative study | Behavioural / self-management | Adults prescribed long-term opioids for chronic non-cancer pain | Adult | Musculoskeletal / chronic pain | Community | Pharmacist-led primary care pain review for long-term opioid use |
| 113 | Davis et al. (2024) | Published | 2024 | Ireland | Randomised controlled trial | Physical rehabilitation | Women living with obesity | Adult | Endocrine / metabolic | Community | Aerobic, resistance or combined exercise programmes over 12 weeks |
| 114 | Dedic et al. (2025) | Published | 2025 | Denmark | Randomised controlled trial | Digital / monitoring | Patients with ischaemic heart disease | Adult | Cardiovascular / vascular | Mixed | Patient-reported outcome measures integrated into cardiac rehabilitation care |
| 115 | Deegan et al. (2024) | Published | 2024 | Ireland | Randomised controlled trial | Multicomponent | Adults with chronic pain | Adult | Musculoskeletal / chronic pain | Community | Online mindfulness and exercise programme for chronic pain, 9 weeks |
| 116 | Enoch et al. (2023) | Published | 2023 | United Kingdom | Qualitative study | Pharmacological | People with geographic atrophy | Adult | Ophthalmology / vision | Inpatient | Intravitreal lampalizumab injections for geographic atrophy |
| 117 | Gaboury et al. (2022) | Published | 2022 | Canada | Quasi-experimental (pre-post) | Behavioural / self-management | Stroke survivors | Adult | Neurological / cognitive | Community | Telerehabilitation platform for stroke care |
| 118 | Gerards et al. (2022) | Published | 2022 | Netherlands | Randomised controlled trial | Physical rehabilitation | Older adults with a history of falls | Adult | Neurological / cognitive | Outpatient | Perturbation-based balance training, 3 sessions |
| 119 | Gerbild et al. (2021) | Published | 2021 | Denmark | Qualitative study | Psychological | Men in cardiac rehabilitation | Adult | Cardiovascular / vascular | Community | Communication about sexuality and erectile dysfunction within cardiac rehabilitation |
| 120 | Green et al. (2025) | Published | 2025 | United Kingdom | Randomised controlled trial | Pharmacological | Women with early-stage breast cancer prescribed endocrine therapy | Adult | Cancer / oncology | Outpatient | Behavioural support intervention to improve endocrine therapy adherence |
| 121 | Green et al. (2024) | Published | 2024 | United Kingdom | Randomised controlled trial | Multicomponent | Women with early-stage breast cancer prescribed endocrine therapy | Adult | Cancer / oncology | Outpatient | Optimised behavioural components to support endocrine therapy adherence |
| 122 | Griffey et al. (2025) | Published | 2025 | United States | Cross-sectional study | Pharmacological | Adults with diabetic ketoacidosis | Adult | Endocrine / metabolic | Inpatient | Subcutaneous insulin treatment pathway for diabetic ketoacidosis |
| 123 | Groarke et al. (2021) | Published | 2021 | Ireland | Randomised controlled trial | Multicomponent | Cancer survivors with overweight or obesity | Adult | Cancer / oncology | Outpatient | Intervention to support weight management and health after cancer treatment |
| 124 | Harris et al. (2023) | Published | 2023 | United Kingdom | Randomised controlled trial | Psychological | Adults with non-affective psychosis and suicidal experiences | Adult | Mental health | Mixed | Cognitive behavioural suicide prevention therapy within a randomised trial |
| 125 | Hendry et al. (2022) | Published | 2022 | United Kingdom | Quasi-experimental (pre-post) | Multicomponent | Adults with early rheumatoid arthritis; therapists | Adult | Musculoskeletal / chronic pain | Outpatient | Home-based gait rehabilitation for early rheumatoid arthritis |
| 126 | Heneka et al. (2023) | Published | 2023 | Australia | Qualitative study | Multicomponent | Men with localised prostate cancer; clinicians and stakeholders | Adult | Cancer / oncology | Outpatient | Virtual prostate cancer survivorship support programme, 12 weeks |
| 127 | Herdman et al. (2022) | Published | 2022 | United Kingdom | Randomised controlled trial | Multicomponent | Adults with persistent dizziness | Adult | Neurological / cognitive | Outpatient | Psychologically informed vestibular rehabilitation versus standard vestibular rehabilitation |
| 128 | Herron et al. (2021) | Published | 2021 | United Kingdom | Randomised controlled trial | Multicomponent | Adults with inflammatory rheumatological conditions | Adult | Musculoskeletal / chronic pain | Outpatient | Nurse-led review to identify and manage comorbidities in inflammatory rheumatological conditions |
| 129 | Istanboulian et al. (2023) | Published | 2023 | Canada | Randomised controlled trial | Multicomponent | ICU patients with advanced airway; family members; healthcare professionals | Adult | Critical care / acute illness | Inpatient | Bundled communication support intervention for intensive care patients with advanced airway |
| 130 | Jafar et al. (2024) | Published | 2024 | Singapore | Randomised controlled trial | Multicomponent | Adults with hypertension in rural South Asia | Adult | Cardiovascular / vascular | Community | Multicomponent hypertension care package in rural primary care |
| 131 | Jafar et al. (2023) | Published | 2023 | Singapore | Randomised controlled trial | Multicomponent | Adults with hypertension in Singapore primary care | Adult | Cardiovascular / vascular | Outpatient | Multicomponent hypertension management with nurse support and medication subsidy |
| 132 | Keyworth et al. (2021) | Published | 2021 | United Kingdom | Cross-sectional study | Behavioural / self-management | Adults with a history of self-harm | Adult | Mental health | Community | Volitional help sheet to prevent and reduce self-harm |
| 133 | Keyworth et al. (2022) | Published | 2022 | United Kingdom | Qualitative study | Behavioural / self-management | Adults with a history of self-harm | Adult | Mental health | Community | Volitional help sheet to prevent and reduce self-harm |
| 134 | Keyworth et al. (2023) | Published | 2023 | United Kingdom | Randomised controlled trial | Behavioural / self-management | Adults with a history of self-harm | Adult | Mental health | Community | Refined volitional help sheet to prevent and reduce self-harm |
| 135 | Kiirya et al. (2025) | Published | 2025 | Uganda | Qualitative study | Behavioural / self-management | Young people living with HIV | Mixed | Infectious diseases | Community | WhatsApp peer support group with educational videos for young people living with HIV |
| 136 | Kuruppu et al. (2024) | Published | 2024 | Sri Lanka | Qualitative study | Behavioural / self-management | ICU survivors after mechanical ventilation | Mixed | Critical care / acute illness | Inpatient | Low-technology communication board for mechanically ventilated intensive care patients |
| 137 | Liptrott et al. (2022) | Published | 2022 | United Kingdom | Qualitative study | Behavioural / self-management | Patients with haematological malignancies | Adult | Cancer / oncology | Outpatient | Telephone support model for people with haematological malignancies |
| 138 | Liptrott et al. (2020) | Published | 2020 | Italy | Qualitative study | Psychological | Adults with haematological malignancy receiving telephone support | Adult | Cancer / oncology | Outpatient | Telephone follow-up support after treatment for haematological malignancies |
| 139 | Locke et al. (2020) | Published | 2020 | United Kingdom | Qualitative study | Digital / monitoring | Adults with chronic pain | Adult | Musculoskeletal / chronic pain | Outpatient | Smartphone-based brainwave entrainment with virtual reality headset, 4 weeks |
| 140 | Lopes et al. (2025) | Published | 2025 | Switzerland | Randomised controlled trial | Digital / monitoring | Adults with cancer receiving immune checkpoint inhibitors | Adult | Cancer / oncology | Outpatient | Electronic patient-reported outcomes model with nurse triage and symptom management |
| 141 | Manduchi et al. (2024) | Published | 2024 | Canada | Randomised controlled trial | Speech and language therapy | Adults with head and neck cancer receiving radiotherapy | Adult | Cancer / oncology | Outpatient | Behavioural swallowing intervention during radiotherapy, low- or high-intensity |
| 142 | McIlroy et al. (2022) | Published | 2022 | United Kingdom | Qualitative study | Multicomponent | Adults with fibromyalgia; healthcare practitioners | Adult | Musculoskeletal / chronic pain | Outpatient | Multidisciplinary fibromyalgia management programme combining education, exercise and psychological strategies |
| 143 | Morris et al. (2023) | Published | 2023 | United Kingdom | Quasi-experimental (pre-post) | Multicomponent | Community-dwelling stroke survivors | Adult | Neurological / cognitive | Community | Dyadic walking behaviour-change intervention after stroke, 12 weeks |
| 144 | Murphy et al. (2022) | Published | 2022 | Lebanon | Qualitative study | Physical rehabilitation | Patients with atherosclerotic cardiovascular disease | Adult | Cardiovascular / vascular | Outpatient | Fixed-dose combination therapy for secondary cardiovascular prevention |
| 145 | Nguyen et al. (2024) | Published | 2024 | Australia | Randomised controlled trial | Multicomponent | Adults with pancreatic cancer | Adult | Cancer / oncology | Outpatient | Intensive nutrition support using feeding tubes |
| 146 | Pattison et al. (2022) | Published | 2022 | Australia | Randomised controlled trial | Behavioural / self-management | Autistic children | Paediatric | Neurodevelopmental | Community | Parent-mediated behavioural sleep intervention for autistic children |
| 147 | Pearsons et al. (2025) | Published | 2025 | United Kingdom | Randomised controlled trial | Digital / monitoring | Patients with syncope | Adult | Neurological / cognitive | Outpatient | Extended ambulatory heart rhythm monitoring patch after unexplained syncope |
| 148 | Pedley et al. (2020) | Published | 2020 | United Kingdom | Quasi-experimental (pre-post) | Psychological | Patients with axial spondyloarthritis | Adult | Musculoskeletal / chronic pain | Outpatient | Telephone-based cognitive behavioural therapy for axial spondyloarthritis |
| 149 | Rizzo et al. (2023) | Published | 2023 | Australia | Randomised controlled trial | Multicomponent | People with chronic low back pain | Adult | Musculoskeletal / chronic pain | #N/A | Multimodal chronic low back pain programme combining pain education and graded movement |
| 150 | Roponen et al. (2025) | Published | 2025 | Finland | Randomised controlled trial | Behavioural / self-management | Adults with moderate to severe depression | Adult | Mental health | Mixed | Group nutrition counselling intervention or matched social support programme |
| 151 | Rushton et al. (2020) | Published | 2020 | United Kingdom | Qualitative study | Psychological | Adults receiving telephone-delivered psychological treatment | Adult | Mental health | Community | Telephone-delivered psychological treatment for common mental health problems |
| 152 | Sara et al. (2025) | Published | 2025 | Australia | Randomised controlled trial | Psychological | Men with prostate cancer receiving androgen deprivation therapy | Adult | Cancer / oncology | Community | Telephone-based psychoeducation and survivorship support for men on androgen deprivation therapy |
| 153 | Sharrad et al. (2023) | Published | 2023 | Australia | Qualitative study | Psychological | Young people with asthma and psychological distress; parents/caregivers; health professionals | Paediatric | Respiratory | Outpatient | Mixed reality psychological resources using cognitive behavioural techniques |
| 154 | Shearsmith et al. (2022) | Published | 2022 | United Kingdom | Randomised controlled trial | Psychological | Older adults with multimorbidity | Adult | Multimorbidity / complex conditions | Community | Behavioural activation delivered by telephone or video call |
| 155 | Simelane et al. (2025) | Published | 2025 | South Africa | Randomised controlled trial | Behavioural / self-management | Adults living with HIV and hypertension; healthcare providers | Adult | Infectious diseases | Outpatient | Patient education for integrated HIV and hypertension self-management |
| 156 | Smyth et al. (2024) | Published | 2024 | Ireland | Cross-sectional study | Physical rehabilitation | Patients awaiting or recovering from cancer surgery; family members; healthcare professionals | Adult | Cancer / oncology | Mixed | Exercise prehabilitation before cancer surgery |
| 157 | Sohanpal et al. (2024) | Published | 2024 | United Kingdom | Randomised controlled trial | Psychological | Patients with COPD and anxiety or depression | Adult | Respiratory | Community | Cognitive behavioural intervention for anxiety and depression in chronic obstructive pulmonary disease |
| 158 | Timm et al. (2022) | Published | 2022 | Sweden | Randomised controlled trial | Behavioural / self-management | Adults with type 2 diabetes or high diabetes risk | Adult | Endocrine / metabolic | Community | Telephone-facilitated health coaching for diabetes prevention and management |
| 159 | Tripathee et al. (2025) | Published | 2025 | United Kingdom | Randomised controlled trial | Behavioural / self-management | Adults with long-term indwelling urinary catheters | Adult | Urological | Community | Weekly prophylactic catheter washouts versus standard care |
| 160 | Vergeer-Hagoort et al. (2024) | Published | 2024 | Netherlands | Quasi-experimental (pre-post) | Psychological | Adults with residual depressive symptoms | Adult | Mental health | Outpatient | Blended positive psychotherapy with face-to-face sessions and digital self-help app |
| 161 | Volkmer et al. (2024) | Published | 2024 | United Kingdom | Randomised controlled trial | Physical rehabilitation | Adults with peripheral arterial disease and intermittent claudication | Adult | Cardiovascular / vascular | Mixed | Home-based walking behaviour-change intervention with physiotherapist support |
| 162 | Weemaes et al. (2025) | Published | 2025 | Netherlands | Randomised controlled trial | Behavioural / self-management | Adult cancer survivors post-treatment | Adult | Cancer / oncology | Community | Remote coaching to support physical activity maintenance after cancer rehabilitation |
| 163 | Weinryb et al. (2025) | Published | 2025 | South Africa | Quasi-experimental (pre-post) | Behavioural / self-management | Women seeking first-trimester abortion care | Adult | Maternal / reproductive | Outpatient | WhatsApp chatbot for contraceptive counselling |
| 164 | Western et al. (2023) | Published | 2023 | United Kingdom | Quasi-experimental (pre-post) | Physical rehabilitation | Older adults with mild cognitive impairment | Adult | Neurological / cognitive | Outpatient | Home-based exercise snacking programme twice daily for 28 days |
| 165 | Deja et al. (2021) | Published | 2021 | United Kingdom | Randomised controlled trial | Pharmacological / biological | Critically ill children with fever and infection | Mixed | Critical care / acute illness | Inpatient | A clinical protocol comparing permissive versus restrictive temperature thresholds for administering antipyretics in critically ill children |

# Additional file 8: Detailed study-level data relating to the application of the TFA

| **Study** | **Citation** | **Setting / population** | **Data type** | **Timing of acceptability assessment** | **TFA utilisation stage** | **Role of TFA in study** | **Acceptability assessment method** | **TFA constructs assessed** | **Construct coverage** |
| --- | --- | --- | --- | --- | --- | --- | --- | --- | --- |
| 1 | NCT03658226 | Outpatient | Quantitative | During intervention | Data collection | TFA-informed questionnaire, adapted or self-developed | Questionnaire | Not specified | Not specified |
| 2 | NCT03696589 | Mixed | Quantitative | Post-intervention | Data collection | TFA-informed questionnaire, adapted or self-developed | Questionnaire | Not specified | Not specified |
| 3 | NCT05269758 | Mixed | Qualitative | Unclear | Data collection | TFA-informed interview topic guide | Interviews | Not specified | Not specified |
| 4 | NCT05403658 | Outpatient (Patients; Carers) | Qualitative | Unclear | Data collection, Interpretation | TFA-informed interview topic guide | Interviews | Not specified | Not specified |
| 5 | NCT05642884 | Community | Mixed methods (Interviews; TFAQ ) | Post-intervention | Data collection | Mixed TFA-informed tools | Mixed | All constructs | Full |
| 6 | NCT06207318 | Inpatient | Quantitative | Post-intervention | Data collection | TFAQ, standardised | Questionnaire | All constructs | Full |
| 7 | NCT06399731 | Community | Mixed methods (Focus group; Questionnaire) | Post-intervention | Data collection | Mixed TFA-informed tools | Mixed | Not specified | Not specified |
| 8 | NCT06326424 | Inpatient (Patients; Carers) | Quantitative | Post-intervention | Data collection | TFA-informed questionnaire, adapted or self-developed | Questionnaire | Not specified | Not specified |
| 9 | NCT06446089 | Community | Unclear | Post-intervention | Data collection | TFA-informed questionnaire, adapted or self-developed | Questionnaire | Not specified | Not specified |
| 10 | NCT06383338 | Outpatient | Quantitative | Unclear | Design, Data collection | TFA-informed questionnaire, adapted or self-developed | Questionnaire | All constructs | Full |
| 11 | NCT06666218 | Outpatient | Quantitative | Post-intervention | Data collection, Analysis | TFAQ, standardised; Descriptive quantitative analysis of TFA constructs | Questionnaire | All constructs | Full |
| 12 | NCT06497582 | Community | Quantitative | Post-intervention | Data collection | TFA-informed questionnaire, adapted or self-developed | Questionnaire | Not specified | Not specified |
| 13 | NCT06425510 | Outpatient | Quantitative | Post-intervention | Data collection | TFA-informed questionnaire, adapted or self-developed | Questionnaire | All constructs | Full |
| 14 | NCT06894836 | Outpatient | Quantitative | Post-intervention | Data collection | TFAQ, standardised | Questionnaire | Not specified | Not specified |
| 15 | NCT06884709 | Inpatient (Patients; HCPs) | Unclear | Post-intervention | Data collection | TFA-informed questionnaire, adapted or self-developed | Questionnaire | Not specified | Not specified |
| 16 | ACTRN12621000554864 | Mixed (Patients; Carers) | Quantitative | Post-intervention | Data collection | TFAQ, standardised | Questionnaire | All constructs | Full |
| 17 | ACTRN12621001741875 | Community | Quantitative | Pre- and post-intervention | Data collection | TFA-informed questionnaire, adapted or self-developed | Questionnaire | All constructs | Full |
| 18 | ACTRN12621001217897 | Outpatient | Qualitative | Post-intervention | Data collection | TFA-informed interview topic guide | Interviews | Not specified | Not specified |
| 19 | ACTRN12622001028796 | Inpatient (Patients; HCPs) | Quantitative | Post-intervention | Data collection | TFAQ, standardised | Questionnaire | Not specified | Not specified |
| 20 | ACTRN12622001071718 | Community (Patients; HCPs) | Unclear | Post-intervention | Data collection | TFA-informed questionnaire, adapted or self-developed | Questionnaire | Not specified | Not specified |
| 21 | ACTRN12622000839707 | Inpatient (Patients; HCPs) | Unclear | Pre- and post-intervention | Data collection | TFA-informed questionnaire, adapted or self-developed | Questionnaire | Not specified | Not specified |
| 22 | ACTRN12622000762752 | Outpatient | Unclear | Post-intervention | Data collection | TFA-informed questionnaire, adapted or self-developed | Questionnaire | Not specified | Not specified |
| 23 | ACTRN12622001393741 | Community | Qualitative | Post-intervention | Data collection | TFA-informed interview topic guide | Interviews | Not specified | Not specified |
| 24 | ACTRN12622000610730 | Community | Qualitative | Post-intervention | Data collection | TFA-informed questionnaire, adapted or self-developed | Questionnaire | Affective attitude; Burden; Opportunity cost; Perceived effectiveness; Self-efficacy | Selective |
| 25 | ACTRN12623000494639 | Outpatient (Patients; Carers; HCPs) | Unclear | Multiple timepoints (≥3) | Data collection | TFA-informed questionnaire, adapted or self-developed | Questionnaire | Not specified | Not specified |
| 26 | ACTRN12623001085662 | Community | Quantitative | Post-intervention | Data collection | TFAQ, standardised | Questionnaire | Not specified | Not specified |
| 27 | ACTRN12623001013651 | Community (Patients; Carers; HCPs) | Mixed methods (Interviews; TFAQ ) | Post-intervention | Data collection | Mixed TFA-informed tools | Mixed | Not specified | Not specified |
| 28 | ACTRN12624001034527 | Inpatient | Unclear | During intervention | Data collection | TFA-informed questionnaire, adapted or self-developed | Questionnaire | Not specified | Not specified |
| 29 | ACTRN12624000658516 | Outpatient (Patients; HCPs) | Mixed methods (Interviews; Questionnaire) | Multiple timepoints (≥3) | Data collection | Mixed TFA-informed tools | Mixed | Not specified | Not specified |
| 30 | ACTRN12624000946516 | Community (Carers; HCPs) | Quantitative | Post-intervention | Data collection | TFAQ, standardised | Questionnaire | Not specified | Not specified |
| 31 | ACTRN12624000511538 | Community | Quantitative | Pre- and post-intervention | Data collection | TFA-informed questionnaire, adapted or self-developed | Questionnaire | Not specified | Not specified |
| 32 | ACTRN12624000486527 | Community | Quantitative | Post-intervention | Data collection | TFA-informed questionnaire, adapted or self-developed | Questionnaire | Not specified | Not specified |
| 33 | ACTRN12624001032549 | Outpatient | Unclear | Post-intervention and follow-up | Data collection | TFA-informed questionnaire, adapted or self-developed | Questionnaire | Not specified | Not specified |
| 34 | ACTRN12624000874516 | Community | Quantitative | Post-intervention | Data collection | TFAQ, standardised | Questionnaire | Not specified | Not specified |
| 35 | ACTRN12624000042549 | Community | Unclear | Post-intervention | Data collection | TFA-informed questionnaire, adapted or self-developed | Questionnaire | All constructs | Full |
| 36 | ACTRN12625000591459 | Mixed (Patients; HCPs) | Mixed methods (Interviews; Questionnaire) | Post-intervention | Data collection | Mixed TFA-informed tools | Mixed | Not specified | Not specified |
| 37 | ACTRN12625000378426 | Mixed | Unclear | Post-intervention |  | N/A | Unclear | Not specified | Not specified |
| 38 | ACTRN12625000410459 | Community | Mixed methods (Interviews; TFAQ ) | Post-intervention | Data collection | Mixed TFA-informed tools | Mixed | Not specified | Not specified |
| 39 | NCT03788122 | Inpatient (Patients; Carers) | Qualitative | Multiple timepoints (≥3) | Data collection, Analysis | TFA-informed interview topic guide; Deductive qualitative analysis using TFA constructs | Interviews | Not specified | Not specified |
| 40 | NCT04447508 | Outpatient | Qualitative | Post-intervention | Data collection | TFA-informed interview topic guide | Interviews | Not specified | Not specified |
| 41 | NCT05107440 | Community | Qualitative | Post-intervention | Data collection | TFA-informed interview topic guide | Interviews | Not specified | Not specified |
| 42 | NCT05682183 | Community | Qualitative | Post-intervention | Data collection | TFA-informed interview topic guide | Interviews | Not specified | Not specified |
| 43 | NCT05935020 | Mixed | Qualitative | Post-intervention | Data collection, Analysis | TFA-informed interview topic guide; Deductive qualitative analysis using TFA constructs | Interviews | Not specified | Not specified |
| 44 | NCT05745038 | Community | Quantitative | Post-intervention | Data collection, Analysis | TFAQ, standardised; Descriptive quantitative analysis of TFA constructs | Questionnaire | All constructs | Full |
| 45 | NCT06377709 | Inpatient | Qualitative | Post-intervention | Data collection, Analysis | TFA-informed interview topic guide; Deductive qualitative analysis using TFA constructs | Interviews | Not specified | Not specified |
| 46 | NCT06733103 | Outpatient (Patients; Carers) | Qualitative | Post-intervention | Data collection | TFA-informed interview topic guide | Interviews | Not specified | Not specified |
| 47 | NCT06667739 | Community | Quantitative | Post-intervention | Data collection | TFA-informed questionnaire, adapted or self-developed | Questionnaire | Not specified | Not specified |
| 48 | NCT05182632 | Mixed | Qualitative (Interviews; Focus group) | Post-intervention | Design, Data collection | Mixed TFA-informed tools | Mixed | All constructs | Full |
| 49 | DRKS00034413 | Outpatient | Quantitative | Post-intervention | Data collection | TFA-informed questionnaire, adapted or self-developed | Questionnaire | Not specified | Not specified |
| 50 | NCT04613076 | Outpatient | Quantitative | During and post-intervention | Design, Data collection, Analysis | TFA-informed questionnaire, adapted or self-developed; Descriptive quantitative analysis of TFA constructs | Questionnaire | All constructs | Full |
| 51 | NCT06576557 | Outpatient (Patients; HCPs) | Quantitative | Post-intervention | Data collection | TFA-informed questionnaire, adapted or self-developed | Questionnaire | All constructs | Full |
| 52 | NCT06638918 | Mixed | Quantitative | Post-intervention | Data collection | TFAQ, standardised | Questionnaire | All constructs | Full |
| 53 | NCT06225791 | Outpatient (Patients; HCPs) | Quantitative | During and post-intervention | Data collection | TFAQ, standardised | Questionnaire | Not specified | Not specified |
| 54 | ISRCTN12202900 | Outpatient | Quantitative | Post-intervention | Design, Data collection | TFA-informed questionnaire, adapted or self-developed | Questionnaire | Overall acceptability | Selective |
| 55 | ISRCTN12693443 | Outpatient | Quantitative | Post-intervention | Data collection | TFAQ, standardised | Questionnaire | Not specified | Not specified |
| 56 | ISRCTN23564393 | Inpatient (Patients; HCPs) | Quantitative | Post-intervention | Data collection | TFAQ, standardised | Questionnaire | Not specified | Not specified |
| 57 | ISRCTN16147125 | Mixed (Patients; HCPs) | Quantitative | Post-intervention | Data collection | TFAQ, standardised | Questionnaire | All constructs | Full |
| 58 | ISRCTN16030620 | Outpatient (Patients; Carers; HCPs) | Quantitative | Post-intervention | Data collection | TFA-informed questionnaire, adapted or self-developed | Questionnaire | Not specified | Not specified |
| 59 | ISRCTN86825441 | Community | Mixed methods (Interviews; Questionnaire) | Post-intervention | Data collection, Interpretation | Mixed TFA-informed tools | Mixed | Not specified | Not specified |
| 60 | ISRCTN79060696 | Mixed | Quantitative | Post-intervention | Data collection | TFAQ, standardised | Questionnaire | Not specified | Not specified |
| 61 | ISRCTN13813593 | Community (Patients; HCPs) | Mixed methods (Interviews; TFAQ ) | Post-intervention | Design, Data collection | Mixed TFA-informed tools | Mixed | Not specified | Not specified |
| 62 | ISRCTN32090559 | Outpatient | Quantitative | Post-intervention and follow-up | Data collection | TFAQ, standardised | Questionnaire | Not specified | Not specified |
| 63 | NCT06877273 | Mixed | Quantitative | Unclear | Data collection | TFAQ, standardised | Questionnaire | Overall acceptability | Selective |
| 64 | NCT05840549 | Mixed | Qualitative | Post-intervention | Unclear | N/A | Interviews | Affective attitude; Burden; Ethicality; Coherence; Opportunity cost; Perceived effectiveness | Selective |
| 65 | NCT06114732 | Mixed | Quantitative | Post-intervention | Unclear | N/A | Questionnaire | Not specified | Not specified |
| 66 | NCT06768918 | Mixed | Unclear | Post-intervention | Unclear | N/A | Unclear | Not specified | Not specified |
| 67 | NCT06652828 | Outpatient | Unclear | Post-intervention | Unclear | N/A | Questionnaire | Not specified | Not specified |
| 68 | NCT06983808 | Outpatient | Quantitative | During intervention | Data collection | TFA-informed questionnaire, adapted or self-developed | Questionnaire | All constructs | Full |
| 69 | NCT06143345 | Outpatient | Quantitative | Post-intervention | Data collection | TFA-informed questionnaire, adapted or self-developed | Questionnaire | All constructs | Full |
| 70 | NCT05695170 | Community (Patients; Carers) | Quantitative | Multiple timepoints (≥3) | Data collection | TFA-informed questionnaire, adapted or self-developed | Questionnaire | Not specified | Not specified |
| 71 | NCT06747650 | Community (Patients; Carers) | Quantitative | Post-intervention | Data collection | TFA-informed questionnaire, adapted or self-developed | Questionnaire | All constructs | Full |
| 72 | NCT06001775 | Outpatient (Patients; Carers; HCPs) | Qualitative | Pre- and post-intervention | Unclear | N/A | Interviews | All constructs | Full |
| 73 | NCT04574206 | Community | Qualitative | Post-intervention | Data collection, Analysis | TFA-informed interview topic guide; Deductive qualitative analysis using TFA constructs | Interviews | Not specified | Not specified |
| 74 | NCT06345781 | Community | Mixed methods (Mixed method suvey) | Post-intervention | Data collection | TFA-informed questionnaire, adapted or self-developed | Questionnaire | Not specified | Not specified |
| 75 | NCT06997874 | Outpatient | Mixed methods (Interviews; Questionnaire) | During and post-intervention | Data collection | Mixed TFA-informed tools | Mixed | Affective attitude; Burden; Coherence; Perceived effectiveness; Self-efficacy | Selective |
| 76 | ISRCTN21541209 | Mixed (Patients; HCPs) | Qualitative | Unclear | Data collection | TFA-informed interview topic guide | Interviews | Not specified | Not specified |
| 77 | ISRCTN10420559 | Outpatient | Unclear | Post-intervention | Data collection | TFA-informed questionnaire, adapted or self-developed | Questionnaire | Not specified | Not specified |
| 78 | ISRCTN62558470 | Mixed | Qualitative | Post-intervention | Data collection | TFA-informed focus group topic guide | Focus groups | Not specified | Not specified |
| 79 | ISRCTN17396524 | Outpatient | Mixed methods (Focus group; Questionnaire) | Multiple timepoints (≥3) | Data collection | Mixed TFA-informed tools | Mixed | Perceived effectiveness | Selective |
| 80 | ISRCTN36893888 | Outpatient (Patients; Carers; HCPs) | Qualitative | Post-intervention | Data collection | TFA-informed interview topic guide | Interviews | All constructs | Full |
| 81 | ISRCTN14403521 | Outpatient | Quantitative | Multiple timepoints (≥3) | Data collection, Interpretation | TFAQ, standardised | Questionnaire | All constructs | Full |
| 82 | ISRCTN69024008 | Community (Patients; Carers; HCPs) | Quantitative | Post-intervention | Data collection | TFAQ, standardised | Questionnaire | All constructs | Full |
| 83 | ISRCTN13121835 | Community | Unclear | Post-intervention and follow-up | Data collection | TFA-informed questionnaire, adapted or self-developed | Questionnaire | Affective attitude; Burden; Coherence; Opportunity cost; Perceived effectiveness; Self-efficacy | Selective |
| 84 | NCT05605015 | Community | Quantitative | Post-intervention | Data collection | TFAQ, standardised | Questionnaire | All constructs | Full |
| 85 | NCT05308238 | Community | Unclear | Post-intervention | Data collection | TFA-informed questionnaire, adapted or self-developed | Questionnaire | Not specified | Not specified |
| 86 | NCT05026385 | Community | Qualitative | During and post-intervention | Data collection | Mixed TFA-informed tools | Mixed | Not specified | Not specified |
| 87 | NCT06954532 | Mixed | Quantitative | Post-intervention | Data collection | TFA-informed questionnaire, adapted or self-developed | Questionnaire | Not specified | Not specified |
| 88 | NCT05310565 | Outpatient | Quantitative | Post-intervention | Data collection | TFAQ, standardised | Questionnaire | Not specified | Not specified |
| 89 | NCT04671225 | Outpatient | Unclear | Pre- and post-intervention | Data collection | TFA-informed questionnaire, adapted or self-developed | Questionnaire | Not specified | Not specified |
| 90 | NCT06411223 | Outpatient | Quantitative | Pre- and post-intervention | Data collection | TFAQ, standardised | Questionnaire | Not specified | Not specified |
| 91 | NCT06867757 | Community | Quantitative | Post-intervention | Data collection | TFA-informed questionnaire, adapted or self-developed | Questionnaire | Not specified | Not specified |
| 92 | Bates et al. (2024) | Outpatient | Qualitative | During and post-intervention | Analysis | Deductive qualitative analysis using TFA constructs | Mixed | All constructs | Full |
| 93 | Capobianco et al. (2024) | Outpatient (Patients; Carers; HCPs) | Qualitative | Post-intervention | Analysis | Inductive-deductive qualitative analysis | Mixed | Not specified | Not specified |
| 94 | Graco et al. (2025) | Community (Patients; Carers; HCPs) | Qualitative | Pre- and post-intervention | Data collection, Analysis | TFA-informed interview topic guide; Deductive qualitative analysis using TFA constructs | Interviews | Not specified | Not specified |
| 95 | Johansson et al. (2023) | Mixed (Patients; Carers; HCPs) | Qualitative | Post-intervention | Data collection, Analysis, Interpretation | TFA-informed interview topic guide; Inductive-deductive qualitative analysis | Interviews | All constructs | Full |
| 96 | Malcolm et al. (2025) | Outpatient (Patients; Carers) | Mixed methods (Interviews; Questionnaire) | Multiple timepoints (≥3) | Data collection, Analysis, Interpretation, Reporting | Mixed TFA-informed tools; Parallel mixed-methods analysis (qualitative and quantitative analysed separately) | Mixed | All constructs | Full |
| 97 | Mouton et al. (2025) | Outpatient (Patients; Carers; HCPs) | Qualitative | Post-intervention | Design, Analysis, Interpretation, Reporting | Inductive-deductive qualitative analysis | Interviews | All constructs | Full |
| 98 | Mullenmeister et al. (2024) | Outpatient | Quantitative | Post-intervention | Data collection, Interpretation, Reporting | TFA-informed questionnaire, adapted or self-developed | Questionnaire | All constructs | Full |
| 99 | Swingwood et al. (2023) | Inpatient | Qualitative | Post-intervention | Data collection, Analysis, Interpretation, Reporting | TFA-informed interview topic guide; Deductive qualitative analysis using TFA constructs | Interviews | Not specified | Not specified |
| 100 | Welford et al. (2025) | Inpatient | Quantitative | Post-intervention | Data collection | TFAQ, standardised | Questionnaire | All constructs | Full |
| 101 | Wong et al. (2023) | Outpatient | Qualitative | Multiple timepoints (≥3) | Analysis | Deductive qualitative analysis using TFA constructs | Interviews | Affective attitude; Burden; Ethicality; Coherence; Opportunity cost; Perceived effectiveness | Selective |
| 102 | Arbour et al. (2024) | Outpatient | Quantitative | Pre- and post-intervention | Data collection, Analysis, Interpretation, Reporting | TFA-informed questionnaire, adapted or self-developed; Inferential quantitative analysis of TFA constructs | Questionnaire | All constructs | Full |
| 103 | Barnett et al. (2024) | Outpatient | Mixed methods (Interviews; Questionnaire) | Post-intervention | Analysis, Interpretation, Reporting | Integrated mixed-methods TFA analysis | Mixed | All constructs | Additional constructs introduced |
| 104 | Beales et al. (2024) | Community | Mixed methods (Interviews; Questionnaire) | Post-intervention | Analysis | Integrated mixed-methods TFA analysis | Mixed | All constructs | Full |
| 105 | Browne et al. (2025) | Community | Qualitative | Post-intervention | Design, Data collection, Analysis, Reporting | TFA-informed interview topic guide; Deductive qualitative analysis using TFA constructs | interviews | Affective attitude; Burden; Perceived effectiveness; Self-efficacy | Selective |
| 106 | Bull et al. (2025) | Outpatient (Patients; HCPs) | Qualitative | Pre- and post-intervention | Analysis | Inductive-deductive qualitative analysis | Mixed | Not specified | Not specified |
| 107 | Bullock et al. (2024) | Outpatient | Qualitative | During intervention | Data collection, Analysis, Reporting | TFA-informed interview topic guide; Inductive-deductive qualitative analysis | Interviews | All constructs | Full |
| 108 | Casmir et al. (2025) | Outpatient (Patients; HCPs) | Qualitative | During intervention | Analysis, Reporting | Inductive-deductive qualitative analysis | Mixed | Affective attitude; Burden; Ethicality; Perceived effectiveness; Self-efficacy | Selective |
| 109 | Chen et al. (2025) | Community | Mixed methods (Focus group; Interviews; Questionnaire) | Multiple timepoints (≥3) | Analysis, Reporting | Parallel mixed-methods analysis (qualitative and quantitative analysed separately) | Mixed | All constructs | Full |
| 110 | Chew-Graham et al. (2022) | Community (Patients; HCPs) | Qualitative | Post-intervention | Analysis, Reporting | Inductive-deductive qualitative analysis | Mixed | Affective attitude; Burden; Coherence; Opportunity cost; Perceived effectiveness; Self-efficacy | Selective |
| 111 | Cleland et al. (2025) | Outpatient (Patients; Carers) | Qualitative | Post-intervention | Analysis, Reporting | Inductive-deductive qualitative analysis | Mixed | All constructs | Full |
| 112 | Cornwall et al. (2024) | Community (Patients; HCPs) | Qualitative | Pre- and post-intervention | Design, Data collection, Analysis | TFA-informed interview topic guide; Deductive qualitative analysis using TFA constructs | Interviews | All constructs | Full |
| 113 | Davis et al. (2024) | Community | Qualitative | Post-intervention | Design, Data collection, Analysis, Reporting | TFA-informed interview topic guide; Inductive-deductive qualitative analysis | Interviews | All constructs | Full |
| 114 | Dedic et al. (2025) | Mixed | Mixed methods (Interviews; Questionnaire) | Post-intervention | Analysis, Reporting | Integrated mixed-methods TFA analysis | Mixed | All constructs | Full |
| 115 | Deegan et al. (2024) | Community | Qualitative | Post-intervention | Analysis, Reporting | Deductive qualitative analysis using TFA constructs | Mixed | All constructs | Full |
| 116 | Enoch et al. (2023) | Inpatient | Qualitative | Post-intervention | Analysis, Reporting | Deductive qualitative analysis using TFA constructs | Interviews | All constructs | Full |
| 117 | Gaboury et al. (2022) | Community (Patients; HCPs) | Qualitative | Post-intervention | Data collection, Analysis, Reporting | Mixed TFA-informed tools; Inductive-deductive qualitative analysis | Mixed | All constructs | Full |
| 118 | Gerards et al. (2022) | Outpatient | Qualitative | Post-intervention | Data collection, Analysis, Reporting | TFA-informed interview topic guide; Deductive qualitative analysis using TFA constructs | Interviews | All constructs | Full |
| 119 | Gerbild et al. (2021) | Community | Qualitative | Pre-intervention | Design, Data collection, Analysis, Interpretation, Reporting | TFA-informed interview topic guide; Deductive qualitative analysis using TFA constructs | Interviews | All constructs | Full |
| 120 | Green et al. (2025) | Outpatient (Patients; HCPs) | Qualitative | Post-intervention | Design, Data collection, Analysis, Interpretation, Reporting | TFA-informed interview topic guide; Deductive qualitative analysis using TFA constructs | Interviews | All constructs | Full |
| 121 | Green et al. (2024) | Outpatient (Patients; HCPs) | Mixed methods (Interviews; Questionnaire) | Post-intervention | Design, Data collection, Analysis, Interpretation, Reporting | Mixed TFA-informed tools; Parallel mixed-methods analysis (qualitative and quantitative analysed separately) | Mixed | Affective attitude; Burden; Coherence; Perceived effectiveness; Overall acceptability | Selective |
| 122 | Griffey et al. (2025) | Inpatient | Quantitative | Post-intervention | Design, Data collection, Analysis, Interpretation, Reporting | TFAQ, standardised; Descriptive quantitative analysis of TFA constructs | Questionnaire | All constructs | Full |
| 123 | Groarke et al. (2021) | Outpatient | Mixed methods (Interviews; Questionnaire) | Post-intervention | Design, Data collection, Analysis, Interpretation, Reporting | Mixed TFA-informed tools; Parallel mixed-methods analysis (qualitative and quantitative analysed separately) | Mixed | Affective attitude; Burden; Coherence; Perceived effectiveness; Self-efficacy | Selective |
| 124 | Harris et al. (2023) | Mixed | Qualitative | Post-intervention | Design, Data collection, Analysis, Interpretation, Reporting | TFA-informed interview topic guide; Deductive qualitative analysis using TFA constructs | Interviews | Affective attitude; Burden; Coherence; Perceived effectiveness; Self-efficacy | Selective |
| 125 | Hendry et al. (2022) | Outpatient (Patients; HCPs) | Qualitative | Post-intervention | Design, Data collection, Analysis, Interpretation, Reporting | TFA-informed interview topic guide; Deductive qualitative analysis using TFA constructs | Interviews | Affective attitude; Burden; Coherence; Opportunity cost; Perceived effectiveness; Self-efficacy; | Selective |
| 126 | Heneka et al. (2023) | Outpatient (Patients; HCPs) | Qualitative | Post-intervention | Design, Data collection, Analysis, Interpretation, Reporting | TFA-informed interview topic guide; Deductive qualitative analysis using TFA constructs | Interviews | All constructs | Full |
| 127 | Herdman et al. (2022) | Outpatient (Patients; HCPs) | Quantitative | Post-intervention | Interpretation, Reporting | N/A | Questionnaire | All constructs | Full |
| 128 | Herron et al. (2021) | Outpatient | Qualitative | Post-intervention | Analysis, Interpretation, Reporting | Inductive-deductive qualitative analysis | Interviews | Affective attitude; Burden; Coherence; Opportunity cost; Perceived effectiveness; Self-efficacy; | Full |
| 129 | Istanboulian et al. (2023) | Inpatient (Patients; Carers; HCPs) | Qualitative | Post-intervention | Design, Analysis, Interpretation, Reporting | Deductive qualitative analysis using TFA constructs | Interviews | All constructs | Full |
| 130 | Jafar et al. (2024) | Community (Patients; HCPs) | Qualitative | Post-intervention | Design, Data collection, Analysis, Interpretation, Reporting | TFA-informed interview topic guide; Deductive qualitative analysis using TFA constructs | Interviews | All constructs | Full |
| 131 | Jafar et al. (2023) | Outpatient (Patients; HCPs) | Qualitative | Post-intervention | Design, Data collection, Analysis, Interpretation, Reporting | TFA-informed interview topic guide; Inductive-deductive qualitative analysis | Interviews | All constructs | Full |
| 132 | Keyworth et al. (2021) | Community | Qualitative | Post-intervention | Design, Data collection, Analysis, Interpretation, Reporting | TFA-informed questionnaire, adapted or self-developed; Deductive qualitative analysis using TFA constructs | Questionnaire | All constructs | Full |
| 133 | Keyworth et al. (2022) | Community | Qualitative | Post-intervention | Design, Data collection, Analysis, Interpretation, Reporting | TFA-informed interview topic guide; Deductive qualitative analysis using TFA constructs | interviews | Affective attitude; Burden; Coherence; Perceived effectiveness; | Selective |
| 134 | Keyworth et al. (2023) | Community | Quantitative | Pre- and post-intervention | Design, Data collection, Analysis, Interpretation, Reporting | TFA-informed questionnaire, adapted or self-developed; Inferential quantitative analysis of TFA constructs | Questionnaire | All constructs | Full |
| 135 | Kiirya et al. (2025) | Community (Patients; HCPs) | Qualitative | Pre-intervention | Analysis | Deductive qualitative analysis using TFA constructs | Interviews | All constructs | Full |
| 136 | Kuruppu et al. (2024) | Inpatient (Patients; HCPs) | Qualitative | Pre-intervention | Design, Analysis, Interpretation | Inductive-deductive qualitative analysis | Interviews | Affective attitude; Burden; Coherence; Opportunity cost; Perceived effectiveness; Self-efficacy | Selective |
| 137 | Liptrott et al. (2022) | Outpatient | Qualitative | Pre-intervention | Analysis, Interpretation, Reporting | Inductive-deductive qualitative analysis | Mixed | All constructs | Full |
| 138 | Liptrott et al. (2020) | Outpatient | Qualitative | Post-intervention | Analysis, Interpretation | Inductive-deductive qualitative analysis | Interviews | All constructs | Full |
| 139 | Locke et al. (2020) | Outpatient | Qualitative | Post-intervention | Analysis, Interpretation, Reporting | Deductive qualitative analysis using TFA constructs | Interviews | All constructs | Full |
| 140 | Lopes et al. (2025) | Outpatient | Qualitative | Post-intervention | Design, Data collection, Analysis, Interpretation, Reporting | TFA-informed interview topic guide; Deductive qualitative analysis using TFA constructs | Interviews | All constructs | Full |
| 141 | Manduchi et al. (2024) | Outpatient | Qualitative | Post-intervention | Design, Data collection, Analysis, Interpretation, Reporting | TFA-informed interview topic guide; Inductive-deductive qualitative analysis | Interviews | All constructs | Full |
| 142 | McIlroy et al. (2022) | Outpatient (Patients; HCPs) | Qualitative | Post-intervention | Interpretation, Reporting | N/A | Mixed | Affective attitude; Burden; Coherence; Perceived effectiveness; Self-efficacy | Selective |
| 143 | Morris et al. (2023) | Community (Patients; Carers) | Qualitative | Post-intervention | Analysis, Interpretation, Reporting | Inductive-deductive qualitative analysis | Interviews | All constructs | Full |
| 144 | Murphy et al. (2022) | Outpatient | Qualitative | Post-intervention | Analysis, Interpretation, Reporting | Inductive-deductive qualitative analysis | Interviews | All constructs | Full |
| 145 | Nguyen et al. (2024) | Outpatient | Qualitative | Pre-intervention | Design, Data collection, Analysis, Interpretation, Reporting | TFA-informed interview topic guide; Deductive qualitative analysis using TFA constructs | Interviews | Affective attitude; Burden; Ethicality; Opportunity cost; Perceived effectiveness; Self-efficacy | Selective |
| 146 | Pattison et al. (2022) | Community | Qualitative | During and post-intervention | Analysis, Interpretation, Reporting | Inductive-deductive qualitative analysis | Questionnaire | All constructs | Full |
| 147 | Pearsons et al. (2025) | Outpatient (Patients; HCPs) | Qualitative | Post-intervention | Analysis, Interpretation, Reporting | Inductive-deductive qualitative analysis | interviews | All constructs | Full |
| 148 | Pedley et al. (2020) | Outpatient | Qualitative | Post-intervention | Analysis, Interpretation, Reporting | Inductive-deductive qualitative analysis | interviews | All constructs | Full |
| 149 | Rizzo et al. (2023) | #N/A | Qualitative | Post-intervention | Analysis, Interpretation, Reporting | Inductive-deductive qualitative analysis | interviews | All constructs | Full |
| 150 | Roponen et al. (2025) | Mixed | Quantitative | Post-intervention | Data collection, Analysis, Interpretation, Reporting | TFA-informed questionnaire, adapted or self-developed; Descriptive quantitative analysis of TFA constructs | Questionnaire | All constructs | Full |
| 151 | Rushton et al. (2020) | Community | Qualitative | Post-intervention | Data collection, Analysis, Interpretation, Reporting | TFA-informed interview topic guide; Inductive-deductive qualitative analysis | Interviews | All constructs | Full |
| 152 | Sara et al. (2025) | Community (Patients; HCPs) | Qualitative | Post-intervention | Design, Data collection, Analysis, Interpretation, Reporting | TFA-informed interview topic guide; Deductive qualitative analysis using TFA constructs | Interviews | All constructs | Full |
| 153 | Sharrad et al. (2023) | Outpatient (Patients; Carers; HCPs) | Qualitative | During intervention | Analysis, Interpretation, Reporting | Deductive qualitative analysis using TFA constructs | Interviews | All constructs | Full |
| 154 | Shearsmith et al. (2022) | Community | Qualitative | Post-intervention | Data collection, Analysis, Interpretation, Reporting | TFA-informed interview topic guide; Inductive-deductive qualitative analysis | Interviews | All constructs | Full |
| 155 | Simelane et al. (2025) | Outpatient (Patients; HCPs) | Qualitative | During intervention | Data collection, Analysis, Interpretation, Reporting | TFA-informed interview topic guide; Deductive qualitative analysis using TFA constructs | Interviews | All constructs | Full |
| 156 | Smyth et al. (2024) | Mixed (Patients; Carers; HCPs) | Mixed methods (Interviews; Questionnaire) | Pre-intervention | Design, Data collection, Analysis, Interpretation, Reporting | Mixed TFA-informed tools; Parallel mixed-methods analysis (qualitative and quantitative analysed separately) | Mixed | All constructs | Full |
| 157 | Sohanpal et al. (2024) | Community | Qualitative | Post-intervention | Data collection, Interpretation | TFA-informed interview topic guide | Interviews | Affective attitude; Burden; Coherence; Opportunity cost; Perceived effectiveness; Self-efficacy | Selective |
| 158 | Timm et al. (2022) | Community | Quantitative | Post-intervention | Design, Data collection, Analysis, Interpretation, Reporting | TFA-informed questionnaire, adapted or self-developed; Descriptive quantitative analysis of TFA constructs | Questionnaire | All constructs | Full |
| 159 | Tripathee et al. (2025) | Community | Qualitative | Pre- and post-intervention | Analysis, Interpretation, Reporting | Inductive-deductive qualitative analysis | Mixed | Affective attitude; Burden; Coherence; Opportunity cost; Perceived effectiveness; Self-efficacy | Selective |
| 160 | Vergeer-Hagoort et al. (2024) | Outpatient | Qualitative | Post-intervention | Analysis, Interpretation, Reporting | Deductive qualitative analysis using TFA constructs | Interviews | All constructs | Full |
| 161 | Volkmer et al. (2024) | Mixed | Qualitative | Post-intervention | Interpretation, Reporting | N/A | Interviews | Affective attitude; Burden; Coherence; Opportunity cost; Perceived effectiveness; Self-efficacy | Selective |
| 162 | Weemaes et al. (2025) | Community | Qualitative | Post-intervention | Design, Data collection, Analysis, Interpretation, Reporting | TFA-informed interview topic guide; Inductive-deductive qualitative analysis | Interviews | All constructs | Full |
| 163 | Weinryb et al. (2025) | Outpatient | Mixed methods (Interviews; Questionnaire) | Post-intervention | Data collection, Analysis, Interpretation, Reporting | Mixed TFA-informed tools; Integrated mixed-methods TFA analysis | Mixed | All constructs | Full |
| 164 | Western et al. (2023) | Outpatient | Mixed methods (Interviews; Questionnaire) | Post-intervention | Design, Data collection, Analysis, Interpretation, Reporting | TFAQ, standardised; Parallel mixed-methods analysis (qualitative and quantitative analysed separately) | Questionnaire | All constructs | Full |
| 165 | Deja et al. (2021) | Inpatient | Qualitative | Pre + Post | Analysis, Interpretation, Reporting | Deductive qualitative analysis using TFA constructs | Interviews | All constructs | Full |

# Additional File 9: Sensitivity analysis restricted to empirical studies

**Table S1:** Conceptualisation, assessment and engagement of acceptability among empirical studies

| **Domain** | **Number of empirical studies (n=64)** | **Percentage (%)** | **Domain** | **Number of empirical studies (n=64)** | **Percentage (%)** |
| --- | --- | --- | --- | --- | --- |
| **Type of TFA data collected** | | | **Population (TFA-measured)** | | |
| Qualitative | **49** | **76.6%** | Patients / service users only | **37** | **57.8%** |
| Quantitative | **6** | **9.4%** | Healthcare professionals | **6** | **9.4%** |
| Mixed methods | **9** | **14.1%** | Carers / informal support only | **0** | **0%** |
| Not reported | **0** | **0%** | Multiple stakeholder groups | **21** | **32.8%** |
| **Method used to assess acceptability** | | | **Positioning of acceptability** | | |
| Questionnaire | **9** | **14.1%** | Outcome | **42** | **65.6%** |
| Interviews | **38** | **59.4%** | Process outcome | **15** | **23.4%** |
| Combined | **17** | **26.6%** | Combined (outcome + process) | **7** | **10.9%** |
| Focus groups | **0** | **0%** | **Timing of assessment** | | |
| Not reported | **0** | **0%** | Pre-intervention | **6** | **9.4%** |
| **Construct engagement** | | | During intervention | **4** | **6.3%** |
| Full set of 7 constructs | **38** | **59.4%** | Post-intervention | **46** | **71.9%** |
| Full + overall acceptability | **10** | **15.6%** | Pre + Post | **6** | **9.4%** |
| Selective use | **14** | **21.9%** | During + Post | **1** | **1.6%** |
| Additional constructs | **1** | **1.6%** | Post + follow-up | **0** | **0%** |
| Not specified | **1** | **1.6%** | Multiple stages (3+) | **1** | **1.6%** |
|  |  |  | Not reported | **0** | **0%** |

**Table S2:** Application of the TFA across the research process among empirical studies

| **Stage of TFA application** | **Number of studies** | **Percentage (%)** |
| --- | --- | --- |
| **TFA used to inform study design** | **26/64** | **40.6%** |
| **TFA used to inform data collection** | **34/64** | **53.1%** |
| Interview guides informed by the TFA | **22/34** | **64.7%** |
| Focus group topic guides informed by the TFA | **0/34** | **0%** |
| Study-specific questionnaires informed by the TFA | **5/34** | **14.7%** |
| Standardised TFAQ | **2/34** | **5.9%** |
| Multiple data collection tools informed by the TFA | **5/34** | **14.7%** |
| **TFA used to inform analysis** | **60/64** | **93.8%** |
| Deductive qualitative analysis | **23/60** | **38.3%** |
| Inductive-deductive qualitative analysis | **23/60** | **38.3%** |
| Descriptive quantitative analysis | **3/60** | **5.0%** |
| Inferential quantitative analysis | **2/60** | **3.3%** |
| Parallel mixed-methods analysis | **5/60** | **8.3%** |
| Integrated mixed-methods analysis | **4/60** | **6.7%** |
| **TFA used to inform interpretation** | **48/64** | **75.0%** |
| **TFA used to structure reporting** | **57/64** | **89.1%** |
| **Unclear - Not reported where TFA applied** | **0/64** | **0%** |

# Additional File 10: Mapping of review findings to the proposed TFA reporting checklist

This table provides an item-level account of how the proposed evidence-informed reporting checklist was derived from the pooled synthesis of 165 study entries and the post hoc sensitivity analysis restricted to 64 empirical studies. The final column explains how the identified pattern informed the corresponding reporting recommendation. The checklist was derived from the findings of this review and has not undergone formal consensus testing or external validation.

| **Checklist item and recommendation** | **Review findings underpinning the item** | **How the findings informed the reporting recommendation** |
| --- | --- | --- |
| **Purpose and rationale for TFA use** Explain the purpose of using the TFA and how its use relates to the study aim, intervention context and positioning of acceptability. | **Pooled findings:** The TFA was used for different purposes and applied at different stages of the research process. Study entries varied in whether acceptability was positioned as an outcome, a process outcome or both; when it was assessed; which constructs were selected; and how the framework informed design, data collection, analysis, interpretation and reporting.  **Empirical sensitivity:** Variation remained among empirical studies in the positioning and timing of acceptability assessment, construct selection and the stages at which the TFA was applied. | Because the TFA was used for different purposes and in different ways, readers need an explicit explanation of why the framework was selected and how it relates to the study aim, intervention context and positioning of acceptability. This provides the conceptual basis for interpreting subsequent decisions about timing, construct selection, measurement and framework integration. |
| **Positioning of acceptability**  State whether acceptability is treated as an outcome, a process outcome, or both. | **Pooled findings:** Acceptability was predominantly positioned as an outcome of intervention exposure (118/165, 71.5%), with fewer studies positioning it as a process outcome (27/165, 16.4%) or both an outcome and a process outcome (20/165, 12.1%).  **Empirical sensitivity:** Outcome positioning remained most common among empirical studies (42/64, 65.6%), although process-oriented use was more frequent than in the pooled analysis (15/64, 23.4%); 7/64 (10.9%) used both positions. | The role assigned to acceptability shapes the purpose of assessment and the meaning of the findings. Explicitly reporting whether acceptability is an outcome, a process outcome or both enables readers to understand whether the study is evaluating a post-exposure judgement, an evolving experience, or both. |
| **Stage of integration**  Specify at which stages the TFA is applied: design, data collection, analysis, interpretation and/or reporting. | **Pooled findings:** The TFA was used to inform study design in 32/165 (19.4%), data collection in 125/165 (75.8%), analysis in 75/165 (45.5%), interpretation in 56/165 (33.9%) and reporting in 61/165 (37.0%). Five trial registrations did not clearly report the stage of application. Categories were not mutually exclusive.  **Empirical sensitivity:** Among empirical studies, the TFA was most frequently used during analysis (60/64, 93.8%), reporting (57/64, 89.1%) and interpretation (48/64, 75.0%). It informed data collection in 34/64 (53.1%) and study design in 26/64 (40.6%). | The pooled and empirical findings showed that planned and completed applications differed and that framework use often extended across multiple stages. Reporting each stage separately makes it possible to distinguish intended from implemented use and to assess whether the TFA was integrated throughout the research process or applied only at selected points. |
| **Stakeholder inclusion**  Report whose perspectives are assessed, such as intervention recipients, deliverers, carers or other stakeholders, and justify their inclusion. | **Pooled findings:** Acceptability was assessed among patients or service users only in 102/165 (61.8%), healthcare professionals only in 6/165 (3.6%), carers or informal supporters only in 3/165 (1.8%) and multiple stakeholder groups in 54/165 (32.7%).  **Empirical sensitivity:** Among empirical studies, 37/64 (57.8%) included patients or service users only, 6/64 (9.4%) included healthcare professionals only and 21/64 (32.8%) included multiple stakeholder groups. No empirical study assessed carers or informal supporters alone. | The concentration on patient perspectives and limited standalone attention to carers or deliverers may leave important dimensions of intervention delivery, burden and sustainment unexplored. Authors should identify whose acceptability is being assessed and explain why those perspectives are relevant to the intervention and implementation context. |
| **Timing of assessment**  Report when acceptability is assessed, including pre-intervention, during intervention, post-intervention and follow-up, and provide a rationale. | **Pooled findings:** Assessment most commonly occurred post-intervention (116/165, 70.3%). Fewer studies assessed acceptability pre-intervention (6/165, 3.6%), during intervention (7/165, 4.2%), at pre- and post-intervention timepoints (13/165, 7.9%), during and post-intervention (6/165, 3.6%), post-intervention and follow-up (3/165, 1.8%) or across three or more stages (9/165, 5.5%); timing was not reported in 5/165 (3.0%).  **Empirical sensitivity:** Post-intervention assessment remained predominant (46/64, 71.9%). Six empirical studies assessed acceptability pre-intervention, four during intervention, six pre- and post-intervention, one during and post-intervention and one across three or more stages. | Because anticipated, concurrent and experienced acceptability capture different judgements, timing determines what the assessment can show. Explicit timing and rationale allow readers to interpret whether findings concern expectations, evolving experiences, retrospective evaluations or change over time. |
| **Construct selection**  Specify which TFA constructs are included and justify any omissions, additions or adaptations. | **Pooled findings:** Fifty-eight of 165 studies used all seven constructs, 18/165 used all seven plus overall acceptability, 22/165 used selected constructs and one added a construct. Among studies using selected constructs, ethicality was omitted in 15/22 and opportunity cost in 10/22. Reasons for omission were rarely reported. Construct use was not specified in 66/165 entries, concentrated among trial registrations and protocols.  **Empirical sensitivity:** Among empirical studies, 38/64 used all seven constructs, 10/64 used all seven plus overall acceptability, 14/64 used selected constructs and one added a construct. Only one empirical study did not specify construct use. | Selective use may be appropriate, but without a clear rationale readers cannot judge whether omissions reflect conceptual relevance, population needs, measurement constraints or incomplete application. Reporting the constructs assessed and explaining omissions or adaptations supports interpretability and comparison across studies. |
| **Operationalisation**  Describe how TFA constructs informed data collection tools and study procedures. | **Pooled findings:** The TFA informed data collection in 125/165 studies. Within these, it informed interview guides in 39/125, a focus group guide in 1/125, study-specific questionnaires in 42/125, the standardised TFAQ in 25/125 and multiple tools in 18/125. The explicit linkage between constructs and questionnaire items or interview content was not always clearly reported.  **Empirical sensitivity:** The TFA informed data collection in 34/64 empirical studies: interview guides in 22/34, study-specific questionnaires in 5/34, the standardised TFAQ in 2/34 and multiple tools in 5/34. This demonstrated variation in how TFA constructs were translated into data collection methods within completed studies. | The framework was translated into several different tools and procedures. Describing the route from each construct to the corresponding question, item or study activity enables readers to assess whether constructs were operationalised coherently and supports replication or appropriate adaptation. |
| **Measurement approach**  Report the measures or methods used, such as the TFAQ, adapted questionnaires, interviews or combined approaches, and explain how they were developed or selected. | **Pooled findings:** Questionnaires were the most commonly reported or planned assessment method (71/165, 43.0%), followed by interviews (59/165, 35.8%), combined approaches (32/165, 19.4%) and focus groups (1/165, 0.6%); the method was not reported in two entries. TFA data were qualitative in 76/165, quantitative in 52/165 and mixed methods in 21/165, with type not reported in 16/165.  **Empirical sensitivity:** Empirical studies were predominantly qualitative (49/64, 76.6%) and interview-based (38/64, 59.4%). Nine of 64 (14.1%) relied on questionnaires and 17/64 (26.6%) used combined approaches. Six empirical studies collected quantitative data and nine used mixed methods. | The differences between planned questionnaire use and completed interview-based research, together with variation in the use of standardised, study-specific and combined methods, show that the assessment approach cannot be inferred from a statement that the TFA was used. Detailed reporting permits appraisal of method suitability and comparison of findings generated through different approaches. |
| **Analytical approach**  Describe how the TFA informed analysis, including deductive, inductive-deductive, descriptive or inferential quantitative, and mixed-methods approaches. | **Pooled findings:** The TFA informed analysis in 75/165 studies. These included deductive qualitative analysis (31/75, 41.3%), inductive-deductive qualitative analysis (26/75, 34.7%), descriptive quantitative analysis (6/75, 8.0%), inferential quantitative analysis (2/75, 2.7%), parallel mixed-methods analysis (6/75, 8.0%) and integrated mixed-methods analysis (4/75, 5.3%).  **Empirical sensitivity:** The TFA informed analysis in 60/64 empirical studies. Deductive and inductive-deductive qualitative approaches were equally common (23/60 each, 38.3%). Three studies used descriptive quantitative analysis, two used inferential quantitative analysis, five used parallel mixed-methods analysis and four used integrated mixed-methods analysis. | Substantively different analytic procedures were grouped under the broad description of TFA-informed analysis. Naming the procedure and explaining how the framework entered the analysis allows readers to judge analytic coherence, understand whether findings were framework-led or data-led and compare results appropriately. |
| **Interpretation and application**  Explain how acceptability findings are interpreted and used in relation to intervention refinement, implementation or study conclusions. | **Pooled findings:** The TFA was explicitly used to inform interpretation in 56/165 studies (33.9%). Across the evidence base, inconsistent construct use and limited linkage between construct-level findings and intervention outcomes prevented robust cumulative conclusions about which aspects of acceptability influence uptake, engagement, fidelity or effectiveness.  **Empirical sensitivity:** Interpretive use was evident in 48/64 empirical studies (75.0%), showing that completed studies frequently used the TFA to make sense of their findings. However, variation in construct use and the limited cumulative linkage between construct-level findings and intervention or implementation outcomes restricted conclusions about which aspects of acceptability matter, for whom and under what conditions. | Because interpretive use was common among empirical studies, authors should make explicit how the TFA shaped the meaning attributed to findings and whether those findings informed intervention refinement, implementation decisions or study conclusions. This distinguishes the description of acceptability from interpretation of its practical implications and supports cumulative learning. |
| **Reporting transparency**  Provide sufficient detail to demonstrate how the TFA was applied across all relevant stages of the study. | **Pooled findings:** The TFA explicitly structured reporting in 61/165 studies (37.0%). Five trial registrations did not state where the framework was applied. Construct use was unspecified in 66/165 entries, including 62/91 trial registrations and 3/10 protocols, and the rationale for selective construct omission and the mapping of constructs to tools were not always reported.  **Empirical sensitivity:** The TFA structured reporting in 57/64 empirical studies (89.1%), and only one empirical study did not specify construct use. However, empirical studies continued to vary in positioning, timing, construct selection, operationalisation and analytical approach. Structuring findings according to TFA constructs did not necessarily provide complete information about how or why the framework was applied at each stage. | Some apparent non-reporting in the pooled synthesis reflected the limited methodological detail available in registry records rather than empirical practice. Nevertheless, the heterogeneity that remained among empirical studies means that readers require sufficient detail across each relevant dimension to understand the study’s methodological choices. Complete reporting supports critical appraisal, replication and appropriate comparison without implying that all variation represents inadequate practice. |
